# High-throughput quantitative binding analysis of DNA aptamers using exonucleases

**DOI:** 10.1093/nar/gkac1210

**Published:** 2022-12-30

**Authors:** Juan Canoura, Obtin Alkhamis, Yingzhu Liu, Connor Willis, Yi Xiao

**Affiliations:** Department of Chemistry, North Carolina State University, 2620 Yarbrough Drive, Raleigh, NC 27607, USA; Department of Chemistry and Biochemistry, Florida International University, 11200 SW 8th Street, Miami, FL 33199, USA; Department of Chemistry, North Carolina State University, 2620 Yarbrough Drive, Raleigh, NC 27607, USA; Department of Chemistry, North Carolina State University, 2620 Yarbrough Drive, Raleigh, NC 27607, USA; Department of Chemistry, North Carolina State University, 2620 Yarbrough Drive, Raleigh, NC 27607, USA; Department of Chemistry, North Carolina State University, 2620 Yarbrough Drive, Raleigh, NC 27607, USA; Department of Chemistry and Biochemistry, Florida International University, 11200 SW 8th Street, Miami, FL 33199, USA

## Abstract

Aptamers are nucleic acid bioreceptors that have been used in various applications including medical diagnostics and as therapeutic agents. Identifying the most optimal aptamer for a particular application is very challenging. Here, we for the first time have developed a high-throughput method for accurately quantifying aptamer binding affinity, specificity, and cross-reactivity via the kinetics of aptamer digestion by exonucleases. We demonstrate the utility of this approach by isolating a set of new aptamers for fentanyl and its analogs, and then characterizing the binding properties of 655 aptamer–ligand pairs using our exonuclease digestion assay and validating the results with gold-standard methodologies. These data were used to select optimal aptamers for the development of new sensors that detect fentanyl and its analogs in different analytical contexts. Our approach dramatically accelerates the aptamer characterization process and streamlines sensor development, and if coupled with robotics, could enable high-throughput quantitative analysis of thousands of aptamer–ligand pairs.

## INTRODUCTION

Aptamers are short DNA or RNA molecules that can bind to targets with high affinity and specificity (1,2). They have several distinctive properties that make them ideal for biosensing, including low cost, ease of chemical synthesis and modification, high stability and long shelf-life (3,4). These nucleic acid-based receptors are being increasingly applied for a wide variety of applications including therapeutic agents (5), medical diagnostics (6), real-time *in vivo* drug monitoring (7) and fundamental biological research in fields such as pharmacology (8) and neuroscience (9). Aptamers are isolated from random oligonucleotide libraries through an *in vitro* method termed systematic evolution of ligands by exponential enrichment (SELEX) (1,2). Starting with a library of ∼10^15^ unique sequences, a typical SELEX screen yields a final pool of 10^3^–10^5^ sequences after multiple rounds of selection. Currently, it is prohibitively difficult to select the most appropriate aptamer for a particular application from this vast number of candidates. Determining the aptamer sequence with the most desirable binding profile requires comprehensive, accurate characterization of the binding properties of many individual candidates, and this step remains challenging and inefficient.

Conventional affinity determination methodologies such as isothermal titration calorimetry (ITC), microscale thermophoresis and surface plasmon resonance have low throughput and require specialized instrumentation (10–12). There are some high-throughput assays that provide information on the binding characteristics for numerous aptamer–ligand pairs (13–15). For example, strand-displacement fluorescence assays have been widely used to characterize aptamer binding affinity and specificity (16,17). However, this assay requires chemically-labeled oligonucleotides, which limits the number of sequences that can be characterized due to the high cost of synthesis. Alternatively, DNA microarray technologies have been adapted for high-throughput aptamer affinity screening (18,19). For example, the Soh group determined the binding affinity of hundreds of aptamers from a series of SELEX rounds simultaneously by synthesizing the top candidates from enriched pools on microarrays and challenging them with fluorophore-labeled protein target (20). However, microarray-based methods are expensive and generally difficult to apply to small-molecule targets, as these typically have peak fluorescence emission <300 nm, and fluorophore labeling can alter their binding properties.

We have recently developed a label-free, solution-phase fluorescence assay based on exonuclease III (Exo III) and exonuclease I (Exo I) to identify the specific binding of DNA aptamers to small molecules and proteins (21,22). In this assay, unbound aptamers are rapidly digested into mononucleotides by these exonucleases, but target-bound aptamers resist enzyme digestion to an extent that is dependent on the concentration of target present, leaving behind a digestion product that retains affinity for its target. Although we observed a qualitative relationship between enzymatic inhibition and aptamer target-binding affinity, the dataset was too small to establish a definitive, quantitative correlation (21). Moreover, we only applied this assay to well-studied aptamers with known three-dimensional structures.

In this work, we have demonstrated that the kinetics of aptamer digestion by exonucleases can be used to quantitatively assess the affinity and specificity of an aptamer digestion product for small-molecule targets as a starting point for biosensor development. As a demonstration, we focused on the small-molecule drug fentanyl and its analogs. Fentanyl is a potent narcotic analgesic that works by activating μ-opioid receptors (23), and as of 2021, fentanyl and its analogs are the fourth most abused illicit substances and the top cause of drug-related overdose deaths in the USA (24,25). Currently, detecting fentanyl involves the use of chemical spot tests, which are inexpensive and rapid but have poor specificity, and lateral-flow immunoassays, which although can detect fentanyl and a few analogs, are unable to detect many other analogs, of which 45 have been identified thus far (26,27). There is therefore a critical need for high-affinity bioreceptors that are specifically cross-reactive to fentanyl and its analogs, and which can be employed for sensitive presumptive testing for this synthetic opioid family. In response to this need, here we isolated 28 aptamer candidates from a SELEX screen against fentanyl, acetyl fentanyl, and furanyl fentanyl and characterized their binding properties using our label-free exonuclease digestion assay and validated our results with gold-standard methodologies such as ITC and the strand-displacement fluorescence assay. We observed an inverse correlation between the resistance of an aptamer to exonuclease digestion in the presence of its target and the equilibrium dissociation constant (*K*_D_) of the aptamer digestion product. We also used our assay to accurately identify the specificity of several aptamers towards 19 interferent molecules as well as 14 fentanyl analogs. Our overall validation process, encompassing 655 aptamer–ligand pairs, provides high confidence in the accuracy and precision of this assay. Having amassed a plethora of data on the binding properties of the isolated aptamers, we were able to identify optimal aptamers for use in an electrochemical aptamer-based (E-AB) sensor that can sensitively detect nanomolar concentrations of fentanyl in biological samples as well as a presumptive E-AB assay that can detect fentanyl and 14 of its analogs in binary mixtures designed to mimic seized samples without responding to unrelated interferents or cutting agents. Given that our exonuclease digestion method can be broadly applied, independent of aptamer sequence and structure or ligand physicochemical properties, we believe this assay should provide a general means for rapidly and quantitatively determining the binding characteristics of many aptamers in a high-throughput manner.

## MATERIALS AND METHODS

### Chemicals and reagents

Exonuclease III (*Escherichia coli*) (100 U/μl) and Exonuclease I (*E. coli*) (20 U/μl) were purchased from New England Biolabs. Fentanyl, acetyl fentanyl, furanyl fentanyl, acrylfentanyl, butyryl fentanyl, valeryl fentanyl, cyclopropyl fentanyl, methoxyacetyl fentanyl, alpha-methyl thiofentanyl, *o*-methyl furanyl fentanyl and *p*-fluoro isobutyryl fentanyl were purchased from Cayman Chemicals. *cis*-3-Methyl fentanyl, *p*-methoxy furanyl fentanyl, *p*-fluoro fentanyl and *p*-methoxy butyryl fentanyl were provided by the United States Drug Enforcement Administration's Southwest Laboratory. Fentanyl and its analogs were formulated as hydrochloride (HCl) salts. Heroin HCl, morphine sulfate, codeine phosphate, and lorazepam were purchased from Cayman Chemicals. Noscapine HCl was purchased from Tokyo Chemical Industry. Papaverine HCl was purchased from Acros Organic. Lidocaine HCl was purchased from Alfa Aesar. Cocaine HCl, (+)-pseudoephedrine HCl, (+)-methamphetamine HCl, chlorpromazine HCl, procaine HCl, quinine sulfate, acetaminophen, benzocaine, diphenhydramine HCl, mannitol, lactose and caffeine were purchased from Sigma Aldrich. SYBR Gold was purchased from Invitrogen. Formamide was purchased from Thermo Fisher Scientific. All other reagents were purchased from Sigma Aldrich unless otherwise noted. Streptavidin-modified agarose beads, ExoSAP-IT Express PCR Purification Kit and Nunc 384-well black plate were purchased from Thermo Fisher Scientific. 800 μl micro-gravity columns were purchased from Bio-Rad. GoTaq Hot Start Colorless Master Mix was purchased from Promega. 3 kDa cut-off spin filters were purchased from Millipore. Unmodified oligonucleotides were purchased from Integrated DNA Technologies with standard desalting purification. Fluorophore- or quencher-modified DNA and the DNA library were purchased from Integrated DNA Technologies with HPLC purification. All oligonucleotides were dissolved in PCR-quality water and their concentrations were measured using a NanoDrop 2000 spectrophotometer. Thiolated methylene blue-modified aptamers were purchased from LGC Biosearch Technologies with dual HPLC purification and dissolved in 1× TE Buffer (10 mM Tris–HCl with 1 mM EDTA, pH 8.0). The DNA sequences are shown in the Supplementary Information (Supplementary Table S1). Deionized water with conductivity of 18.2 MΩ × cm was obtained from a Milli-Q EQ 7000 ultrapure water filtration system.

### SELEX procedure

The isolation of aptamers for fentanyl, acetyl fentanyl, and furanyl fentanyl was carried out following a library-immobilized SELEX protocol. The selection buffer included 10 mM Tris–HCl (pH 7.4), 20 mM NaCl, 0.5 mM MgCl_2_ and 1% (v/v) methanol. For SELEX, the initial single-stranded DNA library pool consisted of ∼6 × 10^14^ (1 nmol) oligonucleotides. Each oligonucleotide in the library contained a randomized 30-nt loop flanked by 8-nt stem-forming constant regions and two PCR primer-binding sites. The SELEX process, including library immobilization, washing, target elution, library amplification, and single-strand generation, was performed using a previously reported protocol. (28) Selection strategies and conditions for each SELEX experiment can be found in Supplementary Tables S2–S4.

### High-throughput sequencing (HTS)

HTS for the round 9 and 11 fentanyl pools, round 8 and 10 acetyl fentanyl pools, and round 7 and 10 furanyl fentanyl pools was performed at FIU’s DNA Core Facility with an Ion Personal Genome Machine System with an Ion 318 v2 chip (Thermo Fisher Scientific). To prepare samples for sequencing, the library pool (final concentration: 10 nM) was mixed with GoTaq Hot Start Colorless Master Mix, forward primer (final concentration: 1 μM) and reverse primer (final concentration: 1 μM) and diluted with PCR-quality water to a final volume of 50 μl. Nine cycles of PCR were performed using the following conditions: 2 min at 95°C; 9 cycles of 95°C for 15 s, 58°C for 30 s and 72°C for 45 s; and finally 5 min at 72°C. 40 μl of PCR product was added into 16 μl of ExoSAP-IT reagent in an ice bath. The mixture was then incubated at 37°C for 15 min to degrade remaining primers and dNTPs, followed by incubation at 80°C for 15 min to inactivate the ExoSAP-IT reagent. Upon obtaining the sequencing data, the primer sequences were trimmed by cutadapt, (29) and the abundance of sequences from each pool and enrichment between rounds were calculated using FASTAptamer. (30)

### Aptamer digestion experiments

Screening of aptamer candidates was performed using an exonuclease digestion assay as recently reported. (21) Briefly, 1 μl of aptamer (final concentration 1 μM) was added to 5 μl Tris–HCl solution (final concentration 10 mM, pH 7.4) and heated to 95°C for 10 min and immediately cooled on ice, after which the solution was diluted to a volume of 40 μl with salts, methanol, and BSA (final concentration: 20 mM NaCl, 0.5 mM MgCl_2_, 1% (v/v) MeOH, 0.1 mg/ml BSA). Next, 5 μl of fentanyl, fentanyl analog, or interferent solution (final concentration varied depending on experiment) was added to the reaction mixture and incubated in a thermal cycler (C1000 touch, Bio-Rad) at 25°C for 60 min. Finally, 5 μl of exonuclease mixture (final concentrations: 0.025 U/μl Exo III and 0.05 U/μl Exo I) was added to each reaction. To monitor the digestion progress, 5 μl of the samples were collected at various time-points and added into the wells of a Nunc 384-well black plate containing 25 μl of quench solution (final concentrations: 10 mM Tris–HCl (pH 7.4), 12.5% formamide, 10 mM EDTA, 1 × SYBR Gold). The fluorescence intensity of SYBR gold (λ_ex_/λ_em_ = 495/537 nm) was recorded using a Tecan Infinite M1000 PRO microplate reader and plotted as a function of time. The area under the curve (AUC) of aptamer fluorescence time-course plots was calculated in the absence and presence of target. Resistance value (*R*_value_), a metric of target-binding induced enzyme inhibition, was calculated by (AUC_target_/AUC_blank_) – 1. All experiments were performed at least twice.

### Isothermal titration calorimetry (ITC) experiments

All ITC experiments were performed in selection buffer with a MicroCal ITC200 instrument (Malvern) at 23°C. For each experiment, 300 μl of parent aptamer or major digestion products (Supplementary Table S5) (final concentrations varying depending on experiments) in Tris–HCl buffer (final concentration 10 mM, pH 7.4, without salts) was heated at 95°C for 10 min and immediately cooled on ice. Salts and methanol were subsequently added to match selection buffer conditions before loading into the sample cell. The syringe was loaded with fentanyl, acetyl fentanyl, furanyl fentanyl, quinine, or chlorpromazine in selection buffer. Concentrations used are listed in Supplementary Tables S6 and S7. Typically, each titration consisted of an initial purge injection of 0.4 μl and 19 successive injections of 2 μl with a spacing of 180 sec between each injection. Injection volume and spacing was changed as necessary for certain sequences (F2, F12, F16, F27, F2–38, F12–38 and F16–43). For some aptamers (F5–40, F22–42 and F2–38), an additional 20 injections were performed in the same manner if saturation was not observed. The raw data was first corrected for the dilution heat of the ligand and then analyzed with the MicroCal analysis kit integrated into Origin 7 software and fitted with a single-site binding model.

### Strand-displacement fluorescence assay

#### Affinity testing of F27-FAM and F27–42-FAM

This assay was performed using a previously reported protocol. (16,17) The *K*_D_ between target and aptamer is equal to the ratio of *K*_D1_ (binding between complementary DNA (cDNA) and aptamer) and *K*_D2_ (affinity of target for aptamer-cDNA complex). To determine *K*_D1_, fluorescein-modified F27 (F27-FAM) was hybridized with a 15-nt dabcyl-modified cDNA (cDNA-Dab), and fluorescein-modified F27 digestion product (F27–42-FAM) was hybridized with a mutated 15-nt dabcyl-modified cDNA containing a single G–T mismatch (cDNA-GT-Dab). Specifically, 72 μl of F27-FAM dissolved in 1× selection buffer (final concentration: 50 nM) was mixed with 8 μl of various concentrations of cDNA-Dab (final concentrations: 0, 7, 15, 31, 62, 125, 250, 500 or 1000 nM) in 1× selection buffer. The mixture was heated to 90°C for 10 min, and then cooled gradually to room temperature over 20 min to promote annealing. Afterwards, 70 μl of each solution was loaded into the wells of a Nunc 384-well black plate and the fluorescence intensity was immediately recorded using a Tecan microplate reader (λ_ex_/λ_em_ = 490/520 nm). The same procedure was performed for F27–42-FAM, except cDNA-GT-Dab was used (final concentrations: 0, 16, 31, 62.5, 125, 250, 500, 1000, 2000 or 4000 nM). *K*_D1_ was determined by fitting the curve using a Langmuir binding isotherm and calculating the free cDNA at 50% quenching efficiency, obtaining values of 15.2 ± 1 and 96.2 ± 15 nM for F27-FAM and F27–42-FAM, respectively. To determine *K*_D2_, a 72 μl solution of aptamer-cDNA complex (final concentrations: 50 nM F27-FAM and 125 nM cDNA-Dab) dissolved in 1× selection buffer was heated to 90°C for 10 min and cooled to room temperature over 20 min to promote perfect hybridization between both strands. Then 8 μl of acetyl fentanyl or furanyl fentanyl at various concentrations in 1× selection buffer (final concentrations: 0, 5, 10, 20, 39, 78, 156, 312.5, 625, 1250, 2500 and 5000 nM) was added to the aptamer/cDNA complex. The mixture was incubated at room temperature for 1 h to allow the system to reach equilibrium, after which 70 μl of each solution was loaded into the wells of a Nunc 384-well black plate with the fluorescence intensity (λ_ex_/λ_em_ = 490/520 nm) immediately recorded. *K*_D2_ was calculated by fitting the fluorescence recovery using a Langmuir binding isotherm, and dividing the free cDNA by the free target concentration at the half-saturation point of the cDNA displacement curve. For F27-FAM, *K*_D2_ values of 1.23 and 2.02 were obtained for acetyl fentanyl and furanyl fentanyl, respectively. Based on *K*_D1_ and *K*_D2_, F27 had a *K*_D_ of 12.4 ± 1 and 7.5 ± 0.4 nM for acetyl fentanyl and furanyl fentanyl, respectively.

#### Determination of aptamer specificity and cross-reactivity

First, the concentration of cDNA-Dab necessary to achieve ∼75% quenching efficiency was determined. 72 μl of F27-FAM or F27–42-FAM (final concentrations: 50 nM) dissolved in 1× selection buffer was mixed with 8 μl of various concentrations of cDNA-Dab in 1× selection buffer (final concentrations: 0, 7, 15, 31, 62, 125, 250, 500 or 1000 nM). The mixture was heated to 90°C for 10 min, and slowly cooled down to room temperature over 20 min to promote annealing of both strands. Afterwards, 70 μl of each solution was loaded into the wells of a Nunc 384-well black plate and the fluorescence intensity was immediately recorded using a Tecan microplate reader (λ_ex_/λ_em_ = 490/520 nm). Optimized aptamer/cDNA ratios (50 nM F27-FAM or F27–42-FAM/250 nM cDNA-Dab) were used to determine each aptamer's specificity against the 19 counter-targets employed during selection or cross-reactivity against fentanyl and its 14 analogs. Specifically, 72 μl of aptamer/cDNA mixture prepared in 1.1× selection buffer was heated and then cooled as described previously to promote annealing. This mixture was then added to 8 μl of selection target or analog (final concentration: 10 μM) or interferent (final concentration: 100 μM) and incubated for 1 h to allow the system to reach equilibrium. To enhance solubility, target and interferent molecules were prepared in deionized water containing 10% methanol. 70 μl of each sample was then loaded into the wells of a Nunc 384-well black plate and the fluorescence intensity (λ_ex_/λ_em_ = 490/520 nm) was immediately recorded. For F27-FAM and F27–42-FAM, cross-reactivity was calculated based on the signal produced by furanyl fentanyl. All experiments were performed at least twice.

### Fabrication of electrochemical aptamer-based (E-AB) sensors

Aptamer-modified gold electrodes were prepared using a target-assisted immobilization strategy. (31) Polishing and cleaning of the electrode was performed using a previously reported protocol. (32) Methylene blue-modified thiolated aptamers (F13–32-MB or F27–38-MB) were mixed with 100 mM Tris(2-carboxyethyl)phosphine hydrochloride in deionized water for 2 h to reduce their disulfide bonds. Freshly-reduced F27–38-MB or F13–32-MB were further diluted to various concentrations (final concentrations: 10, 25 or 50 nM F27–38-MB or 50, 100 or 150 nM F13–32-MB) in selection buffer containing 50 μM fentanyl or acetyl fentanyl, respectively. Cleaned electrodes were incubated in the aptamer solution for 13 h at room temperature in the dark. After aptamer immobilization, the electrode was rinsed with deionized water and then backfilled with 1 mM 6-mercapto-1-hexanol prepared in 1× selection buffer containing 50 μM fentanyl or acetyl fentanyl for F27–38-MB or F13–32-MB, respectively, for 2 h. Finally, the electrodes were rinsed with deionized water and stored in 10 mM Tris–HCl (pH 7.4) for 1 h prior to measurements.

### Electrochemical measurements and optimization of E-AB sensor performance

All electrochemical measurements were performed using a CHI760D electrochemical workstation with a three-electrode system containing an Ag/AgCl reference electrode (3M KCl) (CHI), a platinum wire counter electrode (CHI), and an aptamer-modified gold working electrode. To optimize surface coverage, E-AB sensors were constructed using 10, 25 or 50 nM F27–38-MB or 50, 100 or 150 nM F13–32-MB. Peak currents produced by the target at various concentrations were obtained with a square-wave voltammetry (SWV) measurement frequency of 200 Hz. Signal gain was calculated using the equation: (*I* – *I*_0_)/*I*_0_ × 100%, where *I* and *I*_0_ are the peak current in the presence and absence of target, respectively. The best sensing performance was achieved with electrodes modified with 10 nM F27–38-MB or 100 nM F13–32-MB, corresponding to a surface coverage of 2.17 ± 0.1 and 3.33 ± 0.4 pmol/cm^2^, respectively, based on the method reported by Tarlov *et al.* (33) Next, we optimized the frequency of SWV measurements in the range of 50–400 Hz by measuring the signal gain with or without target. The optimal frequencies were 400 Hz and 200 Hz for E-AB sensors constructed with F27–38-MB and F13–32-MB, respectively. All data represent the average of measurements taken with three independent working electrodes.

### Cross-reactivity and binary-mixture measurements using E-AB sensors constructed with F13–32-MB

E-AB sensors were fabricated using 100 nM F13–32-MB. Each electrode was placed in 1 ml selection buffer with or without fentanyl or one of its analogs (final concentration: 5 μM). After 1 minute incubation, the peak SWV current was recorded three times and the average current was used to calculate signal gain. Sensor cross-reactivity was calculated relative to the signal gain produced by 5 μM fentanyl. For detection of fentanyl in binary mixtures, a similar detection procedure was performed except the electrodes were placed in 1 ml selection buffer containing either 5 μM fentanyl or 5 μM fentanyl mixed with 500 μM cocaine, lactose, mannitol, quinine, lidocaine, heroin, benzocaine, (+)-methamphetamine, diphenhydramine, (+)-pseudoephedrine, acetaminophen, codeine, chlorpromazine, morphine, caffeine, or procaine, or 200 μM papaverine, noscapine, or lorazepam. Cross-reactivity was calculated relative to the signal gain produced by 5 μM fentanyl. All data represent the average of measurements taken with three independent working electrodes.

### Detection of fentanyl in 50% saliva using E-AB sensors constructed with F27–38-MB

To perform detection of fentanyl in saliva, we first prepared a human saliva pool based on a published protocol. (34) Drug-spiked 50% saliva samples were prepared by mixing 500 μl of 2× selection buffer with or without fentanyl (for background determination) with 500 μl of human saliva. Prior to detection, sensors constructed with 10 nM F27–38-MB were first incubated in a 6 M guanidinium HCl solution for 10 min to unfold the aptamers and release any remaining fentanyl from the target-assisted immobilization step. The treated electrodes were then incubated in fentanyl-spiked 50% saliva samples for 1 min, and the peak SWV current was recorded three times. Signal gain was calculated using the average peak current. All data represent the average of measurements taken with three independent working electrodes.

## RESULTS AND DISCUSSION

### Isolation of fentanyl aptamers using SELEX

**Figure 1.**
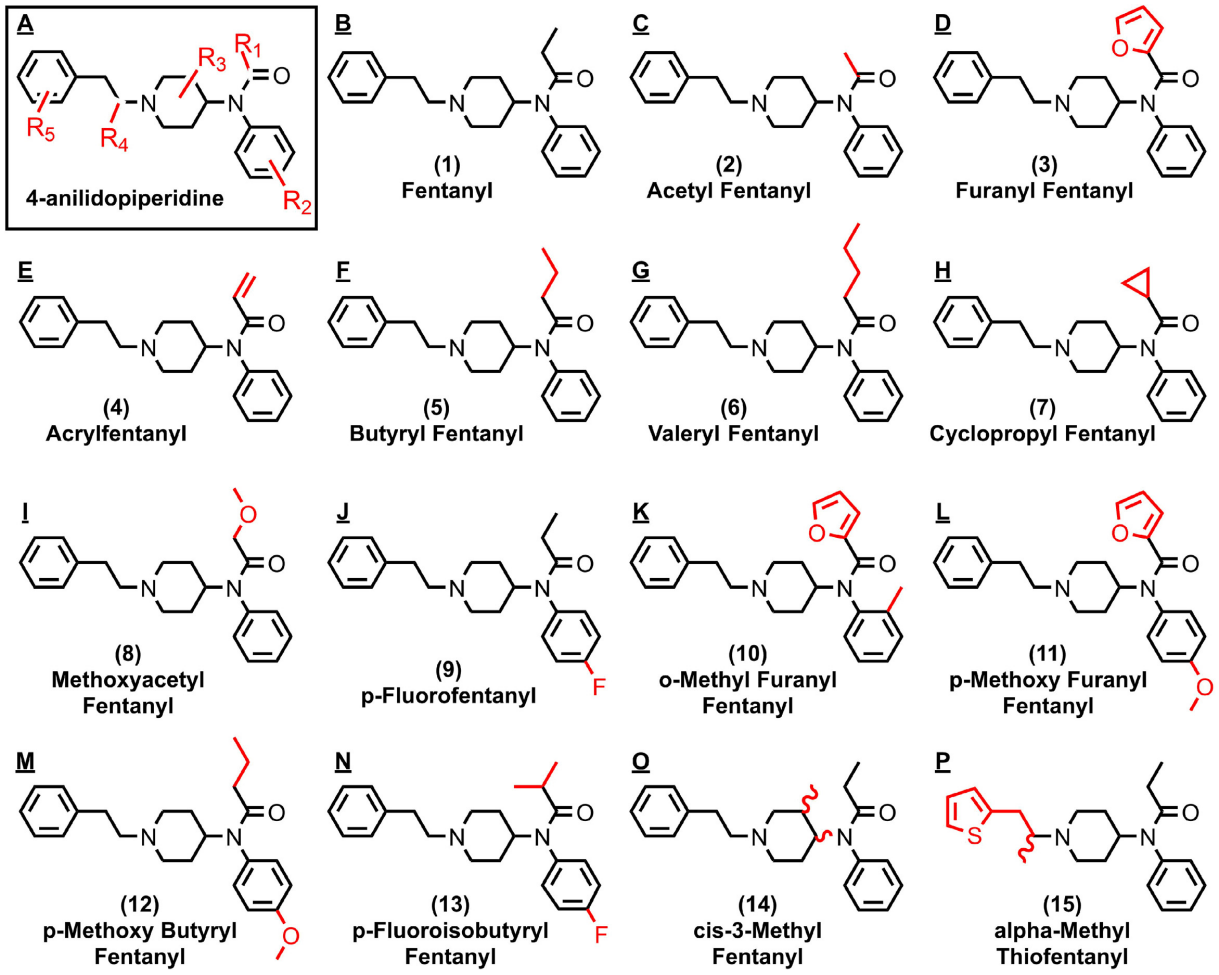
The chemical structures of fentanyl and its analogs, with substituent sites marked in red.

The fentanyl analogs are a family of novel psychoactive substances that share the same 4-anilidopiperidine core structure with fentanyl, but with various chemical modifications at five different substituent sites (Figure 1A, R1–R5) (35). For the present work, we chose fentanyl, acetyl fentanyl, and furanyl fentanyl (Figure 1B–D) as selection targets because these three are most the commonly encountered members in the family. We performed three independent library-immobilized SELEX experiments against these targets with a 73-nucleotide (nt) stem-loop structured DNA library (36) (Supplementary Table S1). Each oligonucleotide in the library contains a 30-nt random region that serves as the putative binding domain, an 8-base-pair stem, and two primer-binding sites at both termini for PCR amplification. Details on the selection conditions and strategies used for the three SELEX experiments are shown in Supplementary Tables S2–S4.

We started from a target concentration of 500 μM in round 1, and this was gradually reduced to ≤50 μM in the final round to enrich high-affinity aptamers. We also performed counter-SELEX to remove sequences that bind adulterants and cutting agents including procaine, lidocaine, quinine, acetaminophen, (+)-pseudoephedrine, benzocaine, diphenhydramine, chlorpromazine, lactose, mannitol, caffeine, noscapine and papaverine as well as the controlled substances cocaine, (+)-methamphetamine, heroin, codeine, morphine, and lorazepam at concentrations ranging from 100–500 μM as SELEX progressed. We used a previously reported gel-elution assay (28) to monitor the selection progress, and observed that SELEX screening for fentanyl was completed within 11 rounds (Supplementary Figure S1A). The final pool bound to this target with a *K*_D_ of 17 μM (Supplementary Figure S1B), and exhibited equivalent cross-reactivity to 25 μM acetyl fentanyl and 74% cross-reactivity to 25 μM furanyl fentanyl (Supplementary Figure S1C). The pool was also specific against other molecules, exhibiting <5% cross-reactivity to 16 counter-targets at 250 μM, although we observed cross-reactivities ≥ 12% for lorazepam, noscapine, and papaverine. Based on this result, we performed counter-SELEX in earlier rounds for the acetyl fentanyl and furanyl fentanyl selection process to remove binders against those three interferents. For both targets, selection was complete within 10 rounds (Supplementary Figures S2A and S3A), with final pool binding affinities of 15 μM for acetyl fentanyl (Supplementary Figure S2B) and 9 μM for furanyl fentanyl (Supplementary Figure S3B), with each pool retaining high cross-reactivity to the other selection targets (Supplementary Figures S2C and S3C). Both pools demonstrated reduced cross-reactivity to lorazepam, noscapine, and papaverine relative to the fentanyl pool, but binding to these interferents was not completely removed. This may be because these ligands can bind G-rich and duplexed DNA sequences indiscriminately. (37,38)

### High-throughput sequencing of enriched pools

We sequenced the round 9 and 11 fentanyl pools, round 8 and 10 acetyl fentanyl pools, and round 7 and 10 furanyl fentanyl pools, obtaining 435632, 521281, 381252, 492320, 410943 and 1425919 reads, respectively. The PCR primer sequences were removed using the cutadapt software (29), and the abundance and enrichment-fold of sequences from each of the pools were calculated using the FASTAptamer software. (30)

**Figure 2.**
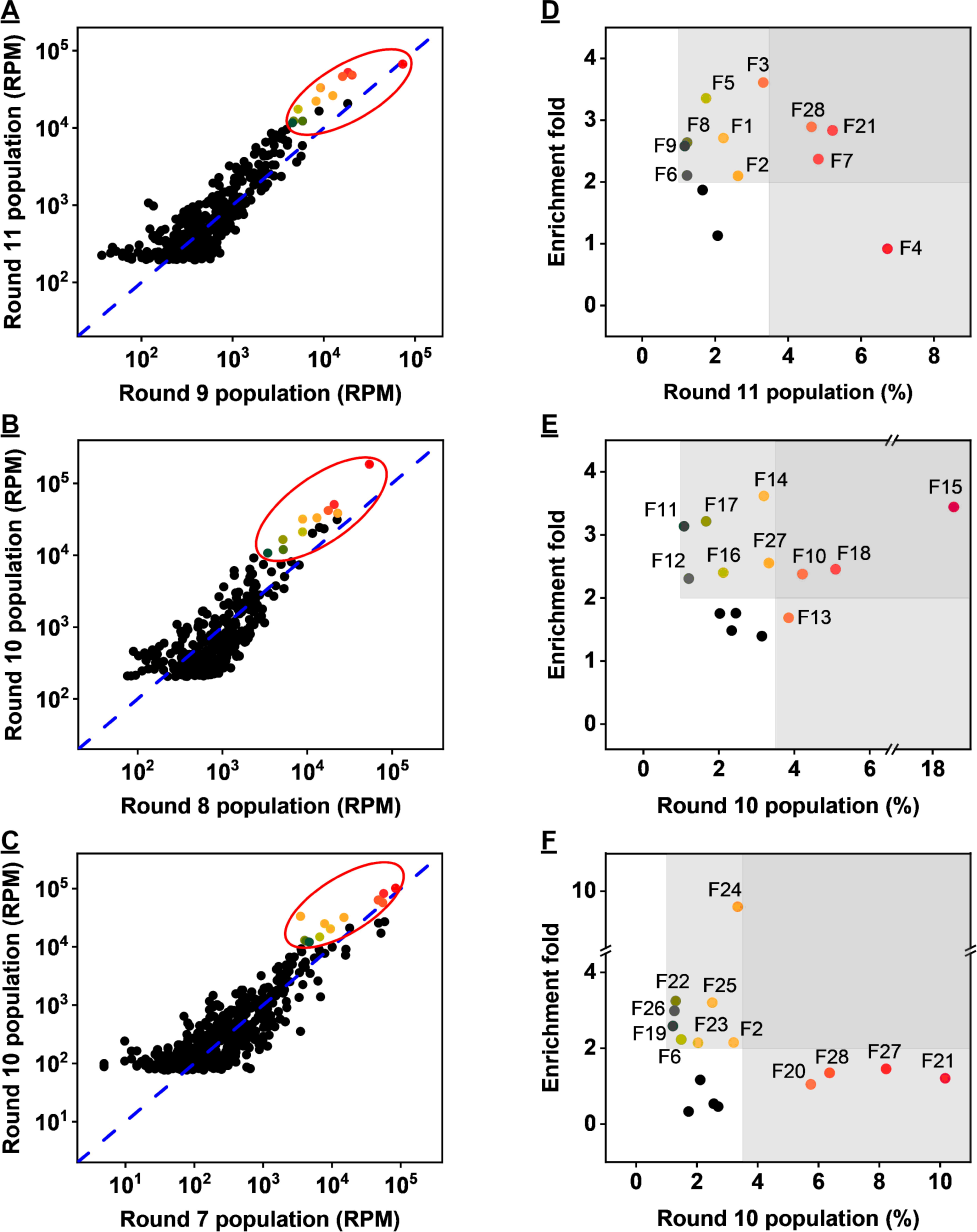
High throughput sequencing of enriched pools. (A–C) Analysis of sequence population growth between (**A**) round 9 and 11 fentanyl pools, (**B**) round 8 and 10 acetyl fentanyl pools and (**C**) round 7 and 10 furanyl fentanyl pools. (D–F) Enrichment-fold of sequences present at >1% of the (**D**) round 11 fentanyl pool, (**E**) round 10 acetyl fentanyl pool and (**F**) round 10 furanyl fentanyl pool. Sequences selected for binding affinity characterization are circled (left) and colored (right), based on the enrichment fold and abundance thresholds defined by the highlighted gray areas. RPM represents reads-per-million.

To identify potential aptamer candidates, we first plotted the population of sequences in both selection rounds for each SELEX experiment against each other in a two-dimensional coordinate system. In Figure 2A–C, each black dot represents a unique sequence, and its position relative to the dashed blue line represents its enrichment-fold. Sequences on the line were equally abundant in both rounds, while sequences above or below the line respectively underwent positive or negative enrichment in between the two rounds. In all three selection experiments, we observed several sequences demonstrating positive or negative enrichment, which indicated that the pools experienced significant evolution. However, only a few sequences were both highly abundant and positively enriched (circled in Figure 2A–C). We plotted the abundance of the sequences in the final round against their enrichment-fold and chose candidates for which the abundance of the sequence in the final round was ≥3.5%, or which had an enrichment-fold >2 and an abundance ≥1% in the final round (colored, labeled dots in Figure 2D–F). Thirty-three sequences met these criteria, and five sequences appeared in more than one pool enriched with different targets. In particular, aptamers F2, F6, F21 and F28 appeared in both the round 11 fentanyl and round 10 furanyl fentanyl pools, and F27 appeared in both the round 10 acetyl fentanyl and round 10 furanyl fentanyl pools. In the end, 28 candidate sequences were chosen for further binding affinity characterization (Supplementary Table S1).

### Rapid screening of target-binding affinity for 28 aptamer candidates

**Figure 3.**
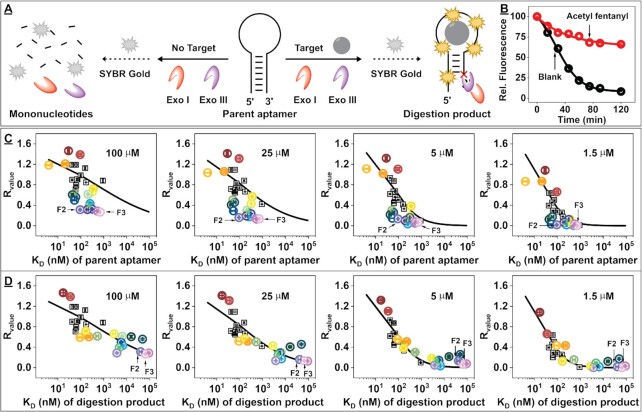
Correlation between target-binding-induced exonuclease inhibition and aptamer binding affinity. (**A**) Schematic of our label-free exonuclease digestion assay for discriminating non-bound (left) from ligand-bound (right) aptamers. (**B**) Digestion time-course of aptamer candidate F27 in the absence and presence of 100 μM acetyl fentanyl. (**C**) Relationship between parent aptamer *K*_D_ and the R_value_ of each aptamer at 100, 25, 5 or 1.5 μM concentrations of their respective selection target (s). Colored points indicate aptamers that do not appear to correlate with the Langmuir fit curve (black line), which was fitted using the dataset from panel D. (**D**) The same plot, but for the major digestion products of the outliers from C (colored) or the parent aptamers that exhibited robust fitting in C (black). The binding affinities of parent aptamers and major digestion products were determined using ITC.

We used our exonuclease digestion assay (21,22) to characterize the target-binding affinity of these newly isolated aptamer candidates. This assay utilizes Exo III, a 3’-to-5’ double-strand DNA exonuclease, and Exo I, a 3’-to-5’ single-strand DNA exonuclease, to differentiate between ligand-bound and unbound DNA aptamers. Exo III and Exo I progressively digest unbound aptamers into mononucleotides in a sequence-independent manner (Figure 3A, left), but the digestion of ligand-bound aptamers is inhibited (Figure 3A right). The digestion process can be monitored using the fluorescent DNA-binding dye SYBR Gold (39). Aptamers that bind tightly to the target remain relatively intact over the course of the whole digestion period and emit high levels of fluorescence relative to unbound or weakly-bound aptamers, which are degraded and undergo a rapid decrease in fluorescence (Figure 3B). We digested the 28 aptamer candidates using a mixture of Exo III and Exo I in the absence and presence of 100 μM of their respective selection target(s) (a total of 33 aptamer–ligand pairs). We collected aliquots of the reaction mixtures at various time points, quenched the reaction with EDTA and formamide, and quantified the digestion products using SYBR Gold. The fluorescence intensity of each aptamer candidate was plotted against reaction time to monitor the digestion progress (Supplementary Figures S4–S6). The digestion kinetics of the aptamers can be assessed using several parameters, including first-order reaction rate, reaction half-life, and area under the curve (AUC) of the digestion time plot. (40) Since reaction rate and half-life are prone to large fitting errors due to the requirement for exponential fitting, we analyzed the digestion kinetics using the AUC parameter. To eliminate the influence of aptamer sequence and structure on digestion kinetics, we created resistance value (*R*_value_) as a metric for evaluating aptamer–ligand binding strength, where *R*_value_ = (AUC_target_/AUC_blank_) – 1. A greater *R*_value_ indicates strong aptamer–ligand binding, whereas a *R*_value_ of 0 indicates that the aptamer and ligand have no affinity. We found that eleven aptamers (F4, F6, F9, F14, F15, F16, F17, F18, F25, F27 and F28) had *R*_value_ >0.8, nine aptamers (F5, F7, F8, F12, F13, F20, F23, F24 and F26) had *R*_value_ between 0.4 and 0.8, and eight aptamers (F1, F2, F3, F10, F11, F19, F21 and F22) had *R*_value_ below 0.4 (Supplementary Figure S7).

To establish a definitive quantitative relationship between *R*_value_ and equilibrium dissociation constant (*K*_D_), we determined the binding affinity of the 28 aptamer candidates for their respective target(s) using the gold-standard ITC method (41) (Supplementary Figures S8–13; Table S6). Twenty-six of the candidates bound to their targets with nM–μM affinity, while two aptamers (F11 and F19) had very weak binding affinity (*K*_D_ > 25 μM) and were excluded from further analysis. This data reflects the results of the exonuclease digestion assay, which indicated that those same 26 aptamers with moderate to high affinity had a *R*_value_ of 0.15–1.46, while the two weakly-binding aptamers had *R*_value_ <0.15. Aptamer F27 had a *K*_D_ of 4 ± 1 nM and 21 ± 5 nM for furanyl fentanyl and acetyl fentanyl, respectively (Supplementary Figure S13B and C), and we further confirmed this result using the fluorescence strand-displacement assay (16,17), obtaining similar *K*_D_s of 6.5 ± 0.4 nM and 12.4 ± 1 nM for furanyl fentanyl and acetyl fentanyl, respectively, (Supplementary Figure S14). When the *K*_D_ of each aptamer was plotted against the *R*_value_, we noted several aptamers had similar *K*_D_ but vastly differing *R*_value_ (e.g. F2 (to furanyl fentanyl), F6 (to fentanyl), F7, F12, F14, F16, F24, F26 and F28, which exhibited *K*_D_ = 42–94 nM and *R*_value_ = 0.5–1.4) and vice-versa (e.g. F1, F2 (to fentanyl), F3, F10 and F21, which exhibited *K*_D_ = 93–709 nM and *R*_value_ = 0.27–0.32) (Figure 3C). To understand this conflicting information and better discriminate the target affinity of these aptamers, we repeated the exonuclease digestion assay using lower target concentrations (25, 5 or 1.5 μM) (Figure 3C; and Supplementary Figure S15–S17). Although we were now able to distinguish high-affinity binders from weak-binding sequences at lower target concentrations, some high-affinity aptamers displayed uncharacteristically low *R*_value_. For instance, high-affinity aptamers F2 (*K*_D_ = 53 ± 12 nM) and F3 (*K*_D_ = 709 ± 37 nM) were unable to achieve *R*_value_ > 0.4 even at 100 μM target.

In the exonuclease digestion assay, target-bound aptamers are typically truncated up to a point 4–6 nt from their 3’ termini (21,22,42,43), and we hypothesized that the digestion products formed in this assay may have divergent binding affinities from their parent aptamers, which would explain the lack of a clear relationship between *R*_value_ and *K*_D_ in our initial dataset (Figure 3C). To confirm this, we identified the major digestion product of the 16 parent aptamers (F1, F2, F3, F5, F7, F8, F10, F12, F13, F16, F18, F20, F21, F22, F23 and F27) that had seemingly contradictory *R*_value_ and *K*_D_ (Figure 3C, colored) by digesting them with and without their target (a total of 19 aptamer–ligand pairs) and analyzing their products using denatured polyacrylamide gel electrophoresis (Supplementary Figures S18–23). Each digestion product was identified (Supplementary Table S5) using a customized DNA ladder based on the original parent aptamer and then chemically synthesized for subsequent affinity testing using ITC (Supplementary Figures S24–S27, Table S7). We observed considerable variability in the number of base-pairs retained in the initial stem of the parent aptamers—ranging from two to six. As expected, all 16 digestion products consistently displayed differing binding affinities from their parent aptamers. For instance, aptamers F8, F16 and F18, which had higher than expected *R*_values_ in the exonuclease digestion assay, indeed had ∼2-fold improved binding affinity relative to their parent aptamers. Likewise, the digestion products of the other 13 aptamers with lower than expected *R*_value_ had binding affinities that were 7–800-fold weaker than that of their parent aptamer. Collectively, these data indicate that the *K*_D_ of the aptamer's major digestion product largely influences their *R*_value_, and when we plotted the *K*_D_ of these digestion products against *R*_value_ and fitted with a Langmuir equation (Figure 3D), we observed a strong correlation between the two parameters—with *R*^2^ values of 0.875, 0.910, 0.917 and 0.931 for 1.5, 5, 25 and 100 μM target, respectively (Supplementary Figure S28, and Table S8). The datasets established at each target concentration are suited for predicting affinity for aptamers with a specific range of affinities. In particular, the 100 and 25 μM datasets should be used for aptamers with high nM to low micromolar *K*_D_s, while the 5 and 1.5 μM datasets are more appropriate to apply for aptamers with low nM affinity. These results demonstrate that there is a definite quantitative relationship between *R*_value_ and *K*_D_ of the major digestion product of an aptamer.

### High-throughput screening of aptamer specificity

**Figure 4.**
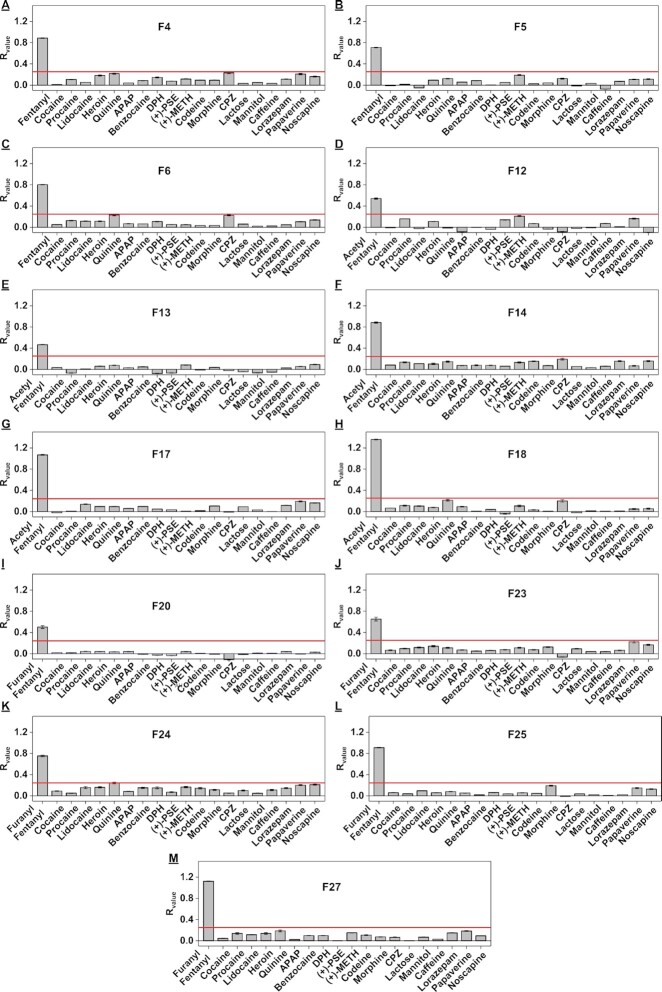
Screening the specificity of twelve aptamer candidates using our exonuclease digestion assay. Plots show *R*_value_ obtained from time-course digestion of (**A**) F4, (**B**) F5, (**C**) F6, (**D**) F12, (**E**) F13, (**F**) F14, (**G**) F17, (**H**) F18, (**I**) F20, (**J**) F23, (**K**) F24, (**L**) F25 and (**M**) F27 in the presence of 100 μM selection target or various interferents. The red line indicates a *R*_value_ of 0.25, which is predicted to be equivalent to a *K*_D_ of ∼100 μM. Acetaminophen (APAP), diphenhydramine (DPH), (+)-pseudoephedrine ( (+)-PSE), (+)-methamphetamine ( (+)-METH), chlorpromazine (CPZ).

The proportion of fentanyl in seized substances is low, typically ranging between 0.1 and 1% by weight. (44) The aptamers used for fentanyl detection must therefore be highly specific in order to discriminate their target from the more abundant interferents. To demonstrate that our exonuclease digestion assay can accurately determine aptamer specificity, we characterized the binding of the 20 aptamers that had a *R*_value_ ≥0.4 (*K*_D_ ∼ 15 μM) against 19 interferents commonly found in drug samples. These included six controlled substances (cocaine, codeine, heroin, lorazepam, morphine and (+)-methamphetamine) and thirteen adulterants and cutting agents (acetaminophen, benzocaine, chlorpromazine, diphenhydramine, lidocaine, noscapine, procaine, papaverine, quinine, caffeine, lactose, mannitol, (+)-pseudoephedrine) (Supplementary Figure S29). We digested each aptamer in the absence or presence of 100 μM interferent (Supplementary Figure S30–31), and used a threshold *R*_value_ of 0.25 (translating to *K*_D_ ∼ 100 μM) to identify specific aptamers. Thirteen aptamers (F4, F5, F6, F12, F13, F14, F17, F18, F20, F23, F24, F25 and F27) had *R*_value_ <0.25 for all 19 interferents (Figure 4), indicating excellent specificity, while the other seven had a *R*_value_ >0.25 for at least one interferent (Supplementary Figure S32). These findings corroborated well with ITC results. For example, using the *R*_value_ obtained for F7-quinine, we predicted a *K*_D_ of ∼30 μM, which closely matched the *K*_D_ of the major digestion product (F7–40) as determined by ITC (*K*_D_ = 62.1 ± 4.9 μM) (Supplementary Figure S33). We omitted the seven low-specificity candidates from subsequent cross-reactivity studies.

Since the affinity data from the exonuclease assay reflects that of the digestion product, we hypothesized that the specificity profile attained from this assay actually represents the binding properties of its major digestion product rather than the parent aptamer. To confirm this, we performed a strand-displacement fluorescence assay using one of the digestion products labeled with FAM (F27–42-FAM) against the abovementioned interferents. We found that the results in this assay were consistent with those obtained from exonuclease digestion assay (Supplementary Figure S34A), demonstrating the reliability of our exonuclease digestion assay for assessing aptamer specificity. To further verify if the major digestion product has a similar specificity with its parent aptamer, we tested the specificity of FAM-modified parent aptamer (F27-FAM) in the strand-displacement fluorescence assay. As expected, we observed that parent aptamer and its major digestion product had an almost identical specificity profile (Supplementary Figure S34B). These results indicated that exonuclease digestion product has similar binding profile with its parent aptamer.

### High-throughput screening of aptamer cross-reactivity to fentanyl analogs

**Figure 5.**
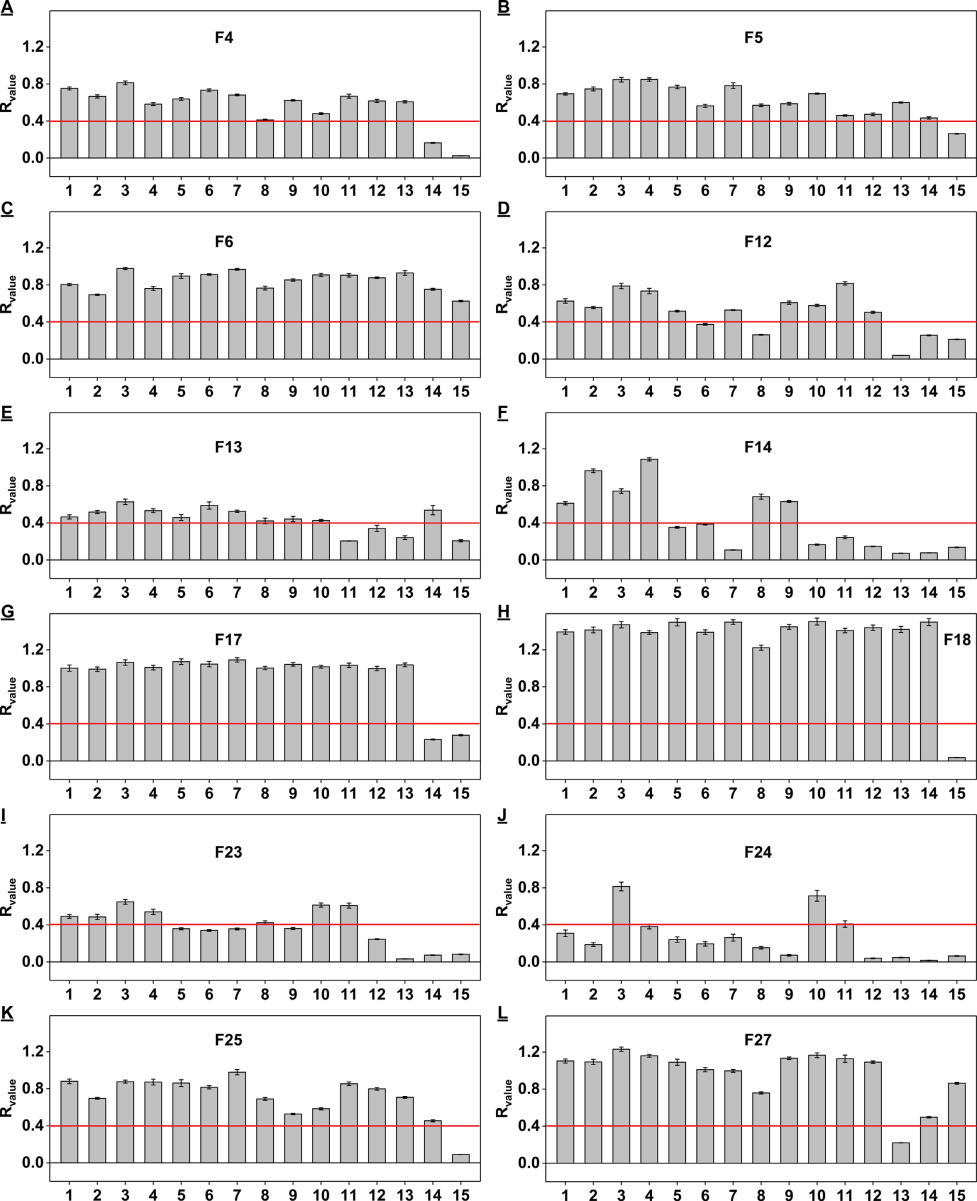
Screening the cross-reactivity of 12 aptamer candidates to fentanyl and its analogs using our exonuclease digestion assay. *R*_value_ from time-course digestion of (**A**) F4, (**B**) F5, (**C**) F6, (**D**) F12, (**E**) F13, (**F**) F14, (**G**) F17, (**H**) F18, (**I**) F23, (**J**) F24, (**K**) F25 and (**L**) F27 in the presence of 100 μM fentanyl (1) or the 14 analogs shown in Figure 1 (drug ID 2–15). The red line indicates *R*_value_ = 0.4, which is predicted to be equivalent to a *K*_D_ of ∼20 μM.

Several structural analogs of fentanyl have been identified, and it is beneficial to develop an aptamer-based assay that can detect as many drugs in this family as possible. We therefore assessed whether our exonuclease digestion assay can determine the cross-reactivity of our twelve aptamer candidates (F4, F5, F6, F12, F13, F14, F17, F18, F23, F24, F25 and F27) to 100 μM fentanyl or 14 of its analogs: acetyl fentanyl, furanyl fentanyl, acrylfentanyl, butyryl fentanyl, valeryl fentanyl, cyclopropyl fentanyl, methoxyacetyl fentanyl, *p*-fluorofentanyl, *o*-methyl furanyl fentanyl, *p*-methoxy furanyl fentanyl, *p*-methoxy butyryl fentanyl, *p*-fluoroisobutyryl fentanyl, *cis*-3-methyl fentanyl, alpha-methyl thiofentanyl (Figure 1, and Supplementary Figure S35). We used *R*_value_ ≥0.4 (*K*_D_ ∼20 μM) as a cutoff to identify aptamers with sufficient binding affinity. Our results indicated that aptamers F4, F5, F12, F13, F17, F18 and F25 were more tolerant of larger moieties at the R_1_ position, such as butyl and valeryl groups, but could not bind to analogs with alterations at R_3_ and/or R_4_ (Figure 5A, B, D, E, G, H, K). F14 cross-reacted to analogs containing relatively small moieties at R_1_ (e.g. fentanyl, acetyl fentanyl, acrylfentanyl), but had less tolerance for alterations at the other substituent sites (Figure 5F). This is perhaps because this aptamer originated from the SELEX experiment using acetyl fentanyl, the analog with the smallest R_1_ substituent (a methyl group). On the other hand, F23 and F24 had high specificity for furanyl fentanyl and other analogs containing the furanyl group at R_1_, as well as moderate affinity to acrylfentanyl, which possesses a similar planar substituent to furanyl fentanyl (Figure 5I, J). This was expected, as these aptamers originated from the furanyl fentanyl pool.

The most cross-reactive aptamer was F6, which bound strongly to all tested analogs with *R*_value_ ≥0.4 (Figure 5C). However, the aptamer did display some cross-reactivity to chlorpromazine and quinine, which would be suboptimal for detection applications. The second most cross-reactive aptamer was F27, which could recognize modifications at each position but could not bind *p*-fluoroisobutyryl fentanyl, which has both R_1_ and R_2_ modifications (Figure 5L). To verify that the cross-reactivity data obtained via the exonuclease digestion assay reflects that of the digestion product, we assessed the cross-reactivity of one of the major digestion products labeled with FAM (F27–42-FAM) to various fentanyl analogs using the strand-displacement fluorescence assay. We observed very similar binding profiles from both assays (Supplementary Figure S36A), indicating that the exonuclease digestion assay can be used to accurately screen for an aptamer's cross-reactivity. We also performed the strand-displacement assay with the parent aptamer F27-FAM against fentanyl and its analogs and found that both the parent and truncated aptamer had very similar binding profiles (Supplementary Figures S36B). These results again indicated that our exonuclease digestion assay can accurately discern aptamer binding profiles.

### Development of E-AB sensors with customized analytical performance

Our exonuclease digestion assay streamlines the aptamer sensor development process because it readily generates structure-switching aptamers (42,43) and simultaneously provides detailed information on aptamer affinity, specificity, and cross-reactivity. Structure-switching functionality – the capability of an aptamer to undergo conformational changes upon target binding – is essential for many folding-based aptamer sensor formats, including electrochemical aptamer-based (E-AB) sensors. (45) Here, we created two E-AB sensors optimized for different applications: 1) highly sensitive and specific detection of fentanyl in biological samples and 2) broad presumptive testing for fentanyl and its analogs in seized substances. Using the data from our exonuclease digestion assay, we determined that the digestion products of F27 (F27–42) and F13 (F13–39) would respectively be well-suited for these two applications. F27–42 is suitable for detecting minute quantities of fentanyl in complex biomatrices because it has the requisite binding affinity to fentanyl, with an *R*_value_ of 0.49 from 5 μM fentanyl (Supplementary Figure S37)—equivalent to a predicted *K*_D_ = 147 nM—and an ITC-measured *K*_D_ of 142 ± 20 nM (Supplementary Figure S38A) and robust specificity, with minimal response to all tested interferents (Supplementary Figure S34). On the other hand, F13–39 is more well-suited for detecting a variety of fentanyl analogs present at ≥0.1% on a weight basis in bulk powders given its high cross-reactivity to the analogs and its sufficient affinity (*R*_value_ = 0.43 for 100 μM fentanyl, predicted *K*_D_ = 12 μM, ITC-measured *K*_D_ = 5.6 ± 0.3 μM) (Figure 5E) (Supplementary Figure S38B).

**Figure 6.**
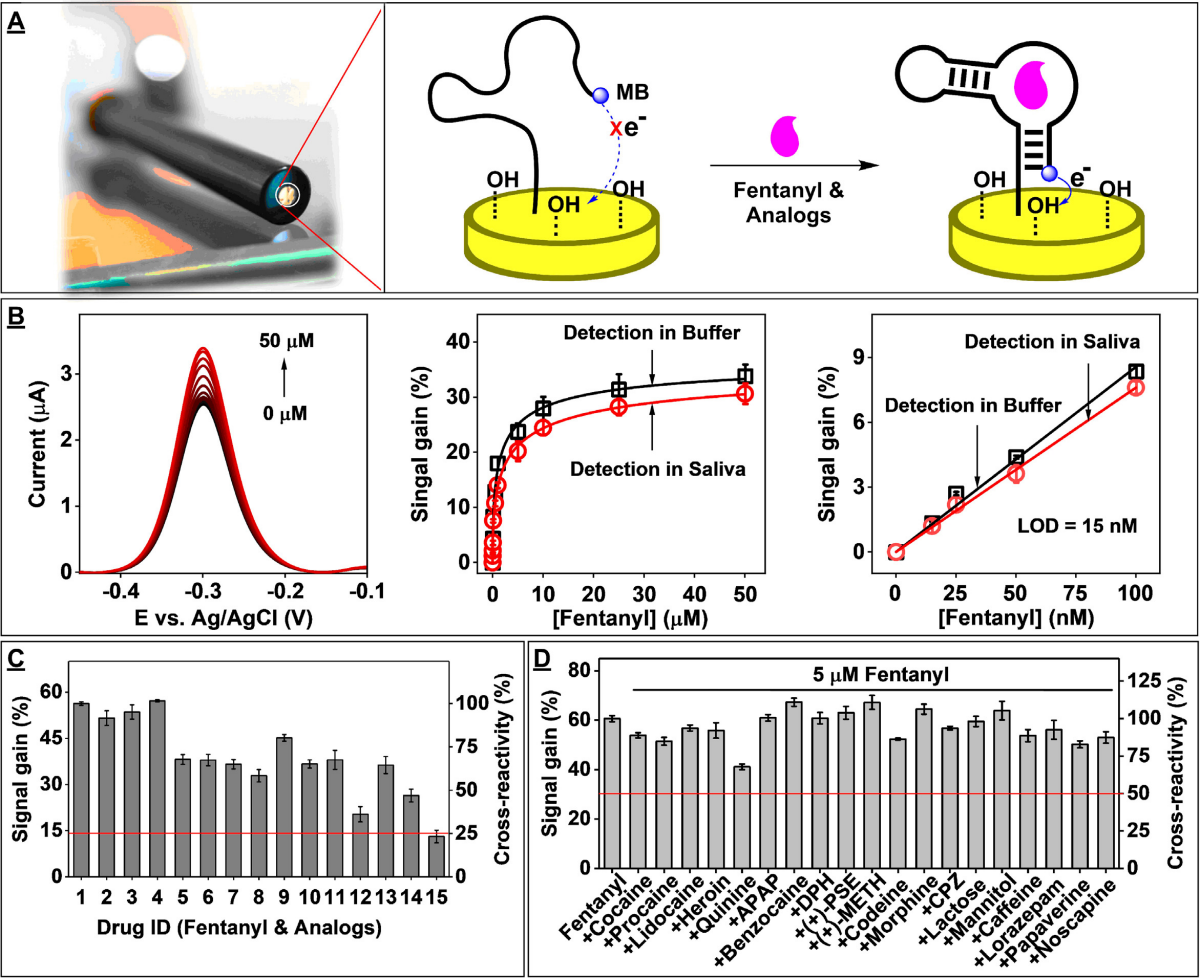
Analytical performance of E-AB sensors for detecting fentanyl or its analogs. (**A**) Working principle of E-AB sensors. (**B**) Raw square-wave voltammetry data (left), calibration curve (middle), and linear range (right) for a F27–38-MB-based E-AB sensor in the presence of 0–50 μM fentanyl in buffer or 50% saliva. Signal gain and cross-reactivity of the F13–32-MB E-AB sensor for (**C**) 5 μM fentanyl and its analogs or (**D**) 5 μM fentanyl in binary mixtures comprising 1:40 or 1:100 molar ratios of fentanyl and various interferents commonly found in seized substances.

We first confirmed that F27–42 and F13–39 had structure-switching functionality using circular dichroism spectroscopy, a gold standard technique for characterizing the conformation of biomolecules. (46) As expected, these aptamers displayed large changes in their circular dichroism spectra upon the addition of fentanyl, indicating a transition from single-stranded to double-stranded B-form DNA for F13–39 (Supplementary Figure S39A), and a transition from single-stranded DNA to either a parallel G-quadruplex or double-stranded, G-C rich, Z-form DNA for F27–42 (Supplementary Figure S39B). (46) Then, to incorporate the aptamers into E-AB sensors, we chemically synthesized blunt variants of these aptamers labeled with a 5’ thiol group and 3’-methylene blue tag (F27–38-MB and F13–32-MB). The aptamers were modified onto gold electrodes using our recently-developed target-assisted aptamer immobilization strategy (31) and we then determined the response of these E-AB sensors to fentanyl. In the absence of target, the electrode-bound aptamers are unfolded, orientating their methylene blue redox tag far from the electrode surface and resulting in a small background current when the electrode is interrogated using voltammetry. When challenged with the target, aptamer-target binding triggers a conformational change that brings the methylene blue tag closer to the electrode surface, thus increasing the redox current in a target-concentration dependent manner (Figure 6A).

At optimal aptamer surface density (Supplementary Figure S40A) and measurement frequency (Supplementary Figure S40B), the F27–38-MB E-AB sensor was able to detect fentanyl at concentrations as low as 15 nM in 50% human saliva (Figure 6B). These results confirmed that this E-AB sensor is potentially capable of assessing recent fentanyl use in saliva, which is typically associated with a concentration ranging from 4 to 16000 nM. (47–49) Meanwhile, under optimized aptamer surface density and sensing parameters (Supplementary Figure S41), the E-AB sensor fabricated with F13–32-MB was able to respond to fentanyl and 14 fentanyl analogs at a 5 μM concentration (∼1.7 μg/ml fentanyl) with ≥ 25% cross-reactivity (Figure 6C) (Supplementary Figure S42) and was capable of detecting fentanyl in binary mixtures containing a 1:100 molar ratio of fentanyl and interferents (Figure 6D) (Supplementary, Figure S43). Based on the specificity profile of this aptamer and the specificity of its corresponding E-AB sensor (Supplementary Figure S44), setting a cut-off of 25% cross-reactivity enables detection of all analogs at 5 μM without false positives to a majority of interferents at 100-fold greater concentration. This however excludes the interferents methamphetamine, procaine, and papaverine, as they produced cross-reactivities in the range of 30–45%. Clearly, this sensor is suitable for sensitive and specific detection of fentanyl family members in seized substances.

## DISCUSSION

Biosensor development is currently hindered by the lack of characterization methods that can rapidly and accurately identify high quality bioreceptors with suitable binding properties for a given sensing application. We have now addressed this issue for DNA aptamers by exploiting a recently-developed exonuclease-based assay that has been shown to provide qualitative insights into aptamer–ligand binding. In the present work, we have proven that our exonuclease assay can also provide quantitative assessments of aptamer–ligand affinity and specificity. We have demonstrated this by isolating a new set of aptamers that bind fentanyl and/or its analogs, and then determining their affinity, specificity, and cross-reactivity using the exonuclease digestion assay across a total of 655 aptamer–ligand pairs. We further validated these results using gold-standard methodologies including ITC and a strand-displacement fluorescence assay. The data indicated that there is a statistically significant inverse correlation between the resistance of a given aptamer–ligand pair to exonuclease digestion (here defined as *R*_value_) and the *K*_D_ of the major digestion product from this assay, and that this relationship can be mathematically modeled and predicted.

This assay offers numerous advantages, including the fact that it is label-free, quantitative, simple, and rapid. In addition, the method requires no prior knowledge of the target-binding domain, in contrast to the strand-displacement assay. Given that the fundamental underlying working principle of the assay has already be shown to be generalizable for aptamers of different structures (e.g. three-way-junctions, stem-loops, G-quadruplexes, triple helixes) and ligands with divergent physiochemical properties, (21,42,43,50–51) we believe our findings regarding the quantitative relationship between aptamer affinity and exonuclease resistance should hold broadly true as well. In fact, we are now routinely employing the exonuclease digestion assay to screen myriads of aptamer candidates we have recently isolated in terms of their binding affinity and specificity, some examples of which are shown in Supplementary Figure S45. These factors together make our assay highly amenable for high-throughput aptamer characterization. Based on the detailed binding profiles of the aptamers ascertained here, we were able to precisely identify structure-switching aptamers suitable for two distinct sensing applications associated with fentanyl: sensitive detection of minute quantities of fentanyl in biosamples and broad presumptive screening of fentanyl and its analogs in seized substances such as bulk powders. We successfully developed a pair of E-AB sensors based on these aptamers that exhibit robust detection capabilities relative to current assays for fentanyl detection, (26,27,52,53) and could play an important role in combatting the opioid epidemic.

We have obtained several new important insights in this study. First, until recently, we were only able to observe a qualitative relationship between aptamer resistance to exonuclease digestion in the presence of target. Here, we have directly correlated digestion kinetics (via *R*_value_) with aptamer target-binding affinity, establishing that there is indeed a quantitative relationship between these two metrics. According to our findings, *R*_value_ can be used to accurately estimate K_D_, something not possible before. Second, we have determined here that digestion resistance is directly related to the target affinity of the truncated aptamer digestion product rather than that of the parent aptamer, as was previously thought. This is an important distinction, as we have demonstrated here that the parent aptamer and truncated digestion product often have differing target-binding affinities. And because the truncated aptamers typically have structure-switching functionality (21,42,43), having detailed information on the binding properties of these truncated aptamers is very useful for assay or sensor development. This means that our exonuclease assay can generate structure-switching aptamers while also quantifying their target-binding affinity, which greatly streamlines sensor development.

We have also demonstrated the necessity of performing high-throughput screens to identify suitable aptamer candidates for analytical use. Often times, data from high-throughput DNA sequencing and bioinformatics analysis can assist by revealing sequences that have the highest relative abundance in the final round or greatest enrichment between rounds. (54) However here we observed that neither the aptamers with the highest population in the final round of SELEX nor those with the greatest enrichment-fold offered the ‘best’ affinities or specificities (Supplementary Figure S46). Indeed, there were even a few aptamers that had relatively high final-round populations (≥1%) and modest enrichment (≥ 2-fold) that demonstrated poor or even no detectable affinity for their target (e.g. F11 and F19). These findings indicate that these commonly applied sequencing metrics are unreliable indicators of aptamer quality, and highlight the need of performing broad characterization of as many potential aptamer candidates as possible—a task which is made feasible with our exonuclease digestion assay. The persistence and enrichment of mediocre aptamers during SELEX can be possibly attributed to bias inherent to the SELEX process that favors the enrichment of non- or weakly-binding sequences, such as low partitioning efficiency when separating binders from non-binders, PCR amplification bias, and biases associated with certain DNA sequencing methods.

Future users of this exonuclease digestion assay should judiciously consider various assay design parameters to ensure the data are correctly interpreted. For instance, it is important to note that *R*_value_ can only be compared for digestions performed at the same concentration of ligand, aptamer, and exonucleases as well as buffer conditions and temperature. Therefore, we recommend using the assay conditions employed herein as a starting point. Digestion should first be performed with a set of candidate aptamers at a fixed concentration (we recommend 0.5 or 1 μM) in the absence of target to determine the time-point required for >90% digestion. It is also important to include control experiments to identify ligands that may non-specifically inhibit enzyme digestion by digesting a sequence that has no binding affinity for each ligand. Next, the aptamers should be digested in the presence of two or three different target concentrations spanning one or two orders of magnitude (we used 1.5–100 μM target). This will ensure that the aptamer is >90% bound with target at at least one target concentration. Selecting appropriate target concentrations is essential for accurate affinity determination. For instance, when determining the affinity of a set of aptamers with *K*_D_ between 10^−8^ and 10^−5^ M, it is recommended that digestion is performed with at least three or four target concentrations within the range of 1–100 μM. This will guarantee that, in at least one concentration, the aptamer will be nearly or completely saturated with ligand, and thus inhibition of aptamer digestion by ligand binding can be observed. Once *R*_value_s are collected, they can be used to predict *K*_D_. Here, the *R*_value_ should be selected based on a target concentration at which dramatic changes in this parameter occur. For instance, an *R*_value_ determined at a relatively low target concentration of 5 μM was suitable to predict *K*_D_ for aptamers with ITC-determined *K*_D_ of 100 nM, as opposed to using an *R*_value_ determined at 100 μM target.

We have established thus far that the rate at which aptamers are digested is affected by whether the aptamer is bound to a ligand. This will influence the shape of the digestion time-course plot, which will in turn influence the area under the curve (AUC) parameter that is used to calculate *R*_value_. Although data thus far indicates a correlation between enzymatic resistance and aptamer-target binding affinity (a thermodynamic parameter), it is also possible that target binding kinetics (i.e. *k*_on_ and *k*_off_) influence the rate at which the aptamer is digested. A detailed investigation will need to be performed in the future to confirm whether this is true.

We envision that assay throughput could be greatly increased in the future through the use of a robotic liquid handling system. This methodology could also be applied to expedite mass mutagenesis screens for thousands of aptamer sequences, thereby enabling the extensive exploration of aptamer structure-function relationships and the identification of novel aptamers with improved binding properties. One could also employ reporting technologies that enable real-time monitoring of the digestion process, which would greatly simplify the assay by removing the need to obtain samples periodically, effectively reducing it to a single mix-and-monitor step.

## DATA AVAILABILITY

The Ion Torrent sequencing data underlying this article are available in SRA at https://www.ncbi.nlm.nih.gov/sra/, and can be accessed with the ID number PRJNA905424.

## Supplementary Material

gkac1210_Supplemental_File
