# Supplementary material for: High-throughput quantitative binding analysis of DNA aptamers using exonucleases
Source: Nucleic Acids Res. 2022 Dec 30;51(4):e19. doi: 10.1093/nar/gkac1210 (PMC9976898; doi:10.1093/nar/gkac1210)
Supplement: gkac1210_Supplemental_File [file gkac1210_supplemental_file.pdf]

Supplementary information for:

High-Throughput Quantitative Binding Analysis of DNA Aptamers using Exonucleases

Juan Canoura[1,2], Obtin Alkhamis[1], Yingzhu Liu[1], Connor Willis[1] and Yi Xiao[1,2*]

[1] Department of Chemistry, North Carolina State University, 2620 Yarbrough Drive, Raleigh, NC, USA, 27607. [2] Department of Chemistry and Biochemistry, Florida International University, 11200 SW 8th Street, Miami, FL, USA, 33199.

*Corresponding author: yxiao34@ncsu.edu

Experimental Section

Polyacrylamide gel electrophoresis (PAGE) analysis of digestion products. Aptamer digestion products were analyzed using denaturing PAGE by collecting 5 µL of samples at various time intervals and mixing with 10 µL formamide loading buffer (75% formamide, 10% glycerol, 0.125% SDS, 10 mM EDTA, and 0.15% (w/v) xylene cyanol). 6 µL of each sample was loaded into the wells of a 15% denaturing PAGE gel. Separation was carried out at 6 V/cm for 30 min followed by 25 V/cm for 4 h in 0.5× TBE running buffer. The gel was stained with 1× SYBR Gold solution for 25 min and imaged using a ChemiDoc MP Image system (Bio-Rad). The major digestion product of each aptamer was determined using a DNA ladder customized for each sequence.

Confirmation of structure-switching functionality of aptamer digestion products using circular dichroism spectroscopy. Circular dichroism experiments were performed at room temperature using a Jasco J-815 circular dichroism spectropolarimeter. F13-39 or F27-42 (1.5 µM final concentration) was prepared in 1× selection buffer, heated to 95 °C for 10 min, and immediately cooled on ice for 5 min. Then deionized water with or without target (final concentration: 10 µM) was added to the solution to a total volume of 300 µL. The solution was transferred to a 1-cm quartz cuvette (Hellma Analytics) and measured using the following parameters: scan range of 210–310 nm, scan speed of 50 nm/min, sensitivity of 5 mdeg, response time of 4 s, bandwidth of 1 nm, and accumulation of 5 scans. Reference spectra of selection buffer were taken with and without target and subtracted from the circular dichroism spectra collected with each aptamer in the absence or presence of its respective target.

Table S1. DNA sequences used in this work.

Sequences ID	Sequence (5'–3')
Random library	CGAGCATAGGCAGAACTTACGAC(N30)GTCGTAAGAGCGAGTCATTC
Bio-cDNA	TTTTTGTCGTAAGTTCTGCCATTTT-/Biotin/
Forward primer	CGAGCATAGGCAGAACTTAC
Biotinylated-reverse primer	/Biotin/-GAATGACTCGCTCTTACGAC
Reverse primer	GAATGACTCGCTCTTACGAC
F1	CTTACGACACGAGGTGTTTGGACTAAGTTCGGTTTCGGGTCGTAAG
F2	CTTACGACGACTGCGTGTGGCCGGTGTGAGGGAGGGTTGTCGTAAG
F3	CTTACGACAGCGGGTGTATGTACTAAGTCCGGTTCGGTGTCGTAAG
F4	CTTACGACACTGGCAGGAGGGTCGGGTGTGGGAACGTGGTCGTAAG
F5	CTTACGACCAGGCCTACGGAAGCAGCGTCAGCGGGGGGGTCGTAAG
F6	CTTACGACTAGTGGAGTAGGGTCGGGTAGTGGGCCTCAGTCGTAAG
F7	CTTACGACCACCATGGGAATCGGGTGGCTTGGAGGTGCGTCGTAAG
F8	CTTACGACGAGCATCGGTTTTTTCGGTGATGTCTGGGAGTCGTAAG
F9	CTTACGACGGAGGTTGGGAAGGAGGGGGAGGCCGGAGAGTCGTAAG
F10	CTTACGACGGCAGGTGTTTGCACTAAGTCCGGTATGTCGTCGTAAG
F11	CTTACGACCGGTGTGCTCGGGGAAGGGGGGCCCTAGGTGTCGTAAG
F12	CTTACGACATCTGCGTGTGGCCGGTGTGAGGGAGGGATGTCGTAAG
F13	CTTACGACCATGGGTGTTTGCACTAAGTCCGGTTCTTGGTCGTAAG
F14	CTTACGACCGGTGTGCTCGGGGAAGGGGGCCCTAGGTGGTCGTAAG
F15	CTTACGACACCGGGATCCAGATGGGTAGTTTGATGTGTGTCGTAAG
F16	CTTACGACCGGCGGAAGGCTGGAGGGGTTGGGGGAGGTGTCGTAAG
F17	CTTACGACCGGTGGGGAGGCCGGAGTTGGGAACGGGGGGTCGTAAG
F18	CTTACGACCGGGATCCTTTGGGACAACCTGGTGGGCATGTCGTAAG
F19	CTTACGACGGGGTACCCGGACAGTGATGTTTGGTGTTCGTCGTAAG
F20	CTTACGACGAAGCAACGGGGTTTCGGAGGGCAGGTGTCGTCGTAAG
F21	CTTACGACCGGACATGTGATCGGGCAGCTGGGAGTCGGGTCGTAAG
F22	CTTACGACGTCGAGGGGTACCCTTTGGCGTTCGTCGAGGTCGTAAG
F23	CTTACGACCAGGCTACGTGGGGGAGGGTGGAAGACGGGTCGTAAG
F24	CTTACGACACAGGGTGTGTTGTGCTCAGTGGTGTATGTGTCGTAAG
F25	CTTACGACAGGGGTACCCGCGTATAACGTGGCGTTCGTGTCGTAAG
F26	CTTACGACGGGGTGGGGGCGGCTTCCCATGGGAGGGGTGTCGTAAG
F27	CTTACGACGAGCGCGTGTGGCCGGCGTGAGGGAGGTGAGTCGTAAG
F28	CTTACGACGGGTGGGGAGGCCCTCTAGTTGGGAACGGTGTCGTAAG
F27-FAM	/FAM/TGGCAGAACTTACGACGAGCGCGTGTGGCCGGCGTGAGGGAGGTGAGTCGTAAG
F27-42-FAM	/FAM/TGGCAGAACTTACGACGAGCGCGTGTGGCCGGCGTGAGGGAGGTGAGTCG
cDNA-Dab	GTCGTAAGTTCTGCC/Dab/
cDNA-GT-Dab	GTCGTAAGTTTTGCC/Dab/
F27-38-MB	/ThiolC6/CGACGAGCGCGTGTGGCCGGCGTGAGGGAGGTGAGTCG/MB/
F13-32-MB	/ThiolC6/CCATGGGTGTTTGCACTAAGTCCGGTTCTTGG/MB/

N30 represents random nucleotide; /Biotin/ represents biotin tag; /FAM/ represents fluorescein tag; /Dab/ represents dabcyl quencher tag; /ThiolC6/ represents thiol group with six-carbon spacer; /MB/ represents methylene blue redox tag.

Table S2. Selection strategy and conditions for fentanyl.

<table>
<tr><th>Round</th><th>Pool (pmol)</th><th>Wash steps[a]</th><th colspan="8">Counter-SELEX</th><th>Wash steps[b]</th><th>Target (μM)</th></tr>
<tr><td>1</td><td>1,000</td><td>10</td><td colspan="8">NA</td><td>NA</td><td>500</td></tr>
<tr><td>2</td><td>350</td><td>10</td><td colspan="4">Cocaine ×3 (100 μM)</td><td colspan="4">Heroin ×3 (100 μM)</td><td>30</td><td>500</td></tr>
<tr><td>3</td><td>350</td><td>30</td><td colspan="4">G1 ×3 (100 μM)</td><td colspan="4">G2 ×3 (100 μM)</td><td>30</td><td>250</td></tr>
<tr><td>4</td><td>300</td><td>30</td><td colspan="4">G1 ×3 (100 μM)</td><td colspan="4">G2 ×3 (100 μM)</td><td>30</td><td>250</td></tr>
<tr><td>5</td><td>300</td><td>30</td><td colspan="4">G1 ×3 (100 μM)</td><td colspan="4">G2 ×3 (100 μM)</td><td>30</td><td>250</td></tr>
<tr><td>6</td><td>300</td><td>30</td><td colspan="4">G1 ×3 (250 μM)</td><td colspan="4">G2 ×3 (250 μM)</td><td>30</td><td>250</td></tr>
<tr><td>7</td><td>300</td><td>30</td><td>G1 ×3
250 μM</td><td>G2 ×3
250 μM</td><td>G3 ×3
100 μM</td><td>G4 ×3
100 μM</td><td>G5 ×3
100 μM</td><td>G6 ×3
100 μM</td><td>G7 ×3
100 μM</td><td>G8 ×3
100 μM</td><td>30</td><td>200</td></tr>
<tr><td>8</td><td>200</td><td>30</td><td>G1 ×3
250 μM</td><td>G2 ×3
250 μM</td><td>G3 ×3
250 μM</td><td>G4 ×3
250 μM</td><td>G5 ×3
250 μM</td><td>G6 ×3
250 μM</td><td>G7 ×3
250 μM</td><td>G8 ×3
250 μM</td><td>30</td><td>100</td></tr>
<tr><td>9</td><td>200</td><td>30</td><td>G1 ×3
500 μM</td><td>G2 ×3
500 μM</td><td>G3 ×3
500 μM</td><td>G4 ×3
500 μM</td><td>G5 ×3
500 μM</td><td>G6 ×3
250 μM</td><td>G7 ×3
250 μM</td><td>G8 ×3
250 μM</td><td>30</td><td>75</td></tr>
<tr><td>10</td><td>200</td><td>30</td><td>G1 ×3
500 μM</td><td>G2 ×3
500 μM</td><td>G3 ×3
500 μM</td><td>G4 ×3
500 μM</td><td>G5 ×3
500 μM</td><td>G6 ×3
250 μM</td><td>G7 ×3
250 μM</td><td>G8 ×3
250 μM</td><td>30</td><td>50</td></tr>
<tr><td>11</td><td>200</td><td>30</td><td>G1 ×3
500 μM</td><td>G2 ×3
500 μM</td><td>G3 ×3
500 μM</td><td>G4 ×3
500 μM</td><td>G5 ×3
500 μM</td><td>G6 ×3
250 μM</td><td>G7 ×3
250 μM</td><td>G8 ×3
250 μM</td><td>30</td><td>50</td></tr>
</table>

The counter-targets were as follows:

G1 = cocaine, procaine, and lidocaine
G2 = heroin and quinine
G3 = acetaminophen, benzocaine, diphenhydramine, (+)-pseudoephedrine, and (+)-methamphetamine
G4 = codeine, morphine, and chlorpromazine
G5 = lactose, mannitol, and caffeine
G6 = lorazepam
G7 = papaverine
G8 = noscapine

Concentrations listed are for each chemical present in the group. Each wash consisted of 250 μL of 1× SELEX buffer. Wash 'a' refers to washes before counter-SELEX and wash 'b' refers to washes after counter-SELEX. We performed three washes with 1× SELEX buffer between each counter-target incubation, and three washes for each new counter target or counter target group.

Table S3. Selection strategy and conditions for acetyl fentanyl.

Round	Pool (pmol)	Wash steps[a]	Counter-SELEX	Wash steps[b]	Target (µM)
1	1,000	10	NA	NA	500
2	350	10	Cocaine ×3 (100 µM); Heroin ×3 (100 µM)	30	500
3	350	30	G1 ×3 (100 µM); G2 ×3 (100 µM)	30	250
4	300	30	G1 ×3 (100 µM); G2 ×3 (100 µM)	30	250
5	300	30	G1 ×3 100 µM; G2 ×3 100 µM; G6 ×3 250 µM; G7 ×3 250 µM; G8 ×3 250 µM	30	250
6	300	30	G1 ×3 250 µM; G2 ×3 250 µM; G6 ×3 250 µM; G7 ×3 250 µM; G8 ×3 250 µM	30	250
7	300	30	G1 ×3 250 µM; G2 ×3 250 µM; G3 ×3 250 µM; G4 ×3 100 µM; G5 ×3 100 µM; G6 ×5 250 µM; G7 ×5 250 µM; G8 ×5 250 µM	30	200
8	200	30	G1 ×3 250 µM; G2 ×3 250 µM; G3 ×3 250 µM; G4 ×3 250 µM; G5 ×3 250 µM; G6 ×10 250 µM; G7 ×10 250 µM; G8 ×10 250 µM	30	100
9	200	30	G1 ×3 500 µM; G2 ×3 500 µM; G3 ×3 500 µM; G4 ×3 500 µM; G5 ×3 500 µM; G6 ×10 250 µM; G7 ×10 250 µM; G8 ×10 250 µM	30	75
10	200	30	G1 ×3 500 µM; G2 ×3 500 µM; G3 ×3 500 µM; G4 ×3 500 µM; G5 ×3 500 µM; G6 ×10 250 µM; G7 ×10 250 µM; G8 ×10 250 µM	30	50

The counter-targets were as follows:

G1 = cocaine, procaine, and lidocaine
G2 = heroin and quinine
G3 = acetaminophen, benzocaine, diphenhydramine, (+)-pseudoephedrine, and (+)-methamphetamine
G4 = codeine, morphine, and chlorpromazine
G5 = lactose, mannitol, and caffeine
G6 = lorazepam
G7 = papaverine
G8 = noscapine

Concentrations listed are for each chemical present in the group. Each wash consisted of 250 µL of 1× SELEX buffer. Wash 'a' refers to washes before counter-SELEX and wash 'b' refers to washes after counter-SELEX. We performed three washes with 1× SELEX buffer between each counter-target incubation, and 3–10 washes for each new counter-target or counter target group.

Table S4. Selection strategy and conditions for furanyl fentanyl.

Round	Pool (pmol)	Wash steps[a]	Counter-SELEX								Wash steps[b]	Target (µM)
1	1,000	10	NA								NA	500
2	350	10	Cocaine ×3 (100 µM)				Heroin ×3 (100 µM)				30	500
3	350	30	G1 ×3<br>100 µM	G2 ×3<br>100 µM				G6 ×3<br>250 µM	G7 ×3<br>250 µM	G8 ×3<br>250 µM	30	250
4	300	30	G1 ×3<br>100 µM	G2 ×3<br>100 µM				G6 ×3<br>250 µM	G7 ×3<br>250 µM	G8 ×3<br>250 µM	30	250
5	300	30	G1 ×3<br>100 µM	G2 ×3<br>100 µM				G6 ×5<br>250 µM	G7 ×5<br>250 µM	G8 ×5<br>250 µM	30	250
6	300	30	G1 ×3<br>250 µM	G2 ×3<br>250 µM				G6 ×5<br>250 µM	G7 ×5<br>250 µM	G8 ×5<br>250 µM	30	250
7	300	30	G1 ×3<br>250 µM	G2 ×3<br>250 µM	G3 ×3<br>100 µM	G4 ×3<br>100 µM	G5 ×3<br>100 µM	G6 ×5<br>250 µM	G7 ×5<br>250 µM	G8 ×5<br>250 µM	30	200
8	200	30	G1 ×3<br>250 µM	G2 ×3<br>250 µM	G3 ×3<br>250 µM	G4 ×3<br>250 µM	G5 ×3<br>250 µM	G6 ×10<br>250 µM	G7 ×10<br>250 µM	G8 ×10<br>250 µM	30	100
9	200	30	G1 ×3<br>500 µM	G2 ×3<br>500 µM	G3 ×3<br>500 µM	G4 ×3<br>500 µM	G5 ×3<br>500 µM	G6 ×10<br>250 µM	G7 ×10<br>250 µM	G8 ×10<br>250 µM	30	50
10	200	30	G1 ×3<br>500 µM	G2 ×3<br>500 µM	G3 ×3<br>500 µM	G4 ×3<br>500 µM	G5 ×3<br>500 µM	G6 ×10<br>250 µM	G7 ×10<br>250 µM	G8 ×10<br>250 µM	30	25

The counter-targets were as follows:

G1 = cocaine, procaine, and lidocaine
G2 = heroin and quinine
G3 = acetaminophen, benzocaine, diphenhydramine, (+)-pseudoephedrine, and (+)-methamphetamine
G4 = codeine, morphine, and chlorpromazine
G5 = lactose, mannitol, and caffeine
G6 = lorazepam
G7 = papaverine
G8 = noscapine

Concentrations listed are for each chemical present in the group. Each wash consisted of 250 µL of 1× SELEX buffer. Wash 'a' refers to washes before counter-SELEX and wash 'b' refers to washes after counter-SELEX. We performed three washes with 1× SELEX buffer between each counter-target incubation, and 3–10 washes for each new counter-target or counter target group.

Table S5. Major digestion products of the 16 selected aptamer candidates.

Aptamer	Sequence (5'–3')
F1-40	CTTACGACACGAGGTGTTTGGACTAAGTTCGGTTTCGGGT
F2-38	CTTACGACGACTGCGTGTGGCCGGTGTGAGGGAGGGTT
F3-37	CTTACGACAGCGGGTGTATGTACTAAGTCCGGTTCGG
F5-40	CTTACGACCAGGCCTACGGAAGCAGCGTCAGCGGGGGGGT
F7-40	CTTACGACCACCATGGGAATCGGGTGGCTTGGAGGTGCGT
F8-42	CTTACGACGAGCATCGGTTTTTTCGGTGATGTCTGGGAGTCG
F10-37	CTTACGACGGCAGGTGTTTGCACTAAGTCCGGTATGT
F12-38	CTTACGACATCTGCGTGTGGCCGGTGTGAGGGAGGGAT
F13-39	CTTACGACCATGGGTGTTTGCACTAAGTCCGGTTCTTGG
F16-43	CTTACGACCGGCGGAAGGCTGGAGGGGTTGGGGGAGGTGTCGT
F18-43	CTTACGACCGGGATCCTTTGGGACAACCTGGTGGGCATGTCGT
F20-40	CTTACGACGAAGCAACGGGGTTTCGGAGGGCAGGTGTCGT
F21-35	CTTACGACCGGACATGTGATCGGGCAGCTGGGAGT
F22-42	CTTACGACGTCGAGGGGTACCCTTTGGCGTTCGTCGAGGTCG
F23-40	CTTACGACCAGGCTACGTGGGGGAGGGTGGGAAGACGGGT
F27-42	CTTACGACGAGCGCGTGTGGCCGGCGTGAGGGAGGTGAGTCG

Table S6. Aptamer dissociation constants (K_D), and ITC experiment conditions.

Aptamer	Ligand type	[Ligand] (μM)	[Aptamer] (μM)	K_D (nM)
F1	Fentanyl	200	20	510 ± 13
F2	Fentanyl	150	15	93 ± 5
F3	Fentanyl	200	20	709 ± 37
F4	Fentanyl	200	20	923 ± 41
F5	Fentanyl	150	20	316 ± 17
F6	Fentanyl	100	10	42 ± 4
F7	Fentanyl	100	10	43 ± 5
F8	Fentanyl	100	10	350 ± 22
F9	Fentanyl	100	10	72 ± 9
F21	Fentanyl	150	20	310 ± 10
F28	Fentanyl	100	10	43 ± 4
F10	Acetyl fentanyl	200	20	546 ± 25
F11	Acetyl fentanyl	1,000	40	27,200 ± 650
F12	Acetyl fentanyl	65	7.5	60 ± 7
F13	Acetyl fentanyl	200	20	251 ± 15
F14	Acetyl fentanyl	200	20	68 ± 4
F15	Acetyl fentanyl	100	10	156 ± 10
F16	Acetyl fentanyl	50	10	94 ± 18
F17	Acetyl fentanyl	100	10	98 ± 7
F18	Acetyl fentanyl	100	10	29 ± 3
F27	Acetyl fentanyl	80	10	21 ± 5
F19	Furanyl fentanyl	250	20	NA
F20	Furanyl fentanyl	100	10	207 ± 14
F21	Furanyl fentanyl	150	15	193 ± 18
F22	Furanyl fentanyl	100	10	240 ± 22
F23	Furanyl fentanyl	100	10	51 ± 3
F24	Furanyl fentanyl	100	10	63 ± 11
F25	Furanyl fentanyl	100	10	171 ± 11
F26	Furanyl fentanyl	150	10	59 ± 7
F27	Furanyl fentanyl	50	10	4 ± 1
F28	Furanyl fentanyl	60	7.5	62 ± 9
F2	Furanyl fentanyl	100	10	53 ± 12
F6	Furanyl fentanyl	100	10	63 ± 7

Table S7. Aptamer digestion product dissociation constants (K_D) and ITC experiment conditions.

Aptamer ID	Target type	[Ligand] (μM)	[Aptamer] (μM)	Digestion product K_D (nM)	Parent K_D (nM)	K_D ratio of Product/ Parent
F1-40	Fentanyl	1,000	50	3,730 ± 110	510 ± 13	7.3
F2-38	Fentanyl	1,400	80	40,600 ± 5627	93 ± 5	436.6
F3-37	Fentanyl	1,000	40	64,900 ± 2100	709 ± 37	91.5
F5-40	Fentanyl	150 × 2 (2 titrations)	20	2,808 ± 377	316 ± 17	8.9
F7-40	Fentanyl	1,000	40	15,800 ± 1300	43 ± 5	367.4
F8-42	Fentanyl	100	10	165 ± 16	350 ± 22	0.5
F21-35	Fentanyl	1,000	40	NA	310 ± 10	NA
F10-37	Acetyl fentanyl	1,500	100	91,700 ± 3700	546 ± 25	167.9
F12-38	Acetyl fentanyl	600	60	49,020 ± 1100	60 ± 7	817
F13-39	Acetyl fentanyl	100	10	6,622 ± 112	251 ± 15	26.4
F16-43	Acetyl fentanyl	100	20	59 ± 12	94 ± 18	0.6
F18-43	Acetyl fentanyl	100	10	17 ± 4	29 ± 3	0.6
F27-42	Acetyl fentanyl	200	30	225 ± 45	21 ± 5	10.7
F2-38	Furanyl fentanyl	300 × 5 (5 titrations)	60	5,747 ± 293	53 ± 12	108.4
F20-40	Furanyl fentanyl	400	40	6,450 ± 449	207 ± 14	31.2
F21-35	Furanyl fentanyl	400	40	NA	193 ± 18	NA
F22-42	Furanyl fentanyl	400 × 2 (2 titrations)	40	4,761 ± 412	240 ± 22	19.8
F23-40	Furanyl fentanyl	100	10	523 ± 67	51 ± 3	10.3
F27-42	Furanyl fentanyl	200	30	92 ± 20	4 ± 1	23
F2-38	Furanyl fentanyl	300 × 5	60	5747 ± 293	53 ± 12	108.4
F13-39	Fentanyl	320	20	5,550 ± 285		
F27-42	Fentanyl	200	30	142 ± 20		

Table S8. Final fitted parameters of K_D-R_{value} plots.

**Target (μM)**	**$E_{1/2}$**	**n**	**Adj. R squared**
1.5	27 ± 4	1.0 ± 0.2	0.875
5	54 ± 9	0.8 ± 0.06	0.910
25	119 ± 32	0.4 ± 0.05	0.917
100	467 ± 160	0.3 ± 0.04	0.931

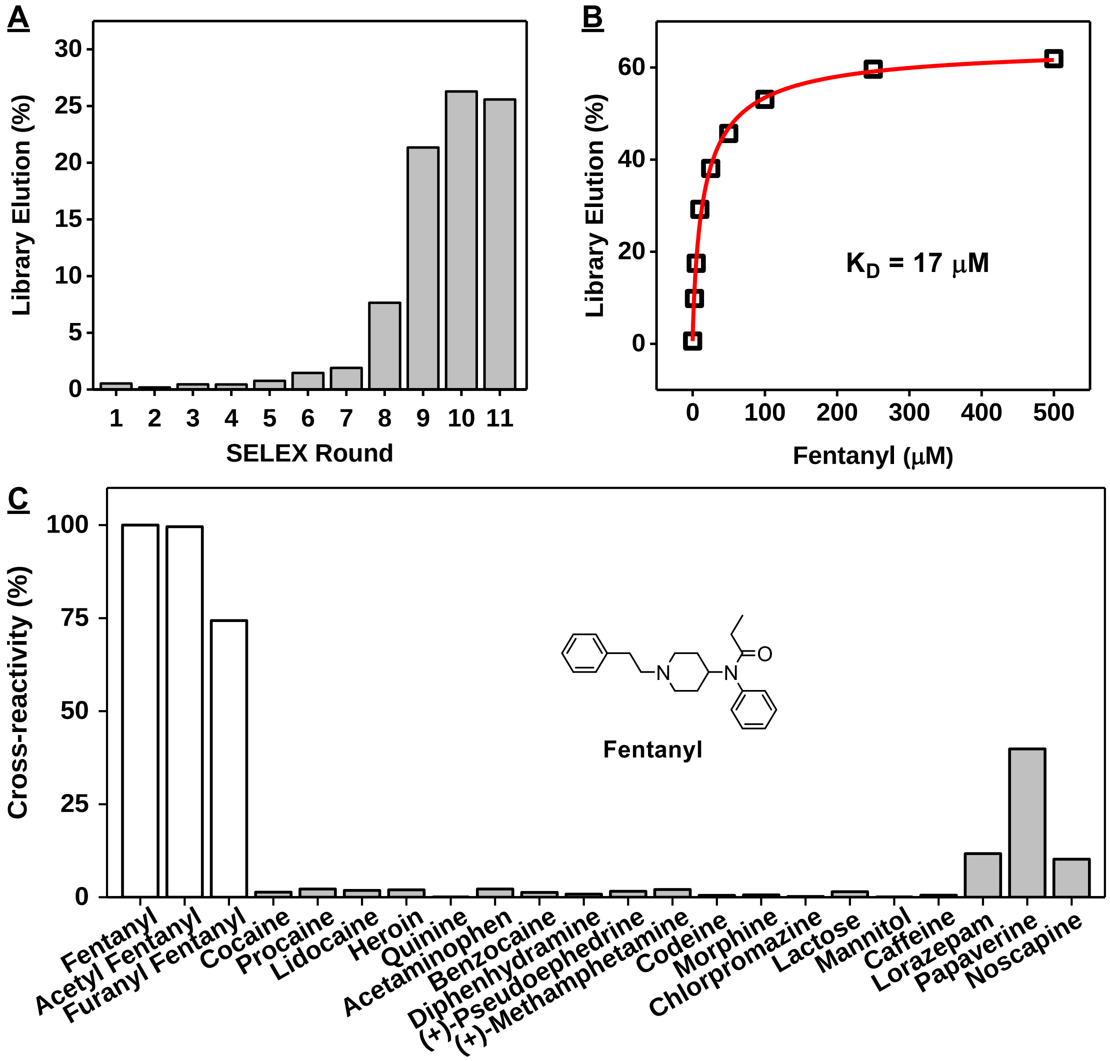

Figure S1. Isolation of fentanyl-binding aptamers via library-immobilized SELEX. (**A**) Percent of pool eluted by the target in each selection round. (**B**) Determination of binding affinity of the round 11 fentanyl pool using a gel-elution assay,(1) where the percent of the pool eluted was plotted against fentanyl concentration (0, 2.5, 5, 10, 25, 50, 100, 250, and 500 μM). (**C**) Determination of the cross-reactivity of the round 11 fentanyl pool against 25 μM fentanyl, acetyl fentanyl, or furanyl fentanyl or 250 μM cocaine, procaine, lidocaine, heroin, quinine, acetaminophen, benzocaine, diphenhydramine, (+)-pseudoephedrine, (+)-methamphetamine, codeine, morphine, chlorpromazine, lactose, mannitol, caffeine, lorazepam, papaverine, or noscapine. Cross-reactivity is calculated relative to 25 μM fentanyl.

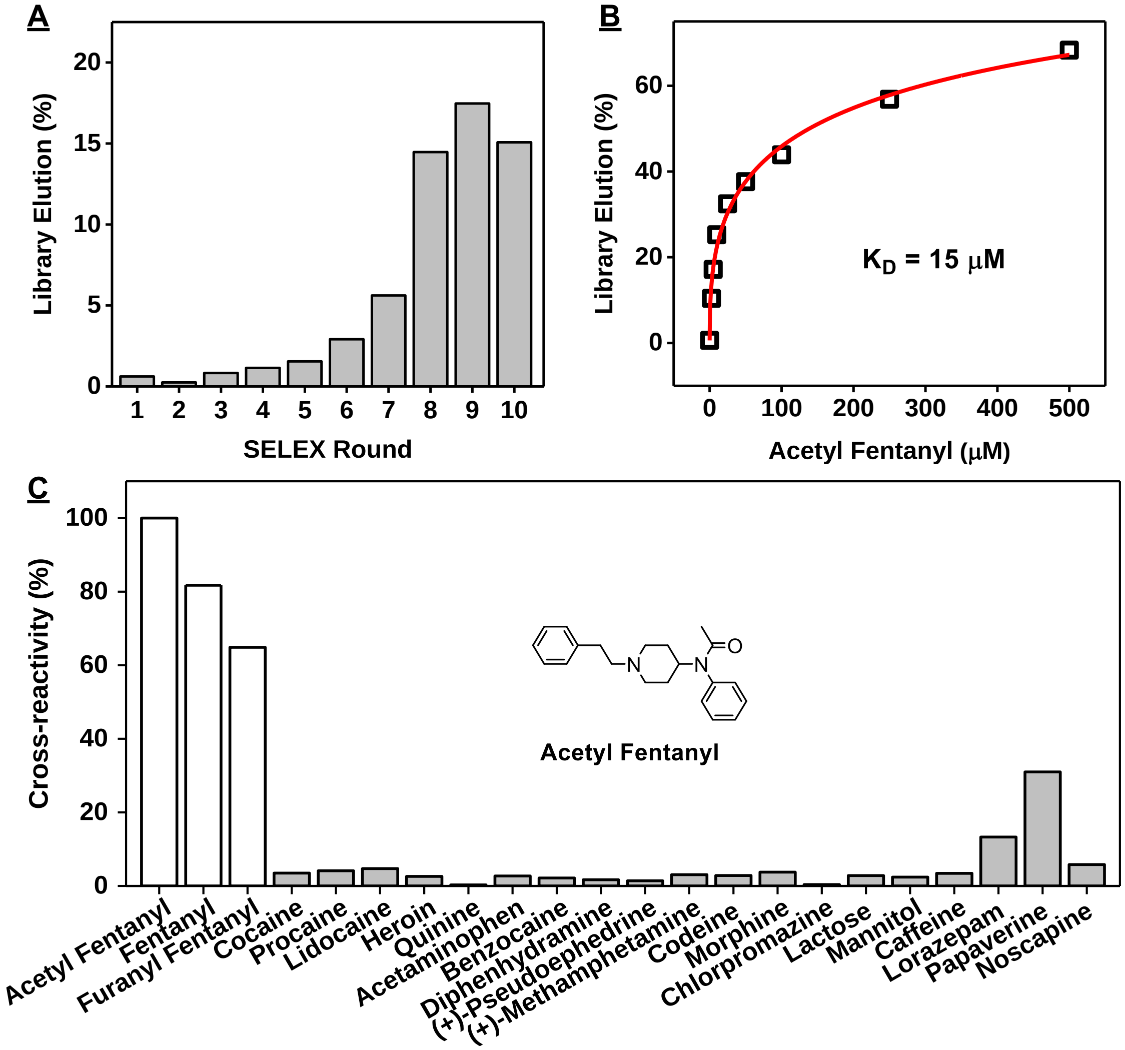

Figure S2. Isolation of acetyl fentanyl-binding aptamers via library-immobilized SELEX. (**A**) Percent of pool eluted by the target in each selection round. (**B**) Determination of binding affinity of the round 10 acetyl fentanyl pool using a gel-elution assay(1), where the percent of the pool eluted by acetyl fentanyl was plotted against acetyl fentanyl concentration (0, 2.5, 5, 10, 25, 50, 100, 250, and 500 µM). **C**) Determination of the cross-reactivity of the round 10 acetyl fentanyl pool against 25 µM acetyl fentanyl, fentanyl, or furanyl fentanyl, or 250 µM cocaine, procaine, lidocaine, heroin, quinine, acetaminophen, benzocaine, diphenhydramine, (+)-pseudoephedrine, (+)-methamphetamine, codeine, morphine, chlorpromazine, lactose, mannitol, caffeine, lorazepam, papaverine, or noscapine. Cross-reactivity is calculated relative to 25 µM acetyl fentanyl.

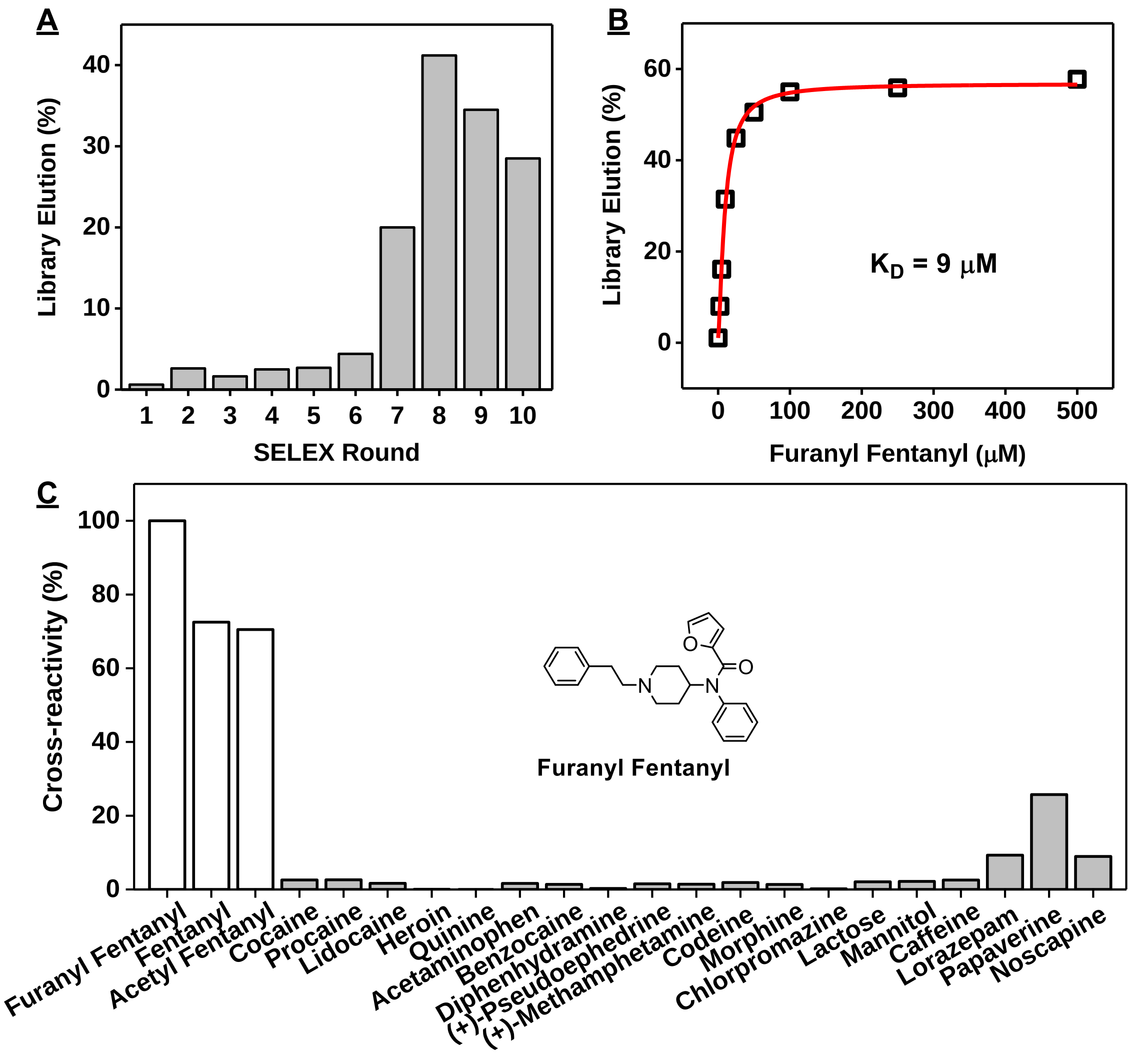

Figure S3. Isolation of furanyl fentanyl-binding aptamers via library-immobilized SELEX. (**A**) Percent of pool eluted by the target in each selection round. (**B**) Determination of binding affinity of the round 10 furanyl fentanyl pool using a gel-elution assay(1), where the percent of the pool eluted by furanyl fentanyl was plotted against furanyl fentanyl concentration (0, 2.5, 5, 10, 25, 50, 100, 250, and 500 µM). **C**) Determination of the cross-reactivity of the round 10 furanyl fentanyl pool against 25 µM furanyl fentanyl, fentanyl, or acetyl fentanyl, or 250 µM cocaine, procaine, lidocaine, heroin, quinine, acetaminophen, benzocaine, diphenhydramine, (+)-pseudoephedrine, (+)-methamphetamine, codeine, morphine, chlorpromazine, lactose, mannitol, caffeine, lorazepam, papaverine, or noscapine. Cross-reactivity is calculated relative to 25 µM furanyl fentanyl.

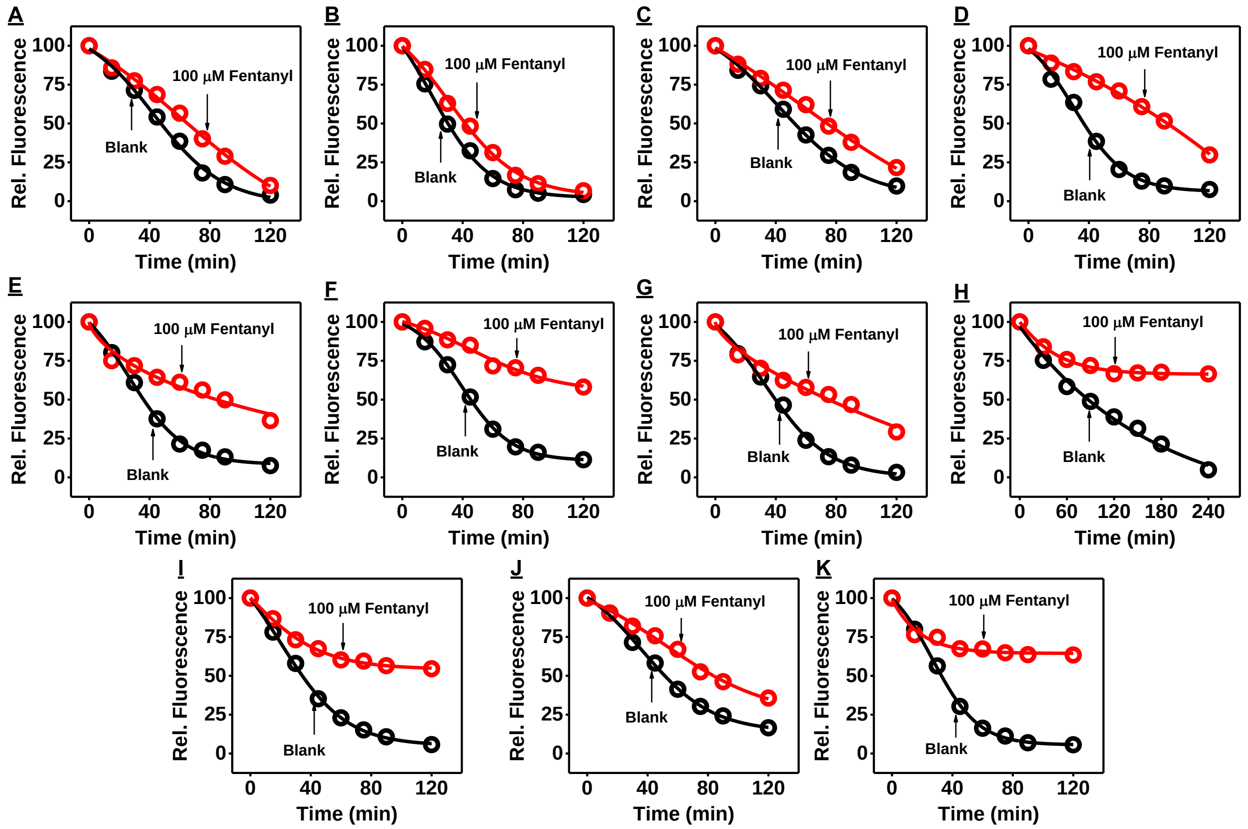

Figure S4. Digestion time-course of fentanyl aptamer candidates (**A**) F1, (**B**) F2, (**C**) F3, (**D**) F4, (**E**) F5, (**F**) F6, (**G**) F7, (**H**) F8, (**I**) F9, (**J**) F21, and (**K**) F28 in the absence and presence of 100 μM fentanyl.

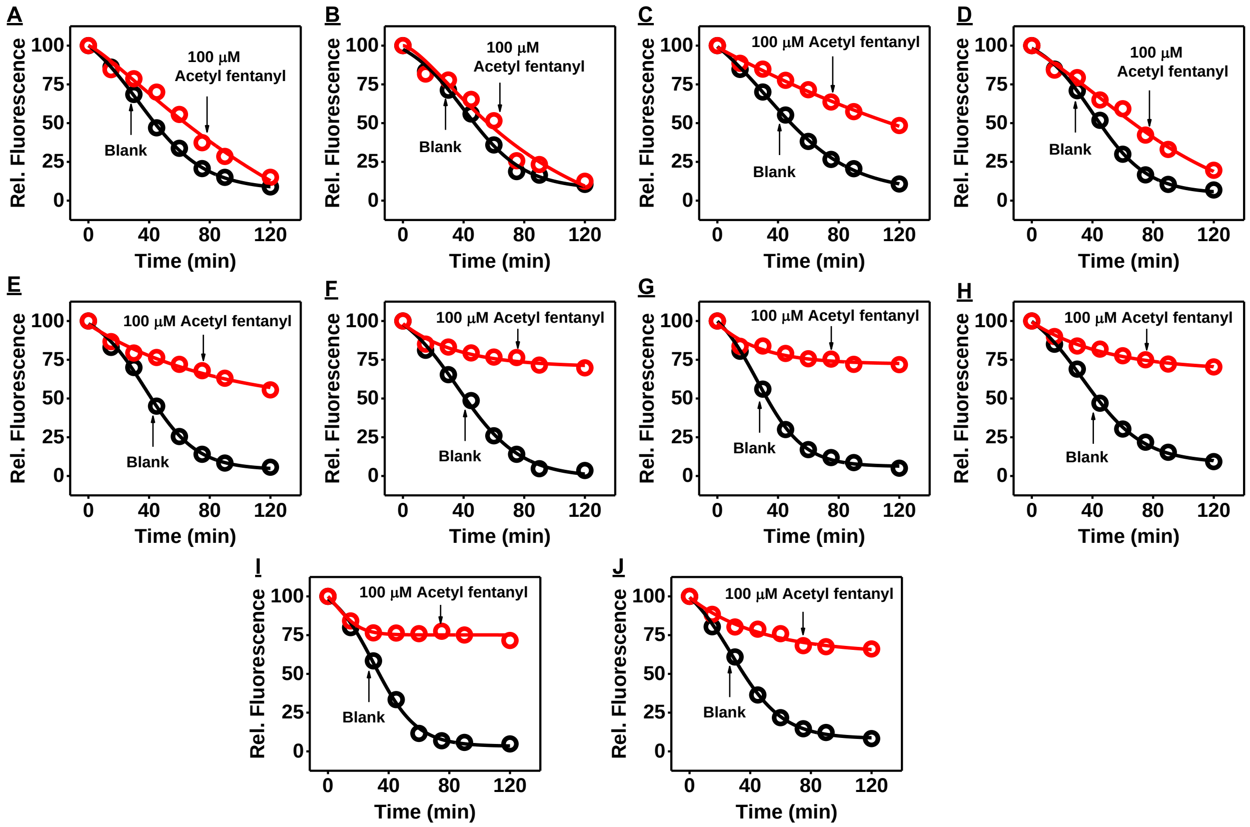

Figure S5. Digestion time-course of acetyl fentanyl aptamer candidates (**A**) F10, (**B**) F11, (**C**) F12, (**D**) F13, (**E**) F14, (**F**) F15, (**G**) F16, (**H**) F17, (**I**) F18, and (**J**) F27 in the absence and presence of 100 µM acetyl fentanyl.

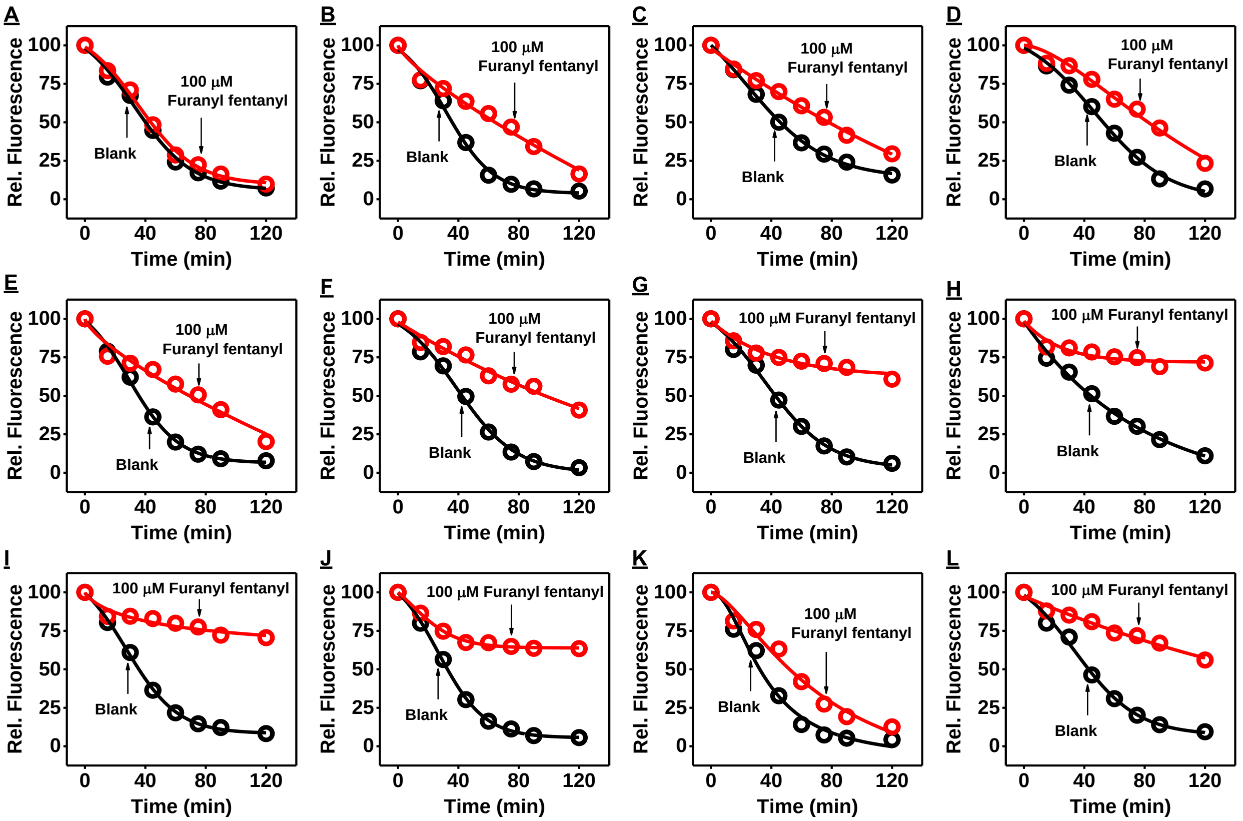

Figure S6. Digestion time-course of furanyl fentanyl aptamer candidates (**A**) F19, (**B**) F20, (**C**) F21, (**D**) F22, (**E**) F23, (**F**) F24, (**G**) F25, (**H**) F26, (**I**) F27, (**J**) F28, (**K**) F2, and (**L**) F6 in the absence and presence of 100 µM furanyl fentanyl.

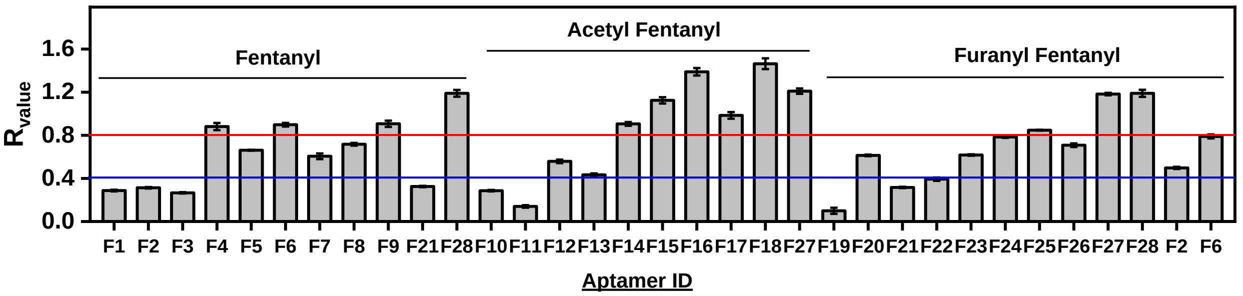

Figure S7. Resistance value (R_{value}) obtained from digestion of aptamer candidates in the presence of 100 µM of the respective selection targets plotted in **Figures S4–S6**.

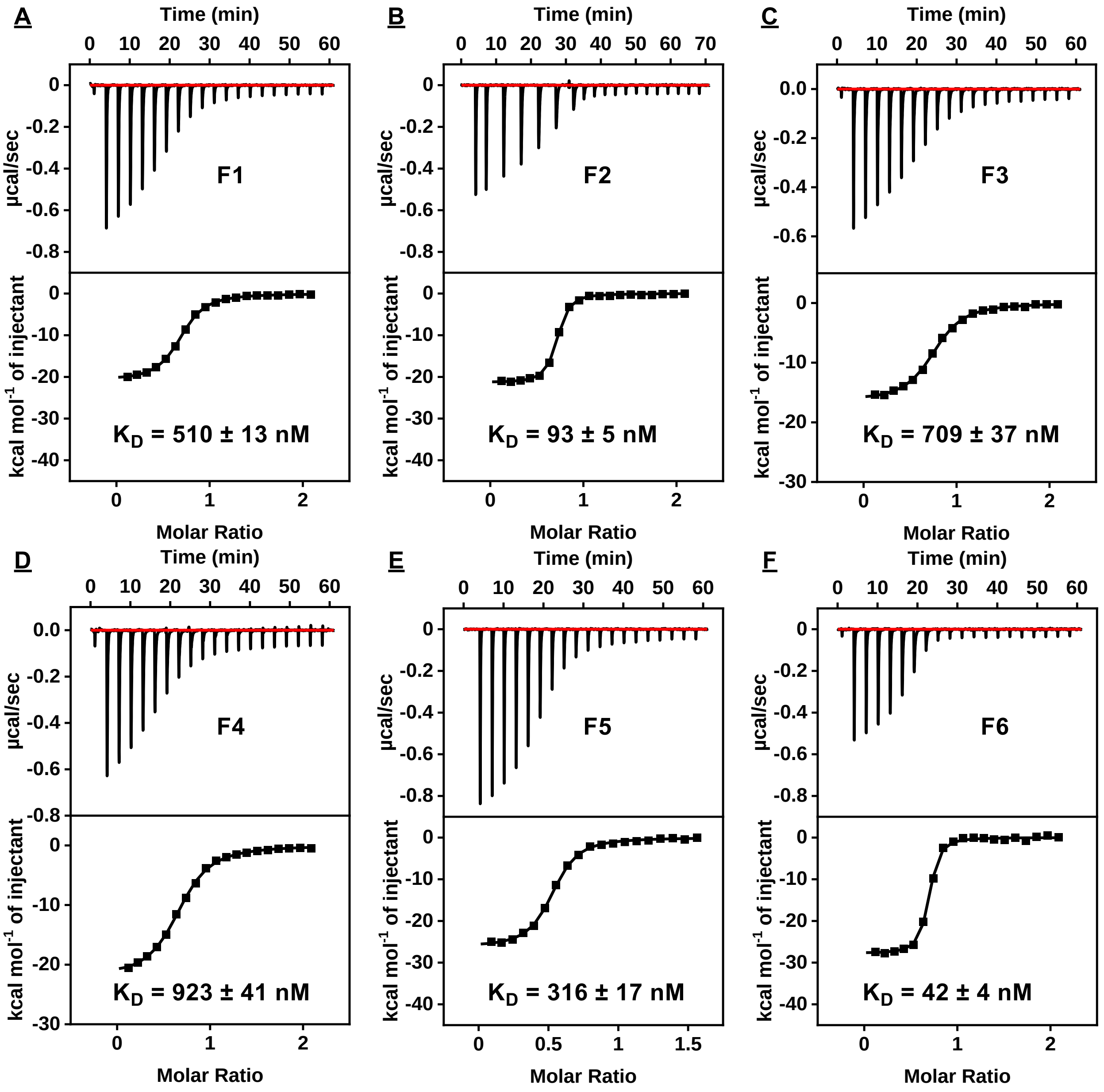

Figure S8. Characterization of fentanyl binding affinity of six aptamer candidates using ITC. Top panels display the heat generated from each titration of fentanyl into (**A**) F1, (**B**) F2, (**C**) F3, (**D**) F4, (**E**) F5, and (**F**) F6. Bottom panels show the integrated heat of each titration after correcting for the heat of dilution of the titrant.

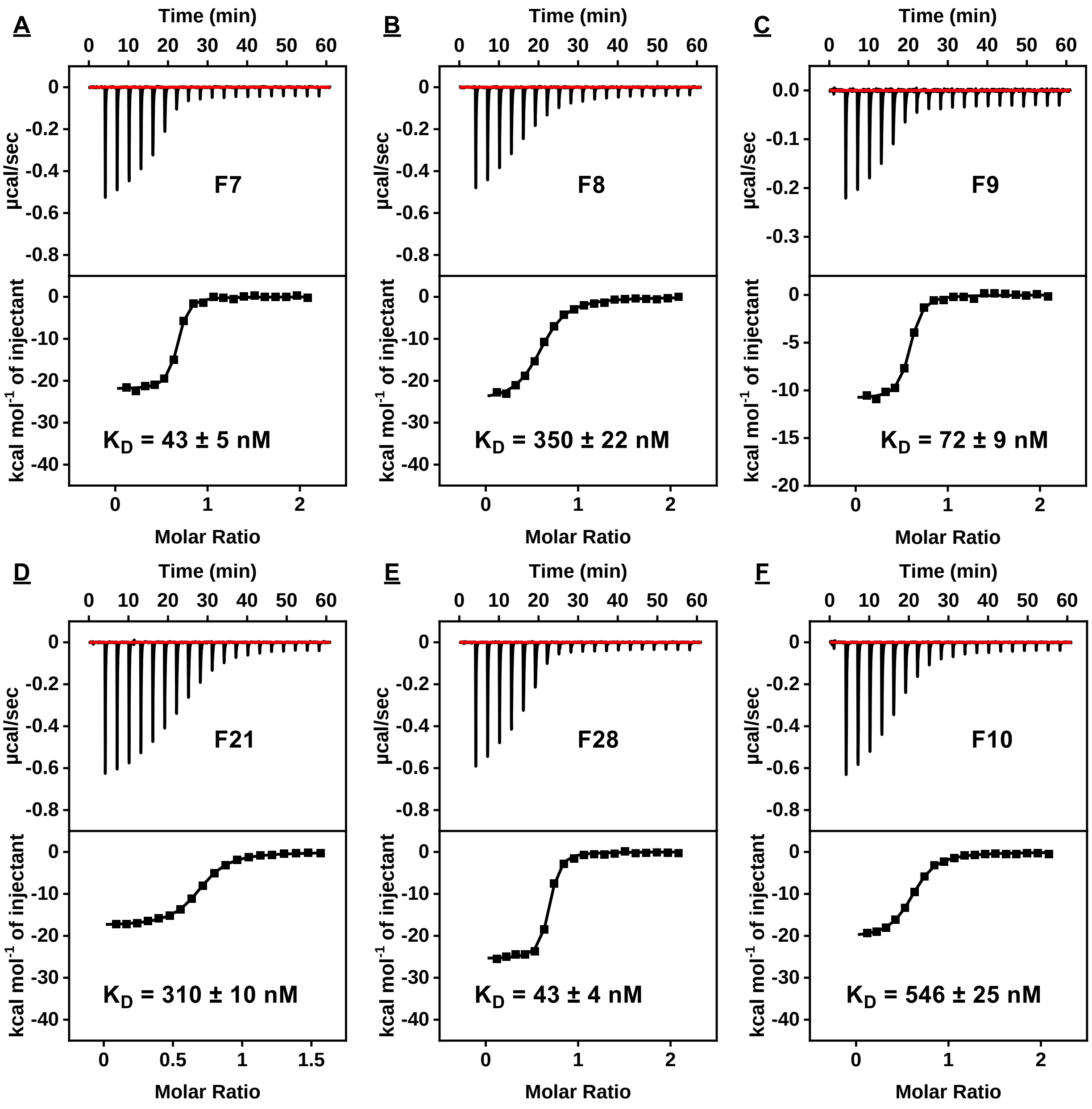

Figure S9. Characterization of fentanyl- and acetyl fentanyl-binding affinity of six aptamer candidates using ITC. Top panels display the heat generated from each titration of fentanyl into (**A**) F7, (**B**) F8, (**C**) F9, (**D**) 21, and (**E**) F28, or acetyl fentanyl into (**F**) F10. Bottom panels show the integrated heat of each titration after correcting for the heat of dilution of the titrant.

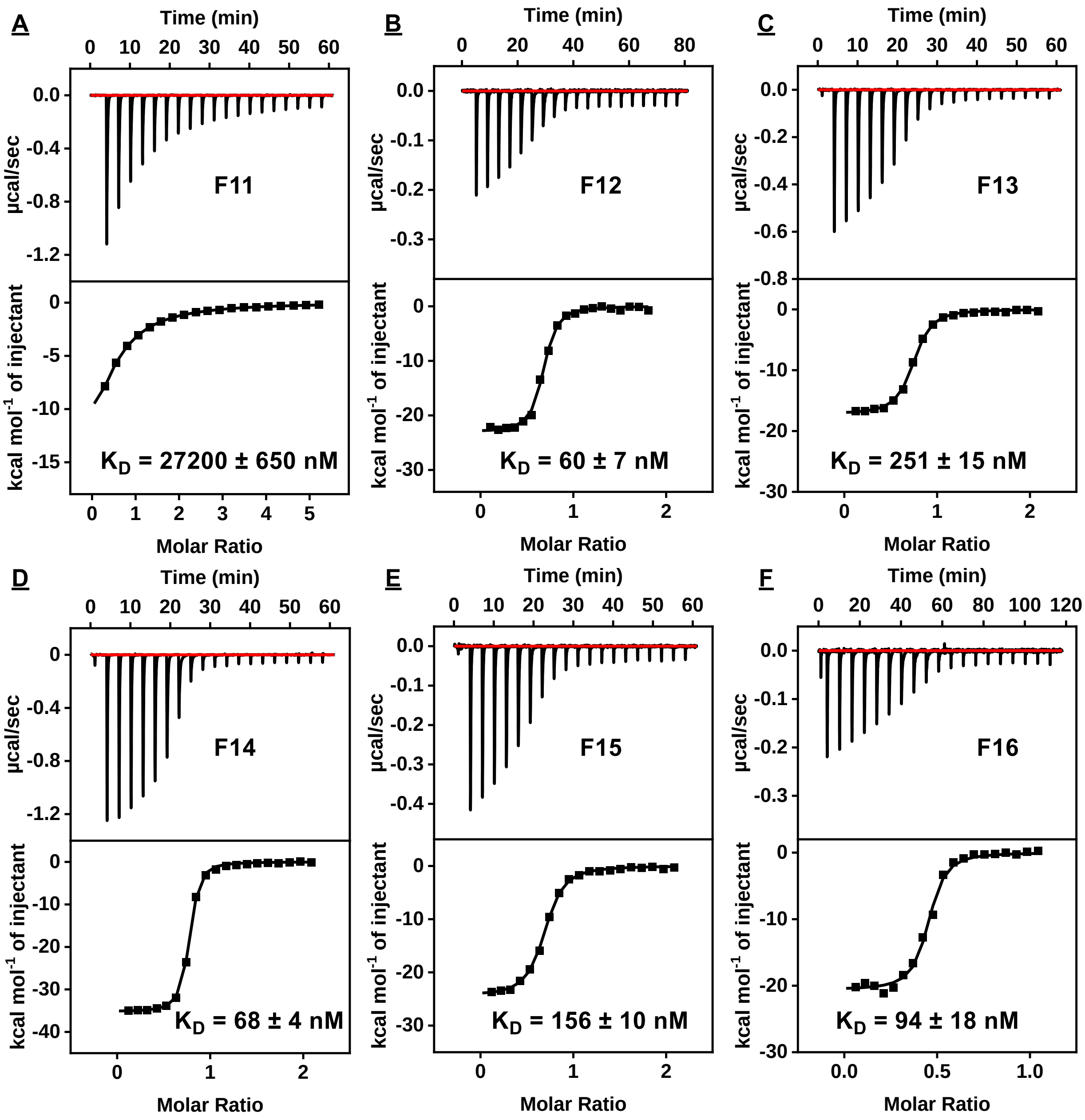

Figure S10. Characterization of acetyl fentanyl binding affinity of six aptamer candidates using ITC. Top panels display the heat generated from each titration of acetyl fentanyl into (**A**) F11, (**B**) F12, (**C**) F13, (**D**) F14, (**E**) F15, and (**F**) F16. Bottom panels show the integrated heat of each titration after correcting for the heat of dilution of the titrant.

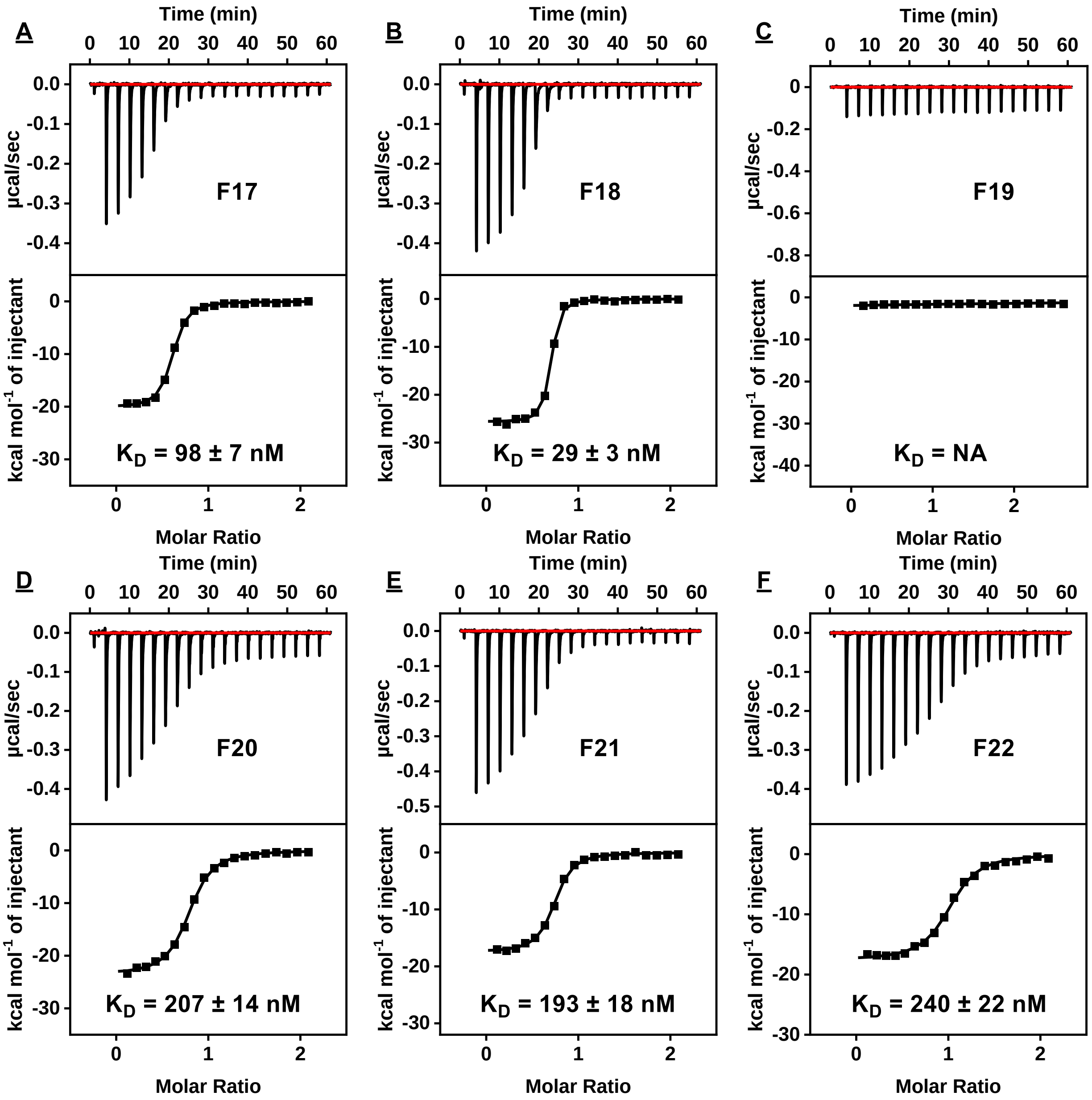

Figure S11. Characterization of acetyl fentanyl- and furanyl fentanyl-binding affinity of six aptamer candidates using ITC. Top panels display the heat generated from each titration of acetyl fentanyl into (**A**) F17 and (**B**) F18, or furanyl fentanyl into (**C**) F19, (**D**) F20, (**E**) F21, and (**F**) F22. Bottom panels show the integrated heat of each titration after correcting for the heat of dilution of the titrant.

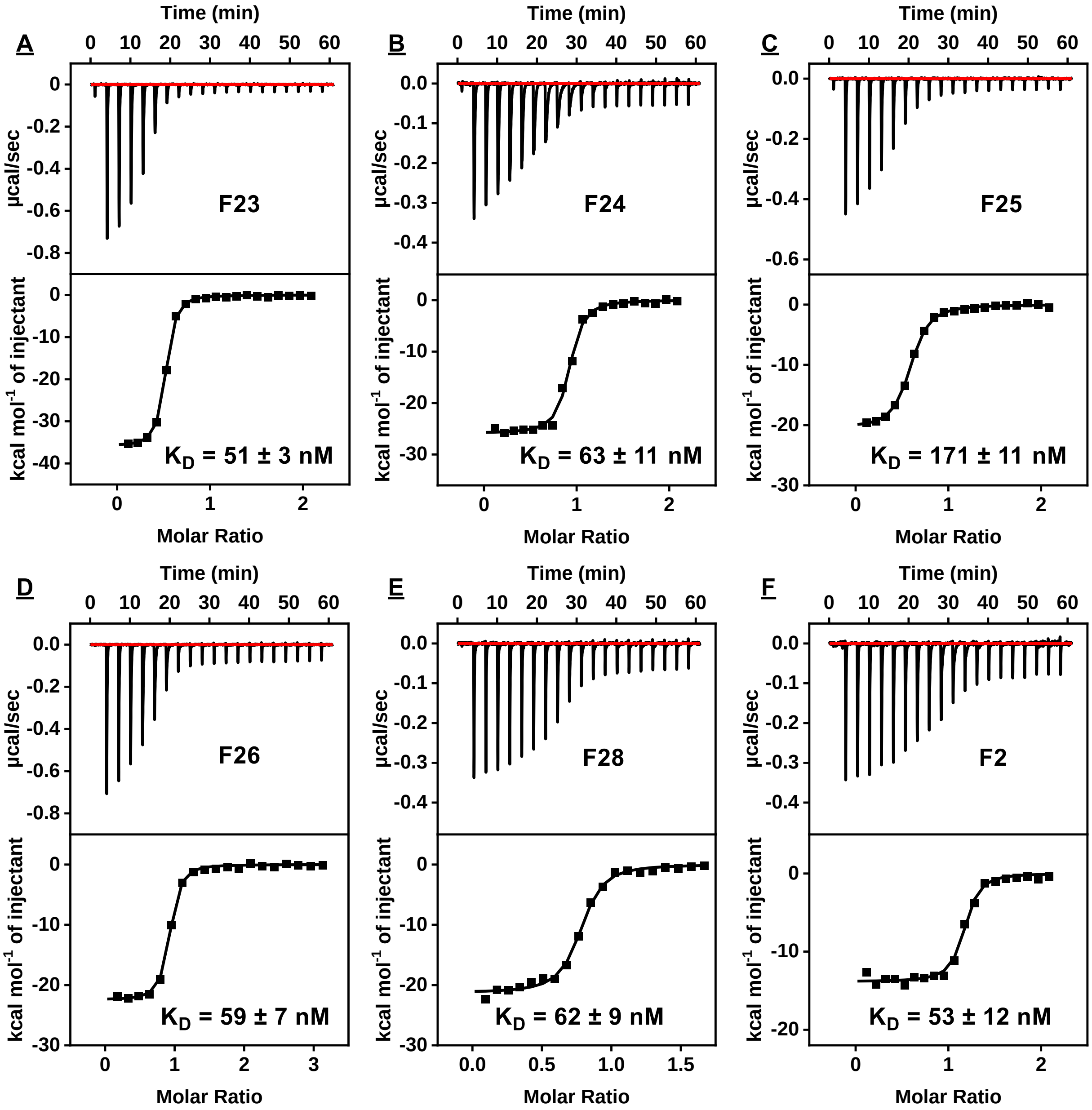

Figure S12. Characterization of furanyl fentanyl binding affinity of six aptamer candidates using ITC. Top panels display the heat generated from each titration of furanyl fentanyl into (**A**) F23, (**B**) F24, (**C**) F25, (**D**) F26, (**E**) F28, and (**F**) F2. Bottom panels show the integrated heat of each titration after correcting for the heat of dilution of the titrant.

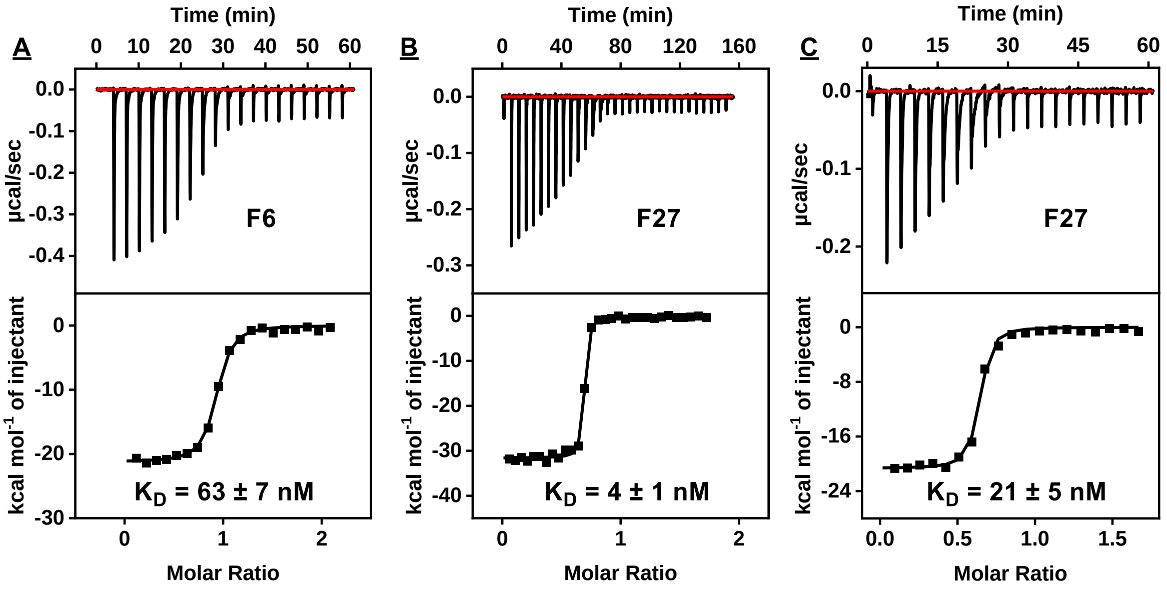

Figure S13. Characterization of furanyl fentanyl binding affinity of aptamer candidates using ITC. Top panels display the heat generated from each titration of furanyl fentanyl into (**A**) F6 and (**B**) F27 as well as from each titration of acetyl fentanyl into (**C**) F27. Bottom panels show the integrated heat of each titration after correcting for the heat of dilution of the titrant.

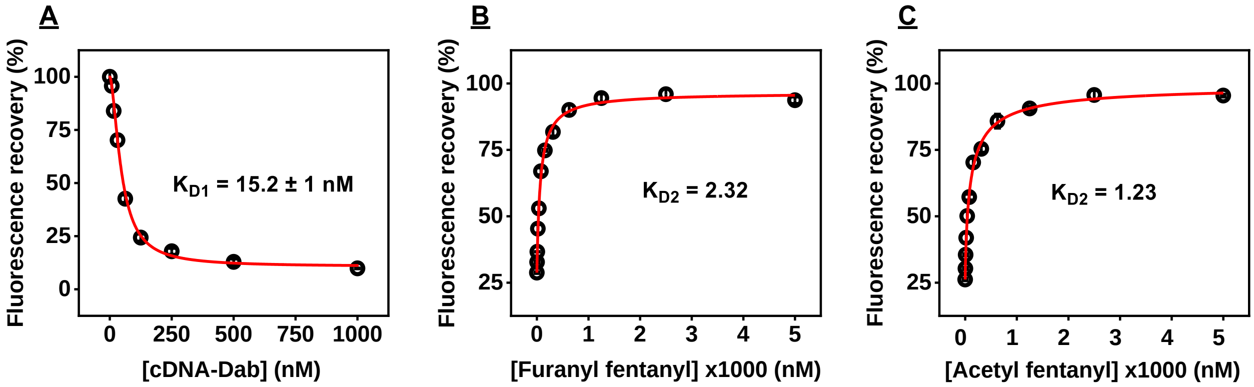

Figure S14. Determination of F27 furanyl fentanyl- and acetyl fentanyl-binding affinity using a fluorescence strand-displacement assay. (**A**) K_{D1} was determined by mixing different concentrations of cDNA-Dab with 50 nM F27-FAM and measuring the fluorescence quenching at 520 nm. F27-FAM was incubated with 0, 8, 16, 31, 62.5, 125, 250, 500, or 1,000 nM cDNA-Dab. K_{D2} was determined from the fluorescence recovery at 520 nm of 50 nM F27-FAM/125 nM cDNA-Dab complexes upon addition of varying concentrations of (**B**) furanyl fentanyl or (**C**) acetyl fentanyl. Target concentrations used were 0, 5, 10, 20, 39, 78, 156, 312.5, 625, 1,250, 2,500, and 5,000 nM.

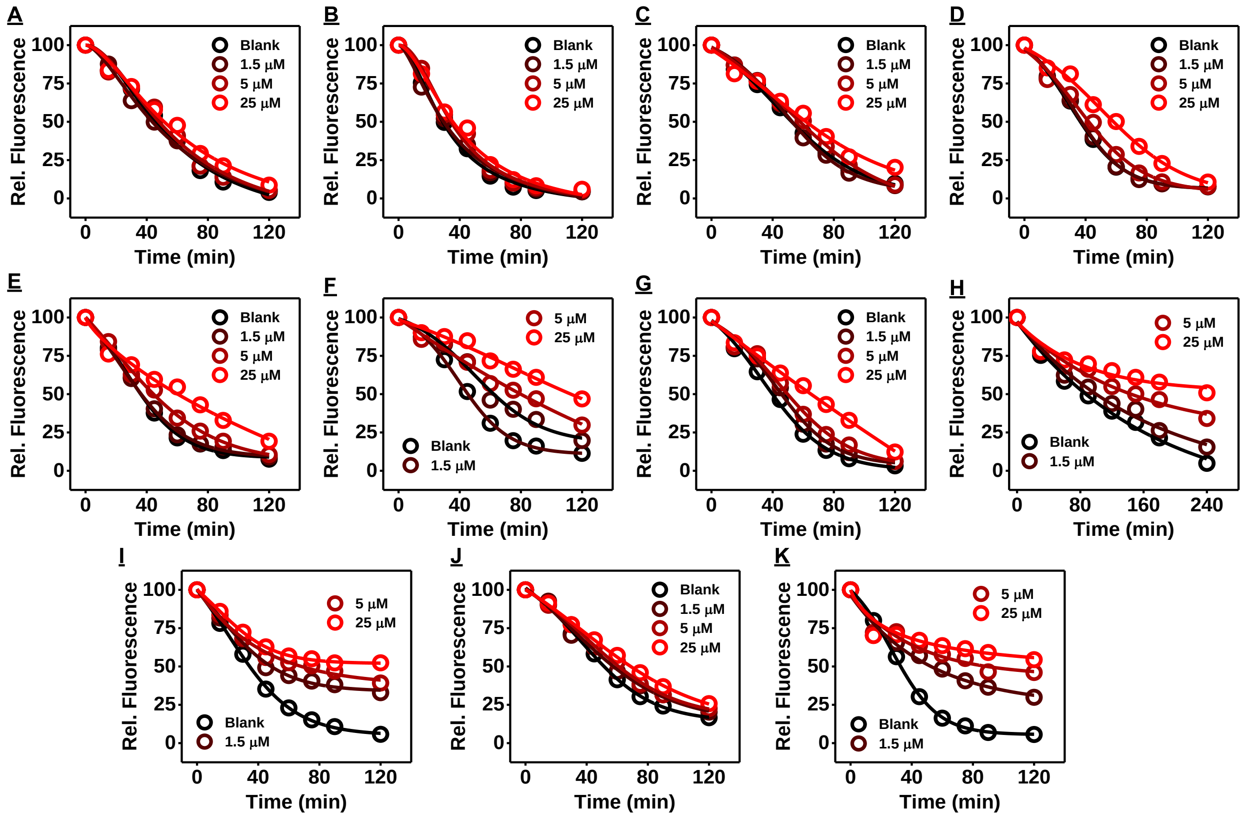

Figure S15. Exonuclease inhibition at low concentrations of fentanyl. Time-course digestion of (**A**) F1, (**B**) F2, (**C**) F3, (**D**) F4, (**E**) F5, (**F**) F6, (**G**) F7, (**H**) F8, (**I**) F9, (**J**) F21, and (**K**) F28 in the absence and presence of 1.5, 5, and 25 μM fentanyl.

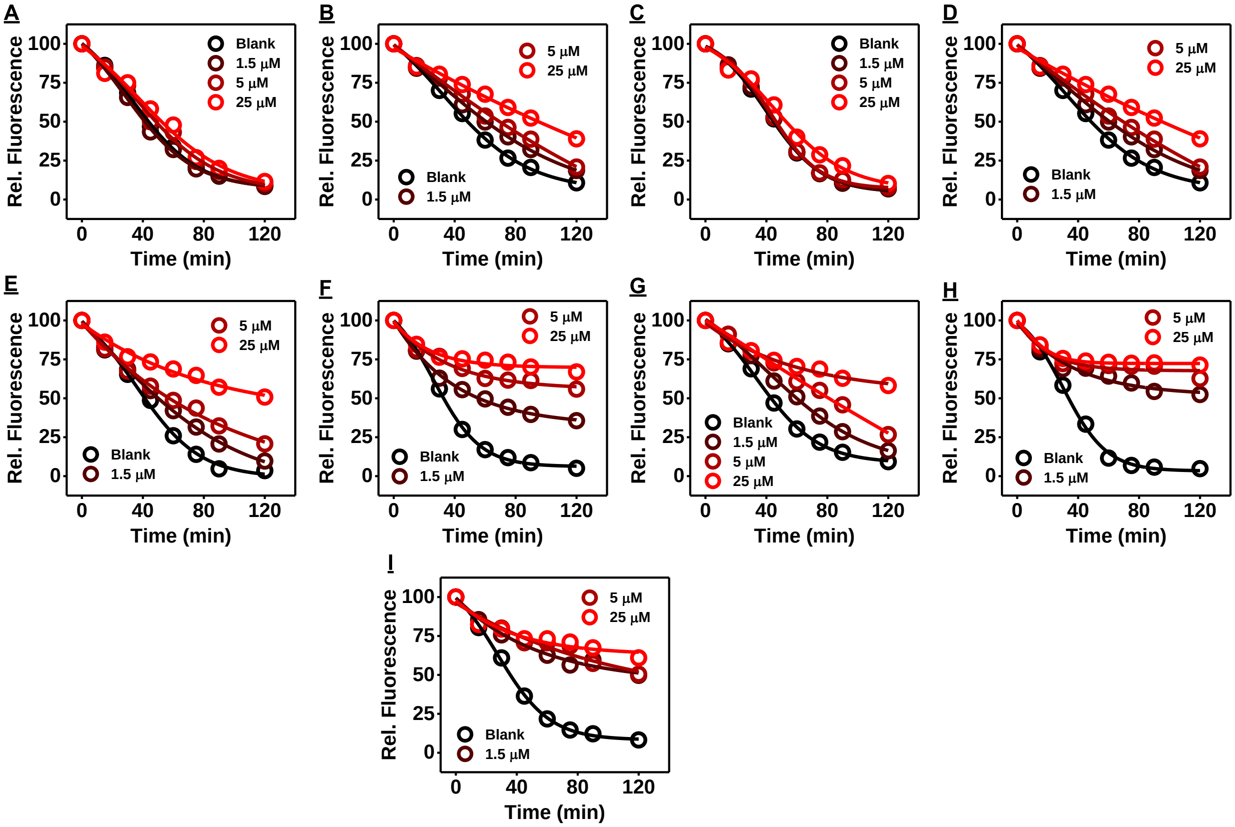

Figure S16. Exonuclease inhibition at low concentrations of acetyl fentanyl. Time-course digestion of (**A**) F10, (**B**) F12, (**C**) F13, (**D**) F14, (**E**) F15, (**F**) F16, (**G**) F17, (**H**) F18, and (**I**) F27 in the absence and presence of 1.5, 5, and 25 μM acetyl fentanyl.

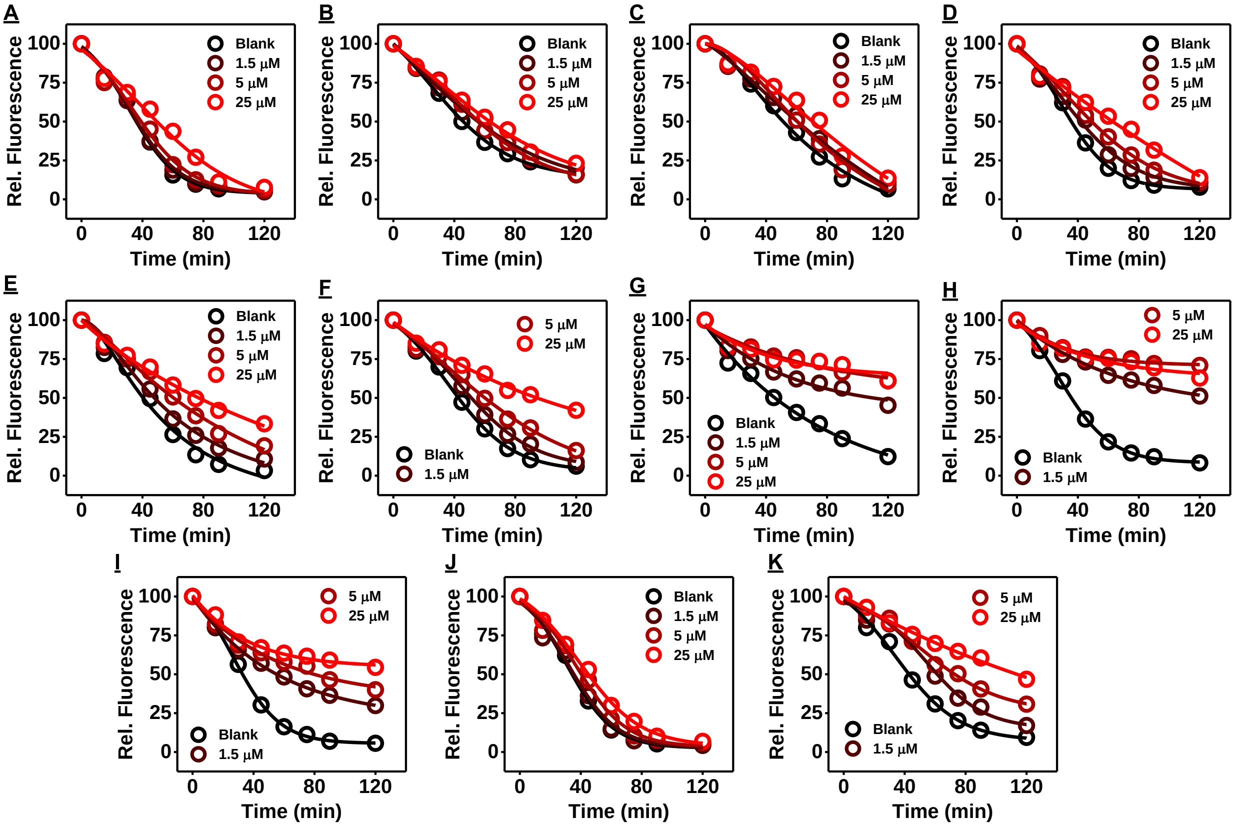

Figure S17. Exonuclease inhibition at low concentrations of furanyl fentanyl. Time-course digestion of (**A**) F20, (**B**) F21, (**C**) F22, (**D**) F23, (**E**) F24, (**F**) F25, (**G**) F26, (**H**) F27, (**I**) F28, (**J**) F2, and (**K**) F6 in the absence and presence of 1.5, 5, and 25 μM furanyl fentanyl.

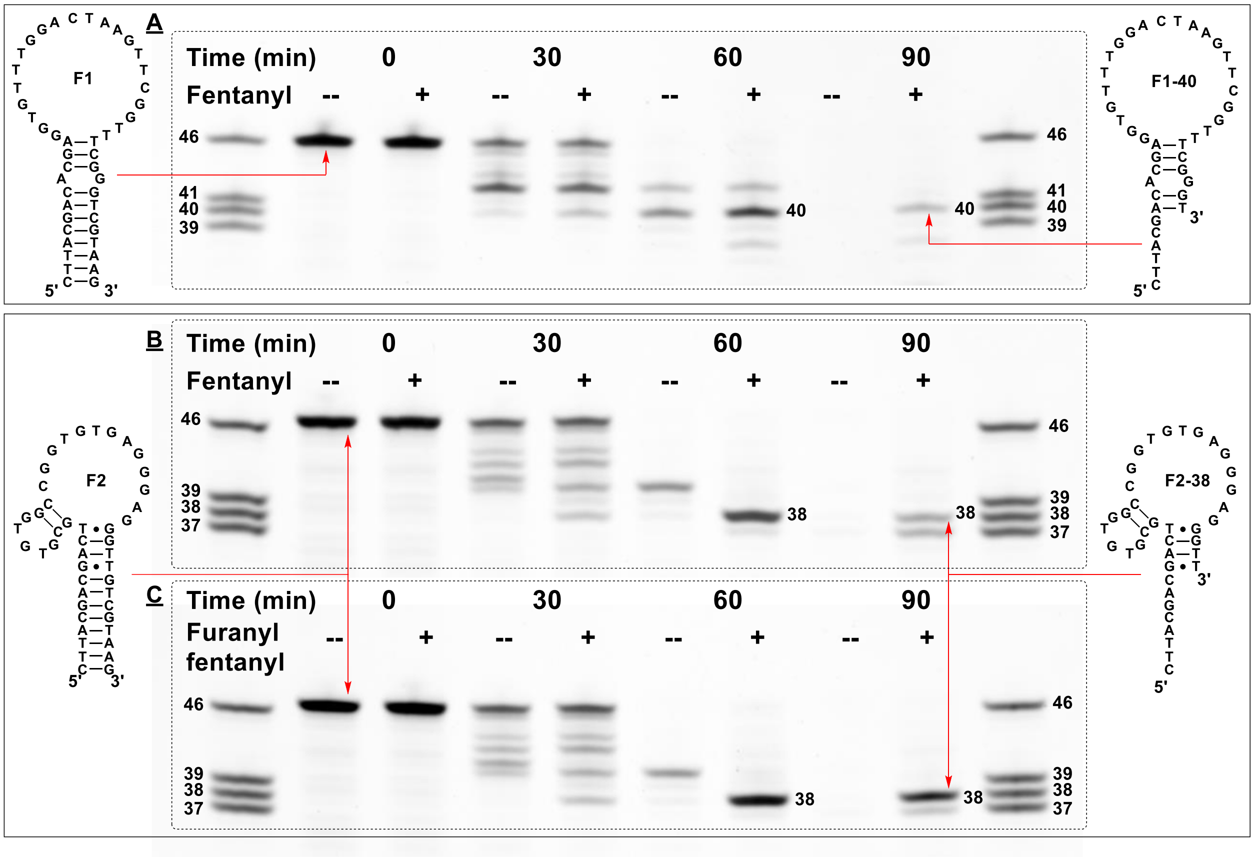

Figure S18. Identification of major inhibition products based on PAGE analysis of a time-course digestion of (**A**) F1 with fentanyl, (**B**) F2 with fentanyl, and (**C**) F2 with furanyl fentanyl. NUPACK(2)-predicted secondary structures of parent aptamers (left) and hypothetical structures of the major digestion products (right) are provided.

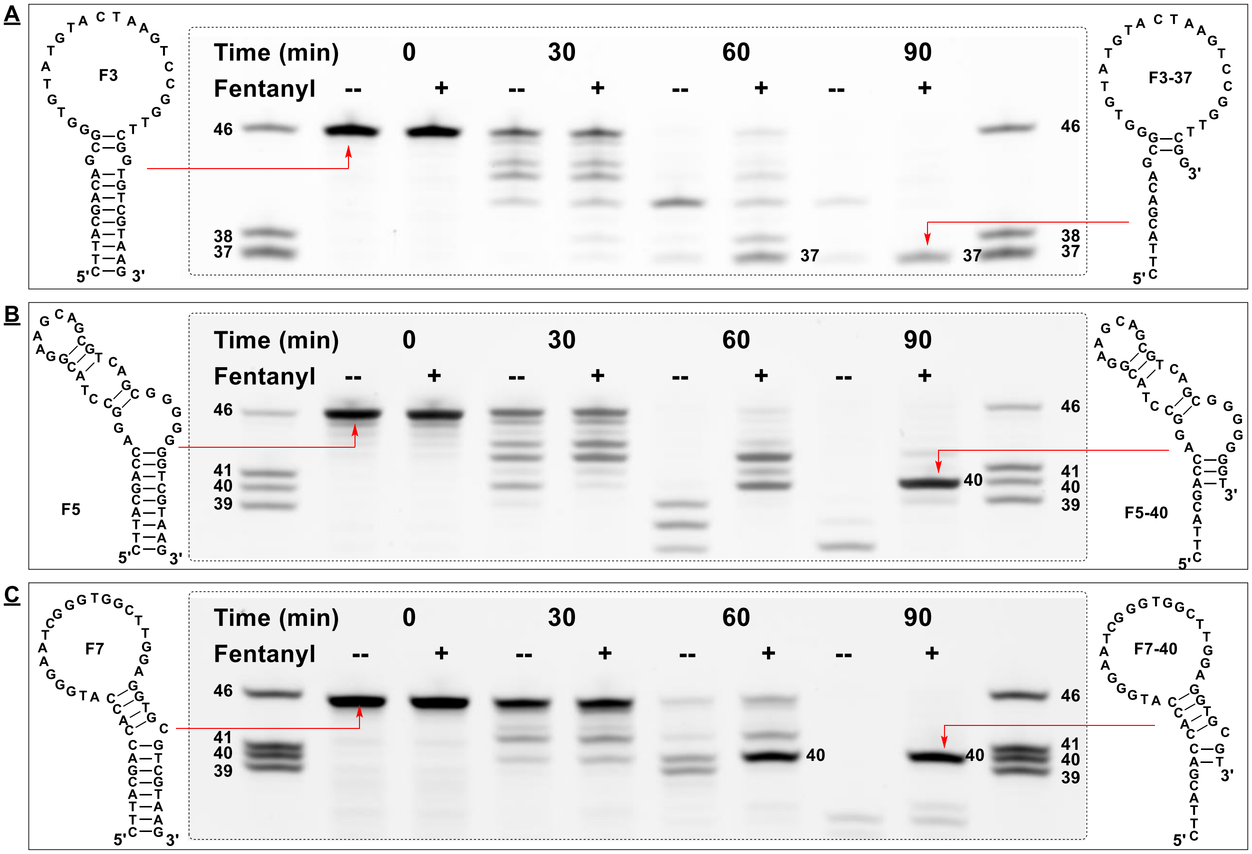

Figure S19. Identification of major inhibition products based on PAGE analysis of a time-course digestion of (**A**) F3 with fentanyl, (**B**) F5 with fentanyl, and (**C**) F7 with fentanyl. NUPACK(2)-predicted secondary structures of parent aptamers (left) and hypothetical structures of the major digestion products (right) are provided.

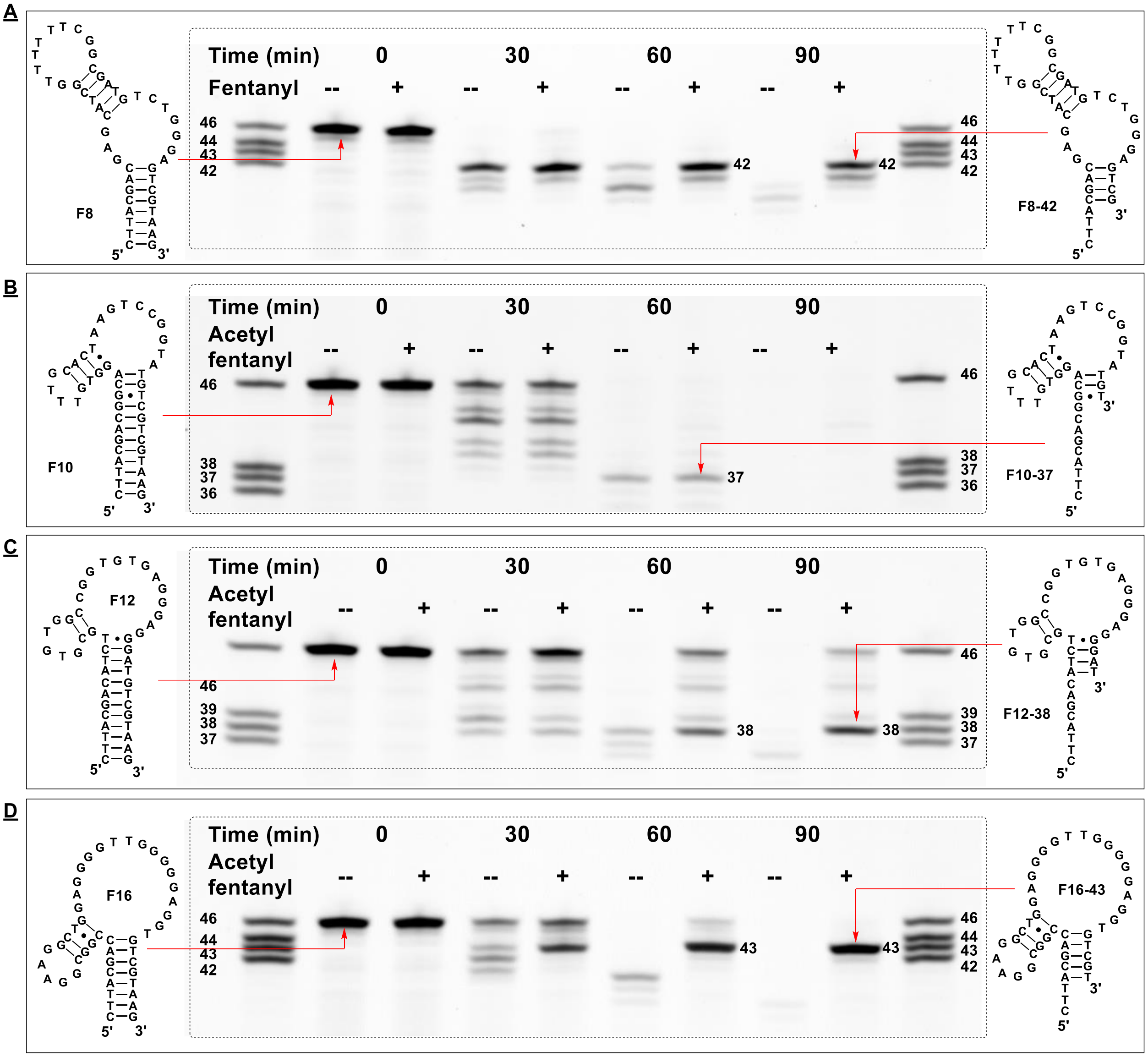

Figure S20. Identification of major inhibition product based on PAGE analysis of a time-course digestion of (**A**) F8 with fentanyl, and (**B**) F10, (**C**) F12, and (**D**) F16 with acetyl fentanyl. NUPACK(2)-predicted secondary structures of parent aptamers (left) and hypothetical structures of the major digestion products (right) are provided.

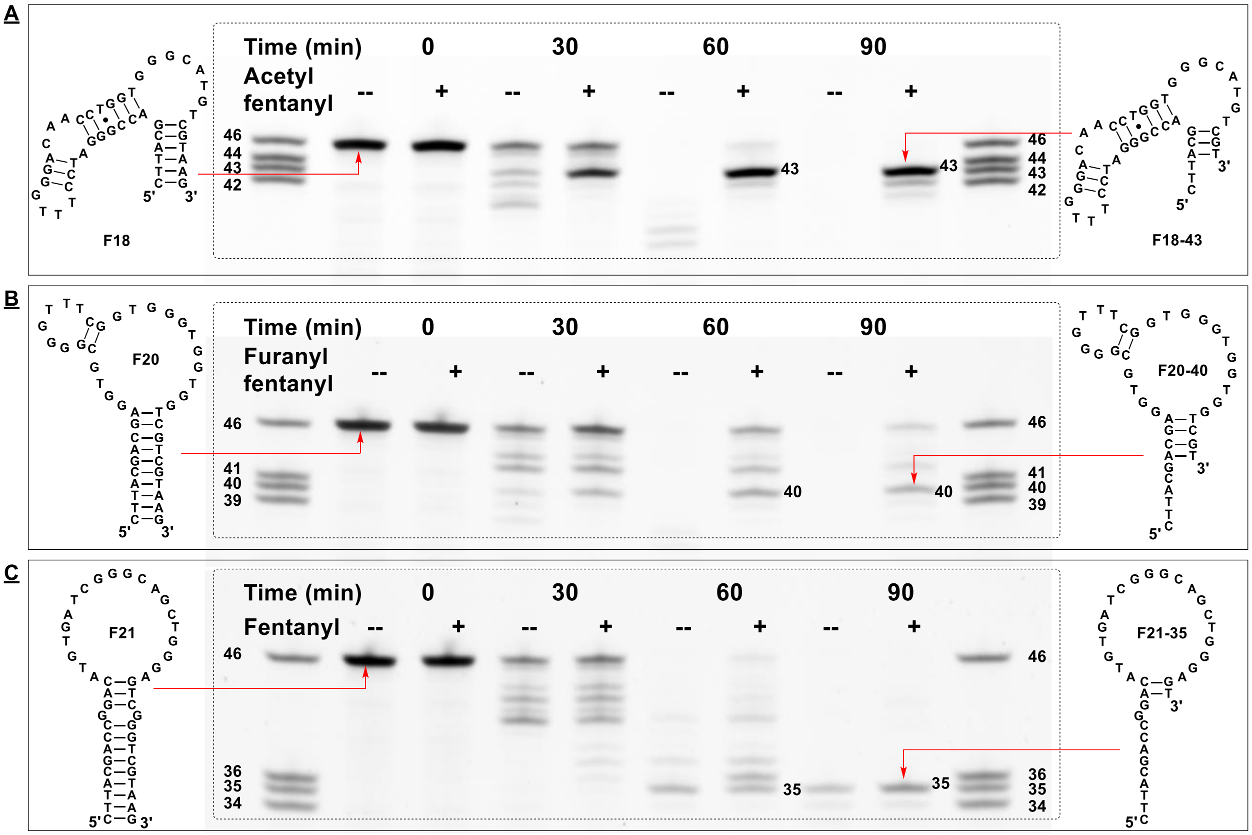

Figure S21. Identification of major inhibition products based on PAGE analysis of a time-course digestion of (**A**) F18 with acetyl fentanyl, (**B**) F20 with furanyl fentanyl, and (**C**) F21 with fentanyl. NUPACK(2)-predicted secondary structures of parent aptamers (left) and hypothetical structures of the major digestion products (right) are provided.

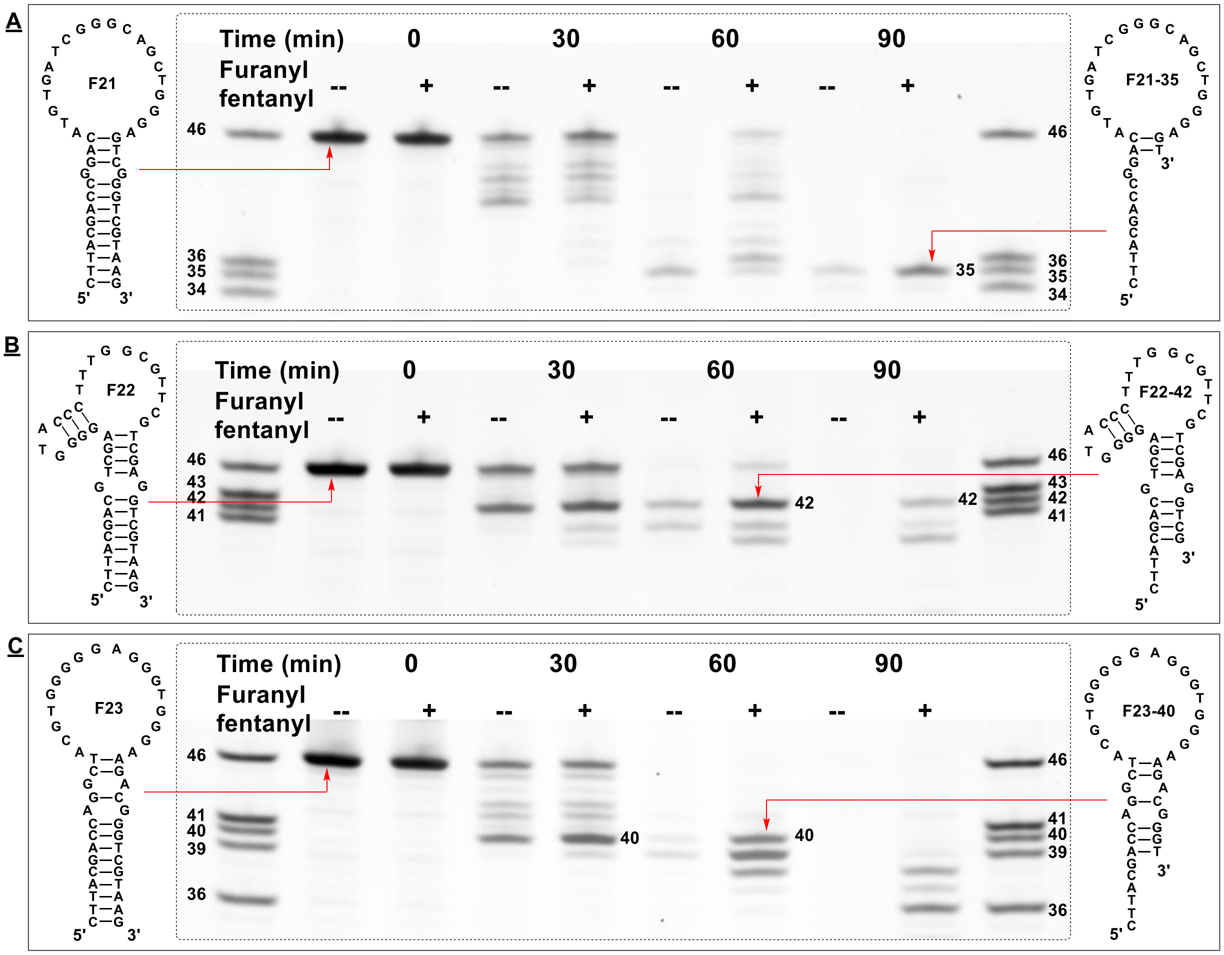

Figure S22. Identification of major inhibition products based on PAGE analysis of a time-course digestion of (**A**) F21, (**B**) F22, and (**C**) F23 with furanyl fentanyl. NUPACK(2)-predicted secondary structures of parent aptamers (left) and hypothetical structures of the major digestion products (right) are provided.

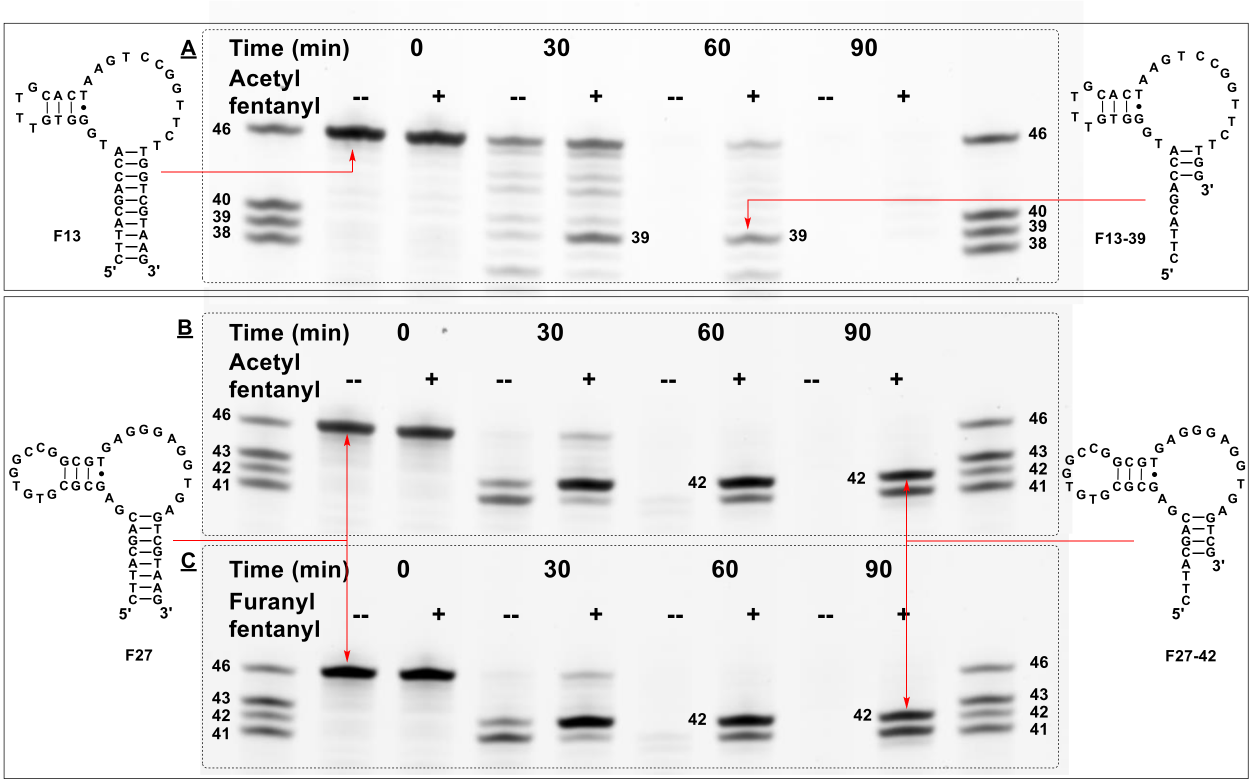

Figure S23. Identification of major inhibition products based on PAGE analysis of a time-course digestion of (**A**) F13 and (**B**) F27 with acetyl fentanyl and (**C**) furanyl fentanyl. NUPACK(2)-predicted secondary structures of parent aptamers (left) and hypothetical structures of the major digestion products (right) are provided.

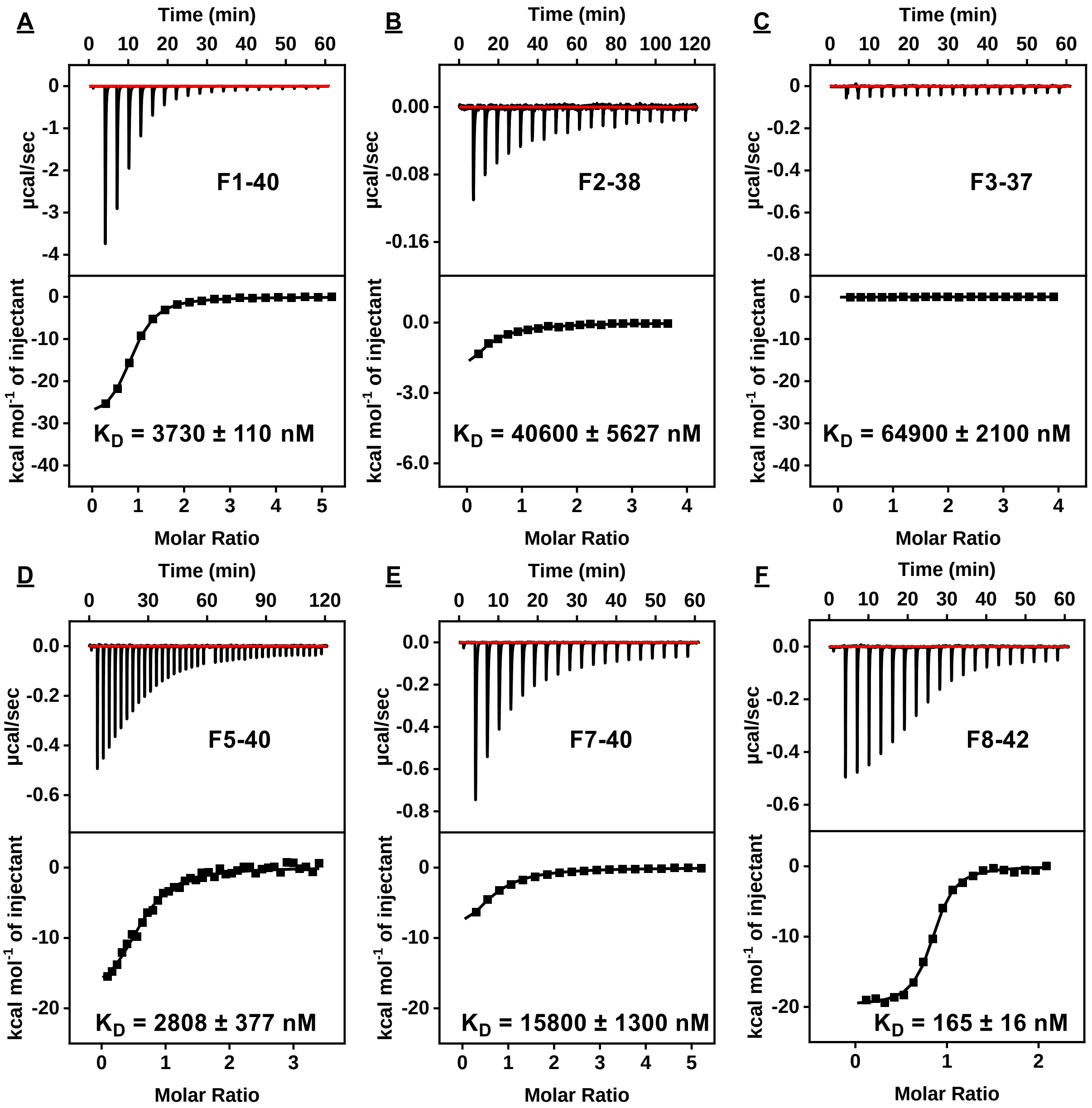

Figure S24. Characterization of fentanyl binding affinity of six major inhibition products using ITC. Top panels display the heat generated from each titration of fentanyl into (**A**) F1-40, (**B**) F2-38, (**C**) F3-37, (**D**) F5-40, (**E**) F7-40, and (**F**) F8-42. Bottom panels show the integrated heat of each titration after correcting for the heat of dilution of the titrant.

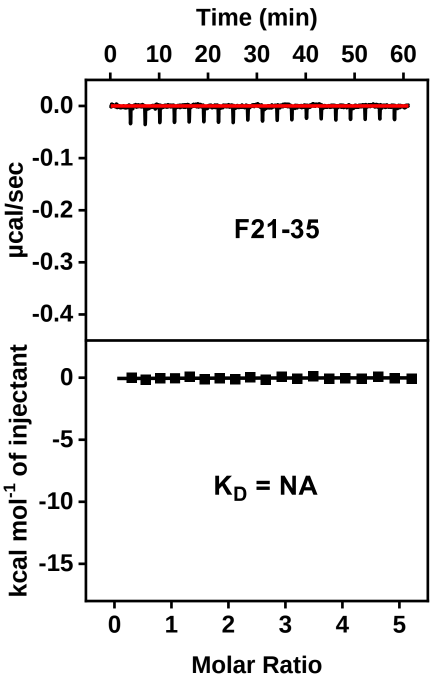

Figure S25. Characterization of fentanyl binding affinity of a major inhibition product of F21 using ITC. Top panels display the heat generated from each titration of fentanyl into F21-35. Bottom panels show the integrated heat of each titration after correcting for the heat of dilution of the titrant.

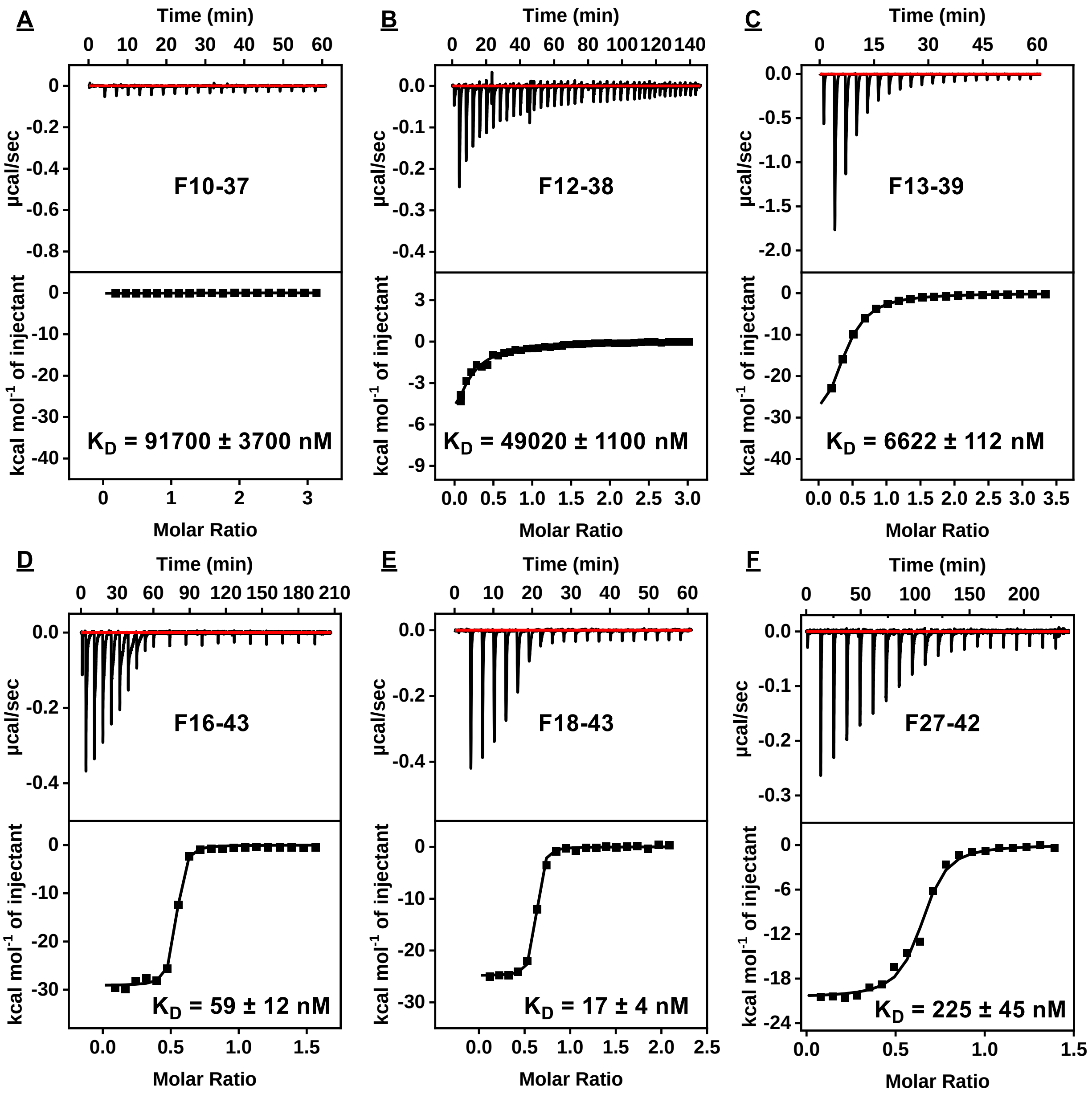

Figure S26. Characterization of acetyl fentanyl binding affinity of six major inhibition products using ITC. Top panels display the heat generated from the titration of fentanyl into (**A**) F10-37, (**B**) F12-38, (**C**) F13-39, (**D**) F16-43, (**E**) F18-43, and (**F**) F27-42. Bottom panels show the integrated heat of each titration after correcting for the heat of dilution of the titrant.

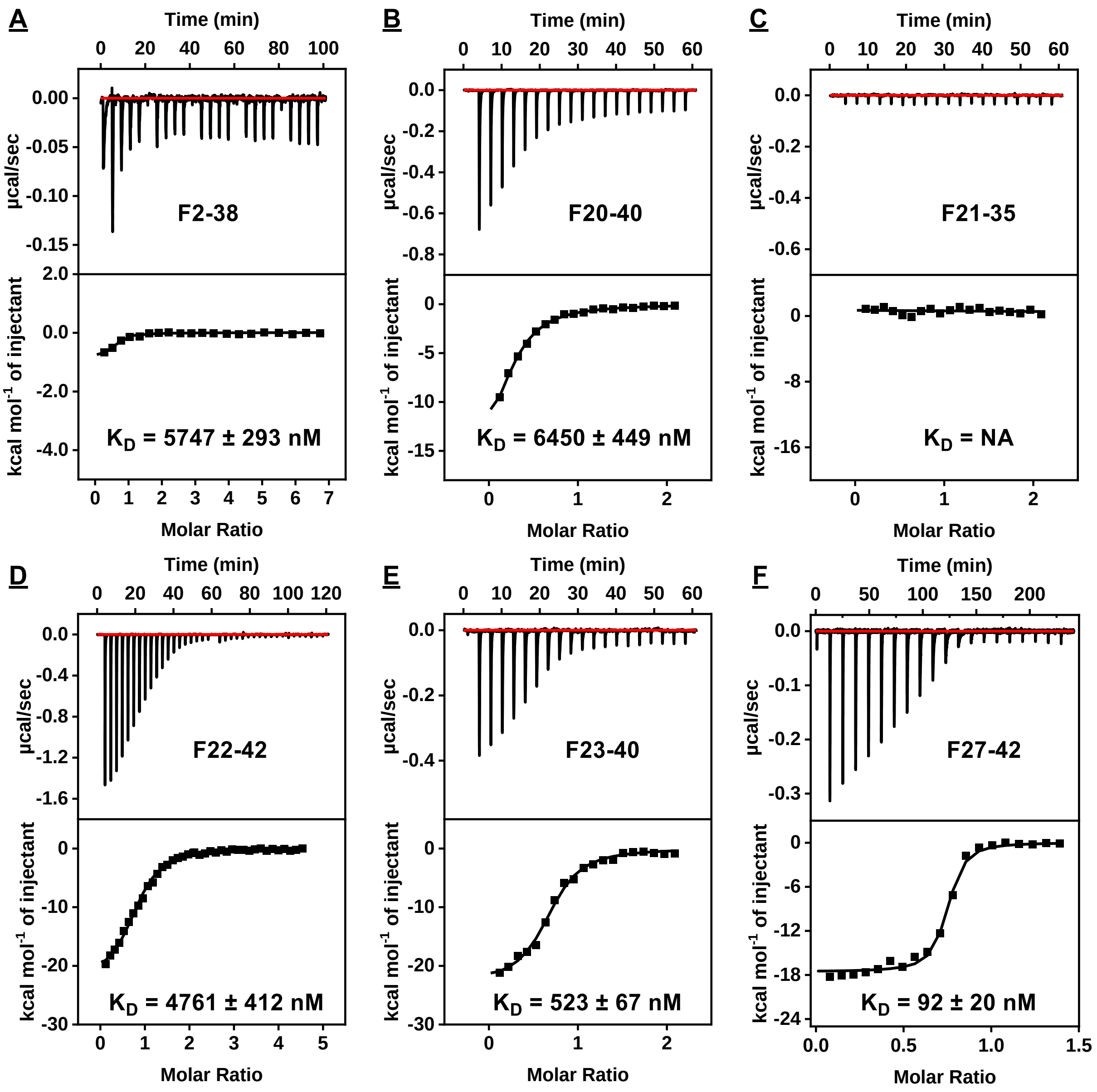

Figure S27. Characterization of furanyl fentanyl binding affinity of five major inhibition products using ITC. Top panels display the heat generated from each titration of furanyl fentanyl into (**A**)F2-38, (**B**) F20-40, (**C**) F21-35, (**D**) F22-42, (**E**) F23-40, and (**F**) F2-42. Bottom panels show the integrated heat of each titration after correcting for the heat of dilution of the titrant.

$$R_{value} = 1.6 - \frac{1.6 \times {K_D}^n}{{E_{1/2}}^n + {K_D}^n}$$

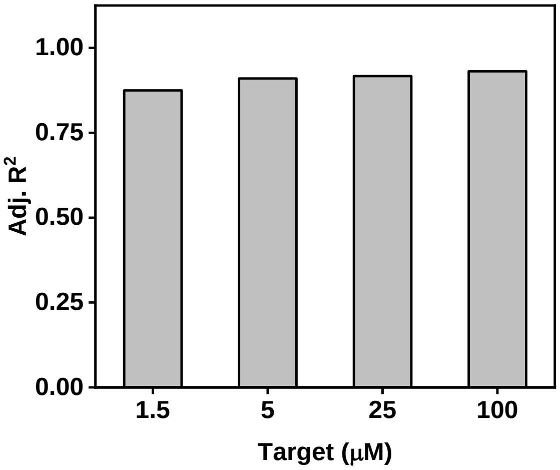

Figure S28. Equation used for fitting (top) and adjusted R^2 values (bottom) obtained during fitting of parent aptamer/major digestion product K_D against R_{value} obtained at 1.5, 5, 25, and 100 µM selection target concentrations. $E_{1/2}$ represents the K_D at which half-maximal inhibition is observed, and n represents the steepness of target response.

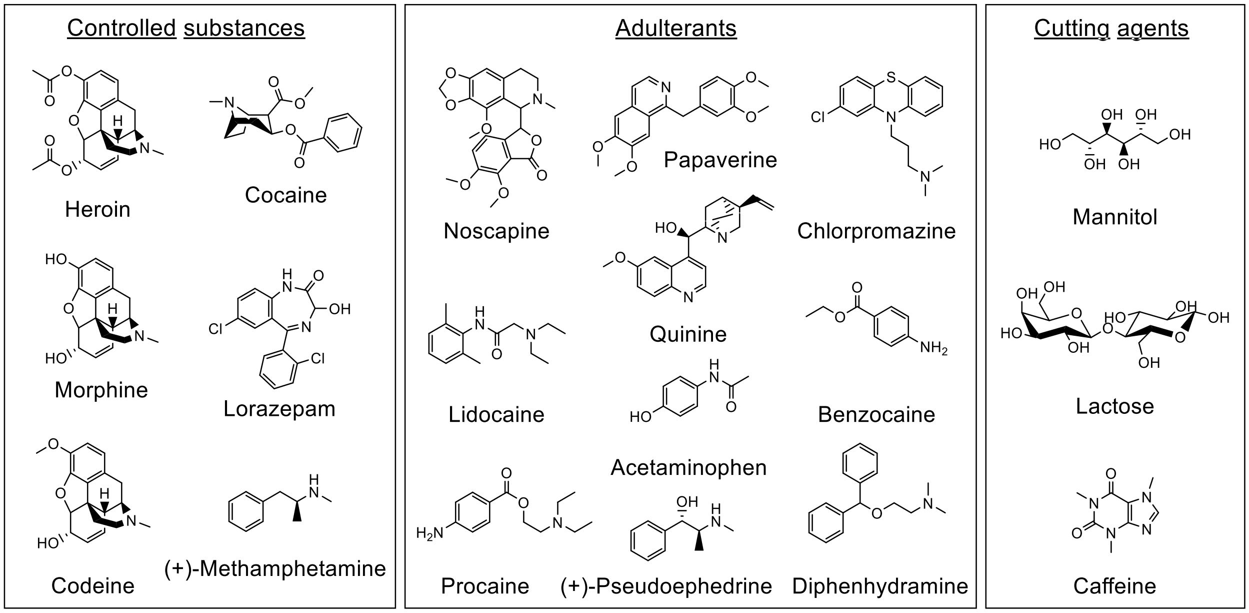

Figure S29. Chemical structure of nineteen interferent molecules employed for aptamer specificity testing, including controlled substances, adulterants, and cutting agents.

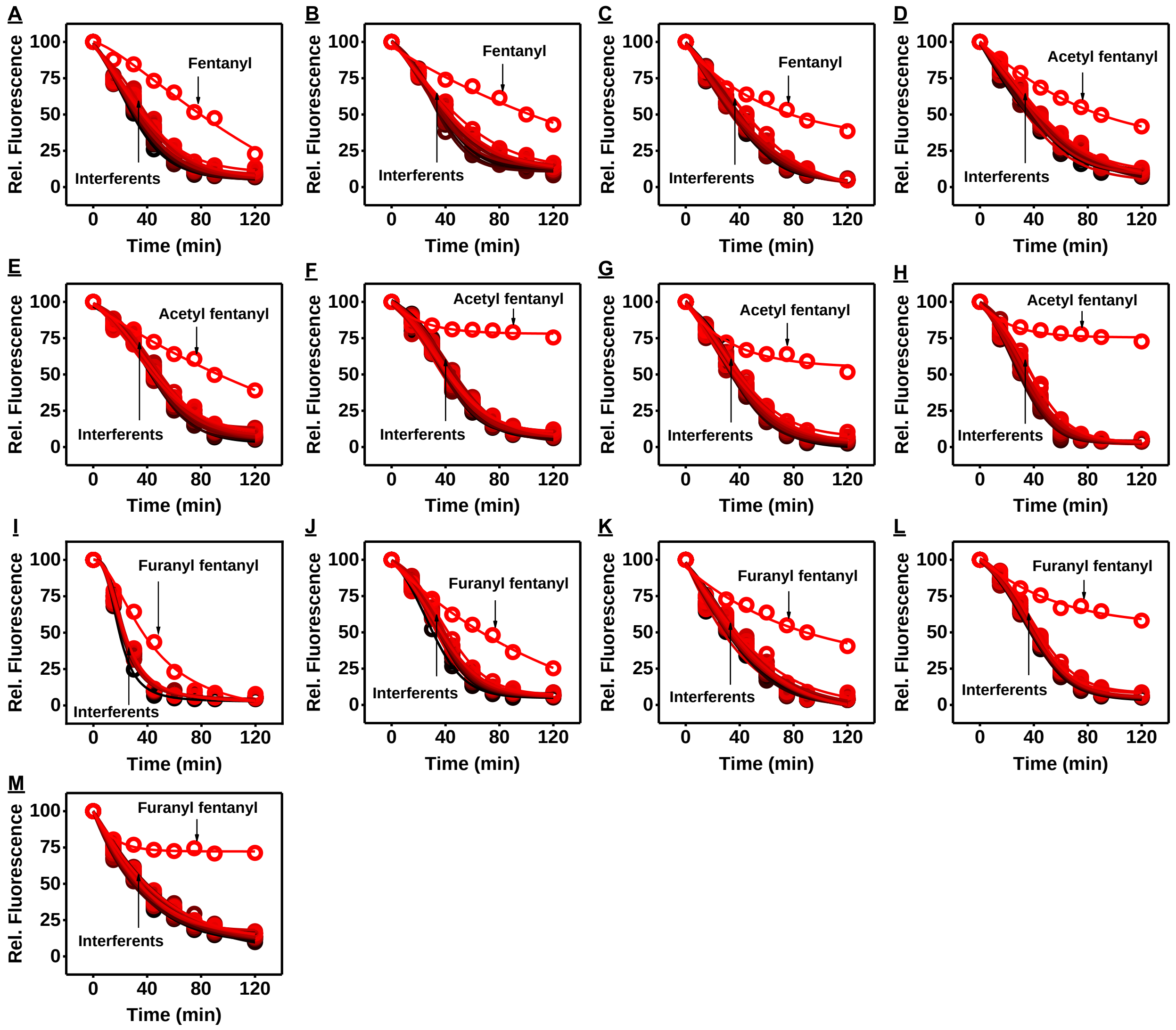

Figure S30. Digestion time-course digestion for (**A**) F4, (**B**) F5, (**C**) F6, (**D**) F12, (**E**) F13, (**F**) F14, (**G**) F17, (**H**) F18, (**I**) F20, (**J**) F23, (**K**) F24, (**L**) F25, and (**M**) F27 in the absence and presence of 100 µM cocaine, lidocaine, procaine, heroin, quinine, codeine, morphine, chlorpromazine, lactose, mannitol, caffeine, (+)-methamphetamine, (+)-pseudoephedrine, benzocaine, diphenhydramine, acetaminophen, papaverine, noscapine, lorazepam, or their selection target.

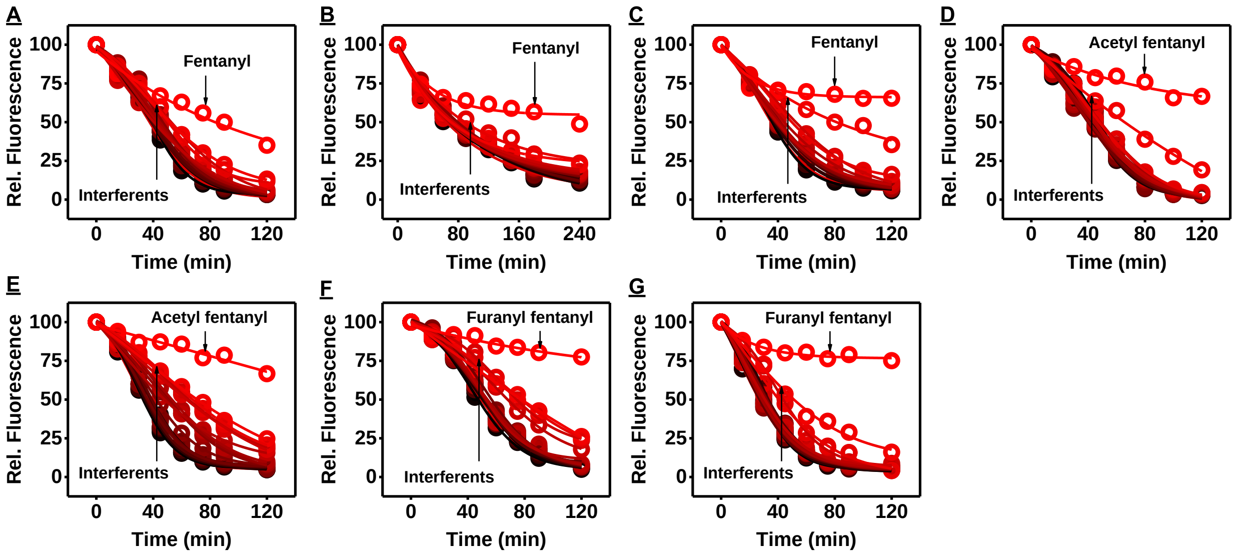

Figure S31. Digestion time-course digestion of (**A**) F7, (**B**) F8, (**C**) F9, (**D**) F15, (**E**) F16, (**F**) F26, and (**G**) F28 in the absence and presence of 100 μM cocaine, lidocaine, procaine, heroin, quinine, codeine, morphine, chlorpromazine, lactose, mannitol, caffeine, (+)-methamphetamine, (+)-pseudoephedrine, benzocaine, diphenhydramine, acetaminophen, papaverine, noscapine, lorazepam, or their selection target.

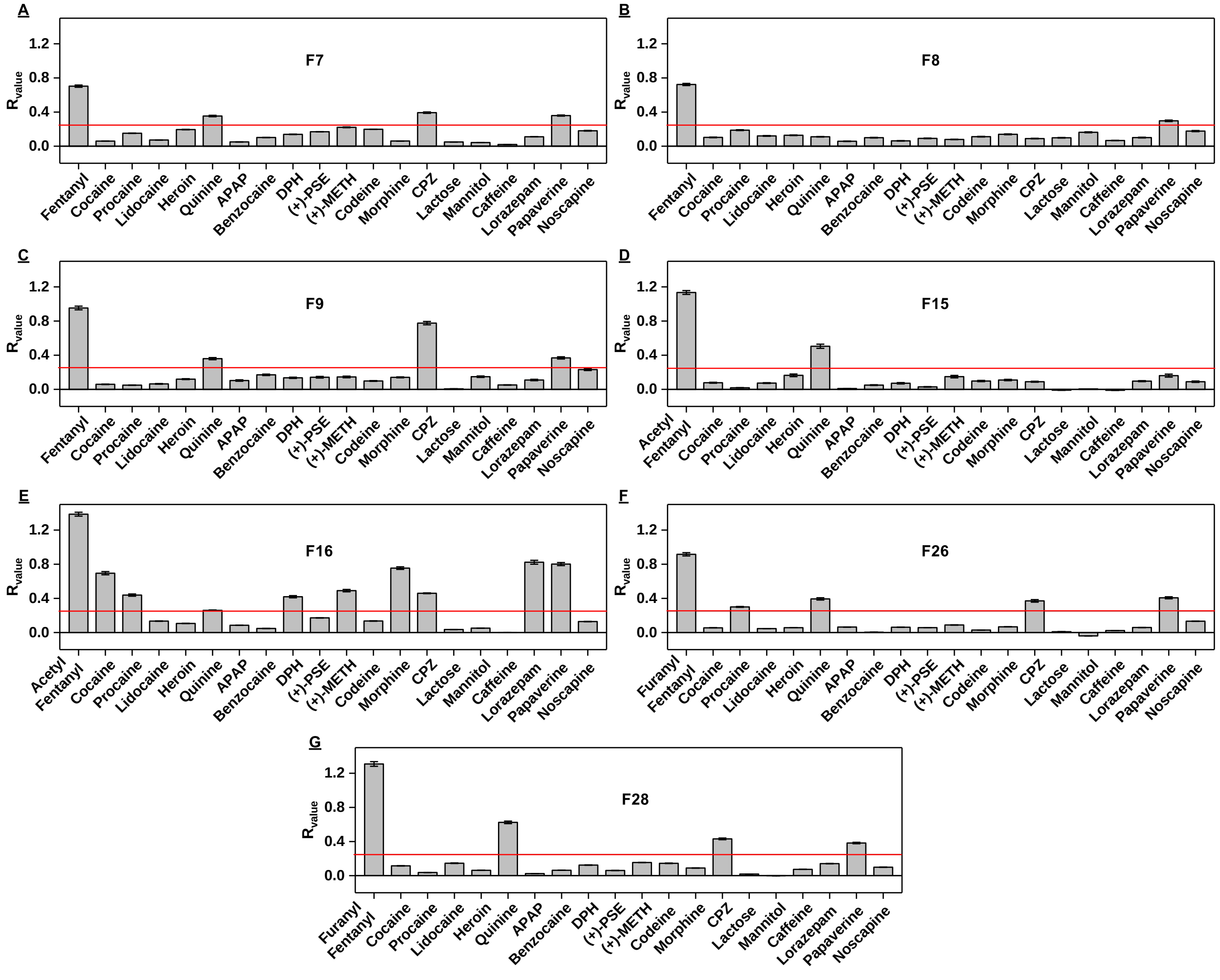

Figure S32. R_{value}s of (**A**) F7, (**B**) F8, (**C**) F9, (**D**) F15, (**E**) F16, (**F**) F26, and (**G**) F28 following exonuclease digestion with 100 µM selection target, cocaine, procaine, lidocaine, heroin, quinine, acetaminophen (APAP), benzocaine, diphenhydramine (DPH), (+)-pseudoephedrine ((+)-PSE), (+)-methamphetamine ((+)-METH), codeine, morphine, chlorpromazine (CPZ), lactose, mannitol, caffeine, lorazepam, papaverine, or noscapine. The red line indicates R_{value} = 0.25, demarcating the threshold indicating very weak binding.

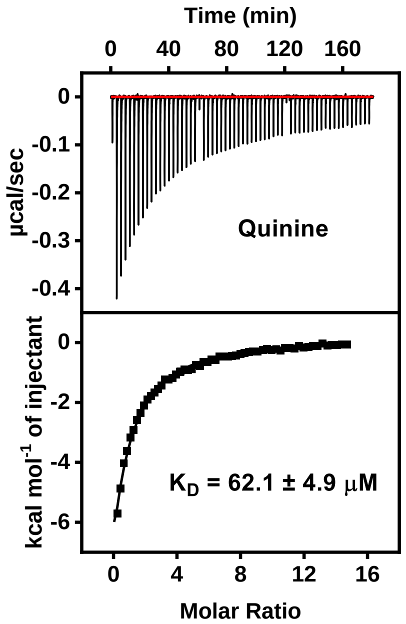

Figure S33. Characterization of quinine binding affinity of digestion product F7-40 using ITC. Top panels display the heat generated from each titration of quinine into F7-40. Bottom panels show the integrated heat of each titration after correcting for the heat of dilution of the titrant.

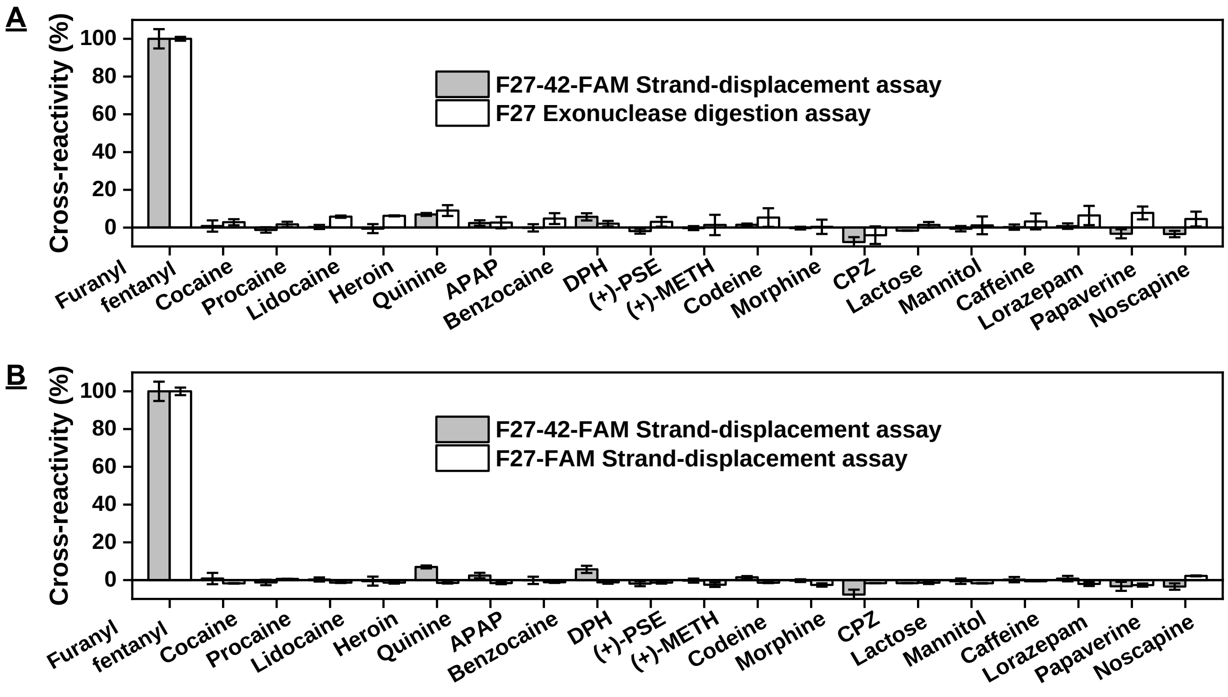

Figure S34. Comparison of aptamer specificity determined using strand-displacement fluorescence assay and exonuclease digestion assay. Cross-reactivity of (**A**) F27-42-FAM and F27, and (**B**) F27-42-FAM and F27-FAM against 100 µM selection target and our various interferents. Cross-reactivity was calculated relative to the signal generated by furanyl fentanyl target. Acetaminophen (APAP), diphenhydramine (DPH), (+)-pseudoephedrine ((+)-PSE), (+)-methamphetamine ((+)-METH), chlorpromazine (CPZ).

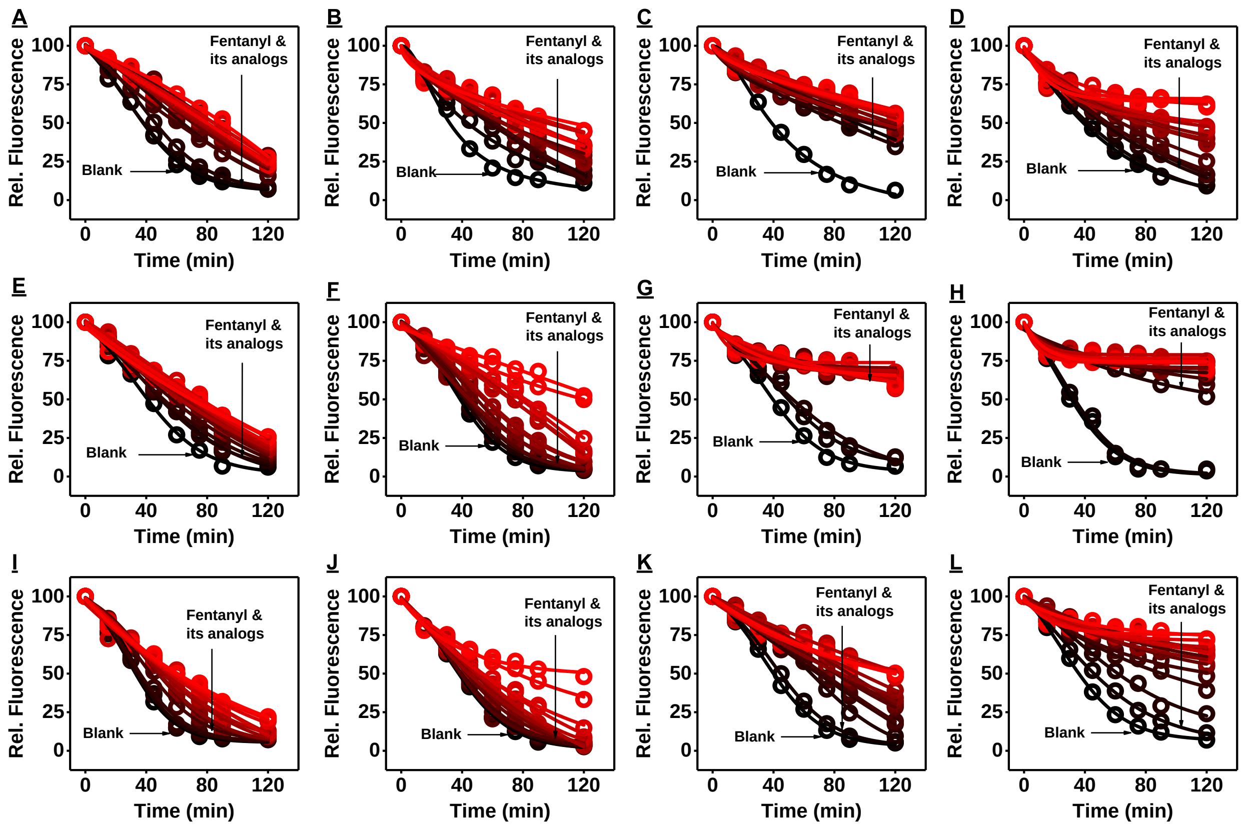

Figure S35. Screening cross-reactivity via time-course digestion of (**A**) F4, (**B**) F5, (**C**) F6, (**D**) F12, (**E**) F13, (**F**) F14, (**G**) F17, (**H**) F18, (**I**) F23, (**J**) F24, (**K**) F25, and (**L**) F27 in the absence and presence of 100 µM fentanyl and the 14 analogues shown in **Figure 1**.

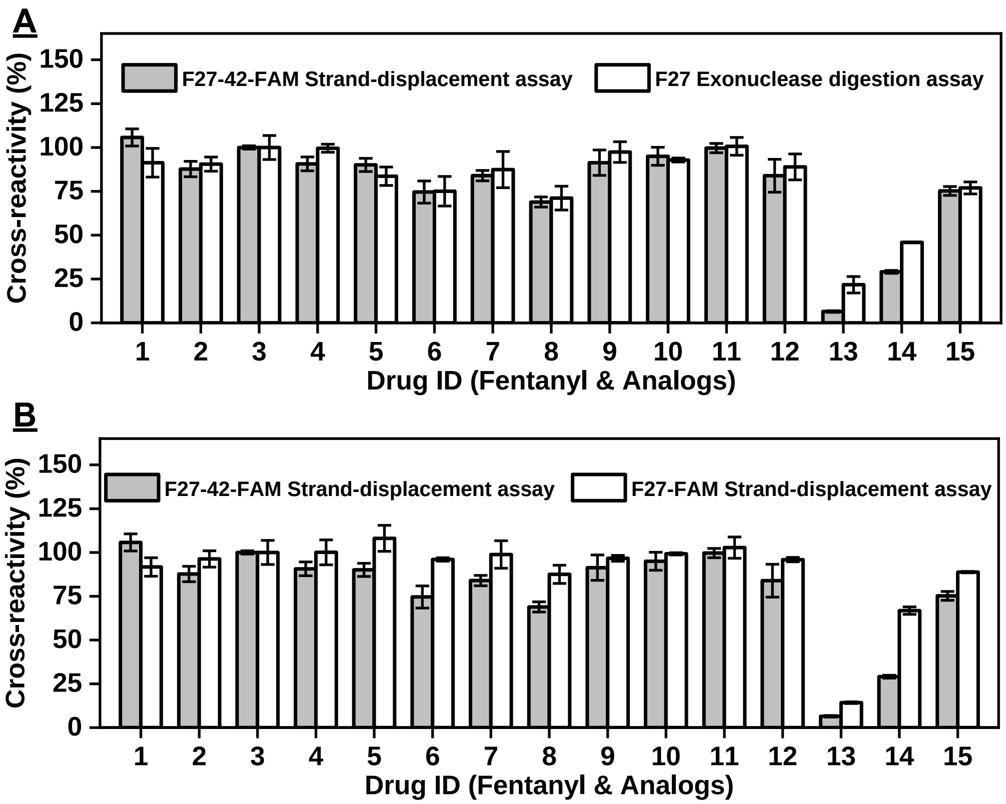

Figure S36. Comparison of cross-reactivity as determined using a fluorescence strand-displacement assay and exonuclease digestion assay for (**A**) F27-42-FAM and F27, respectively, (**B**) a strand-displacement fluorescence assay for F27-42-FAM and F27-FAM against 100 µM fentanyl and 14 of its analogs. Cross-reactivity was calculated based on the signal produced by acetyl fentanyl for F13 or furanyl fentanyl for F27 and F27-42.

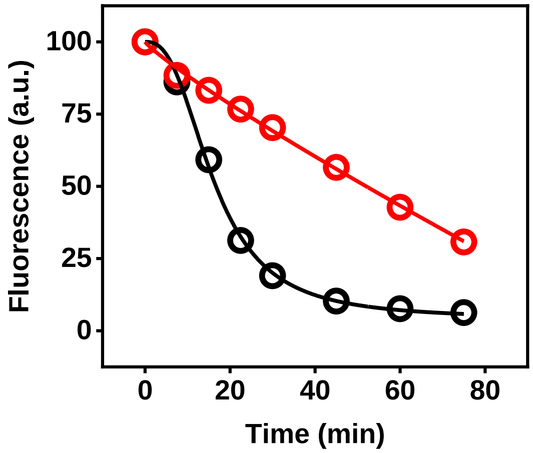

Figure S37. Digestion time-course of F27 in the absence and presence of 5 µM fentanyl.

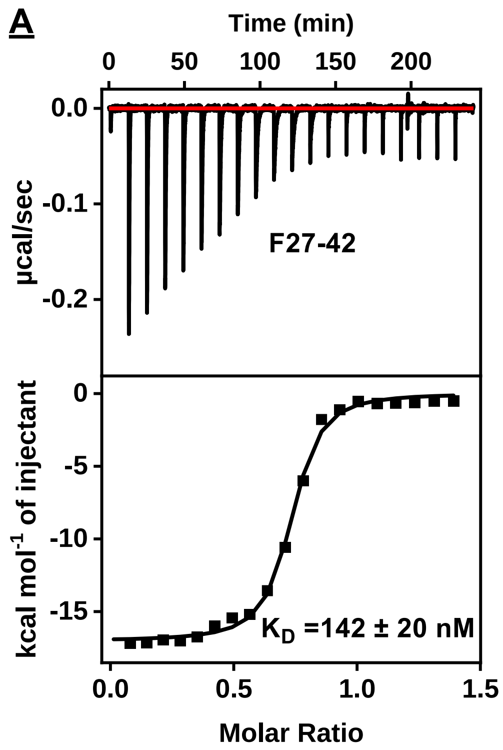

Figure S38. Characterization of fentanyl affinity of (**A**) F27-42 and (**B**) F13-39 using ITC. Top panels display the heat generated from each titration of fentanyl into F27-42 and F13-39. Bottom panels show the integrated heat of each titration after correcting for the heat of dilution of the titrant.

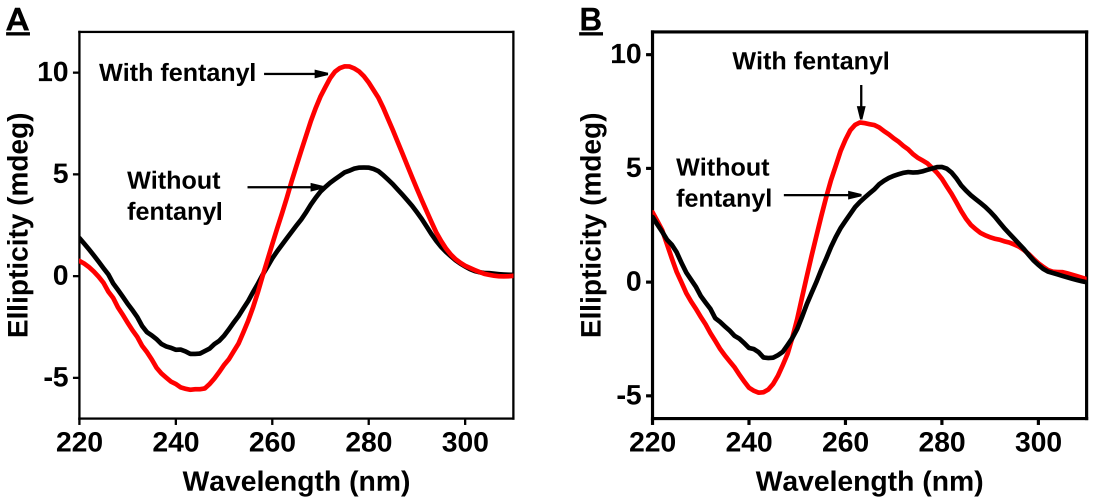

Figure S39. Confirmation of structure-switching functionality for (**A**) F13-39 and (**B**) F27-42 based on circular dichroism spectra in the absence (black) and presence (red) of 10 µM fentanyl.

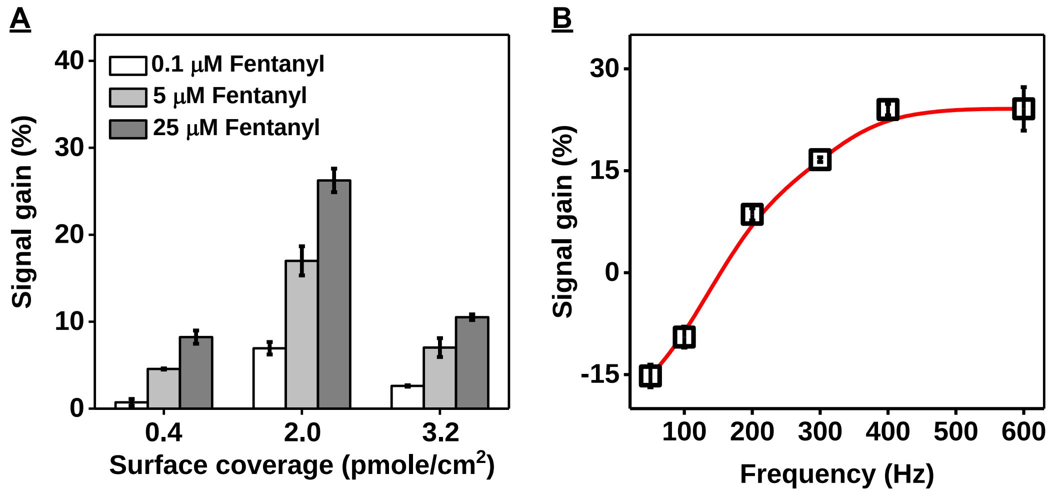

Figure S40. Optimization of (**A**) surface coverage of E-AB sensors constructed with F27-38-MB for detection of 0.1, 5 and 25 µM fentanyl, and (**B**) performance of E-AB sensors at different frequencies for detection of 5 µM fentanyl.

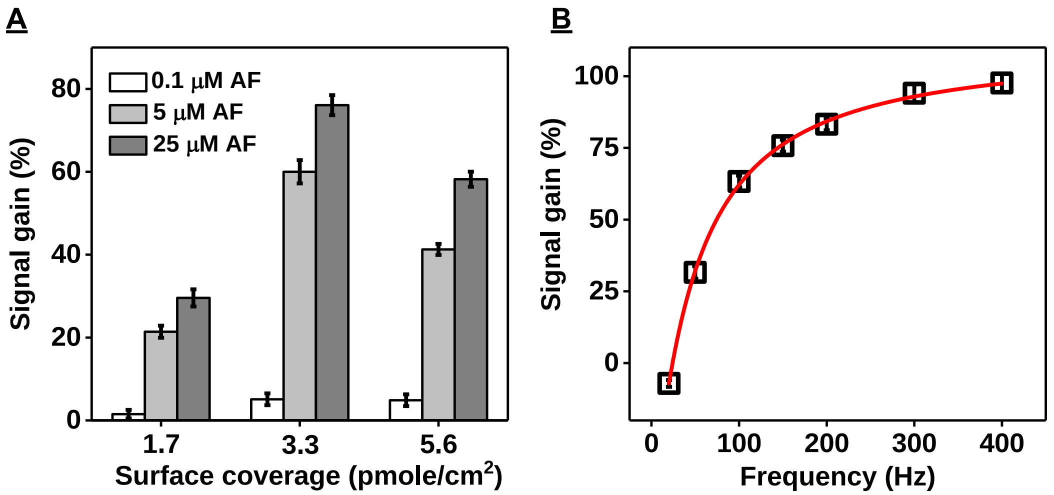

Figure S41. Optimization of (**A**) surface coverage of E-AB sensors constructed with F13-32-MB for detection of 0.1, 5 and 25 µM acetyl fentanyl, and (**B**) performance of E-AB sensors at different frequencies for detection of 50 µM acetyl fentanyl.

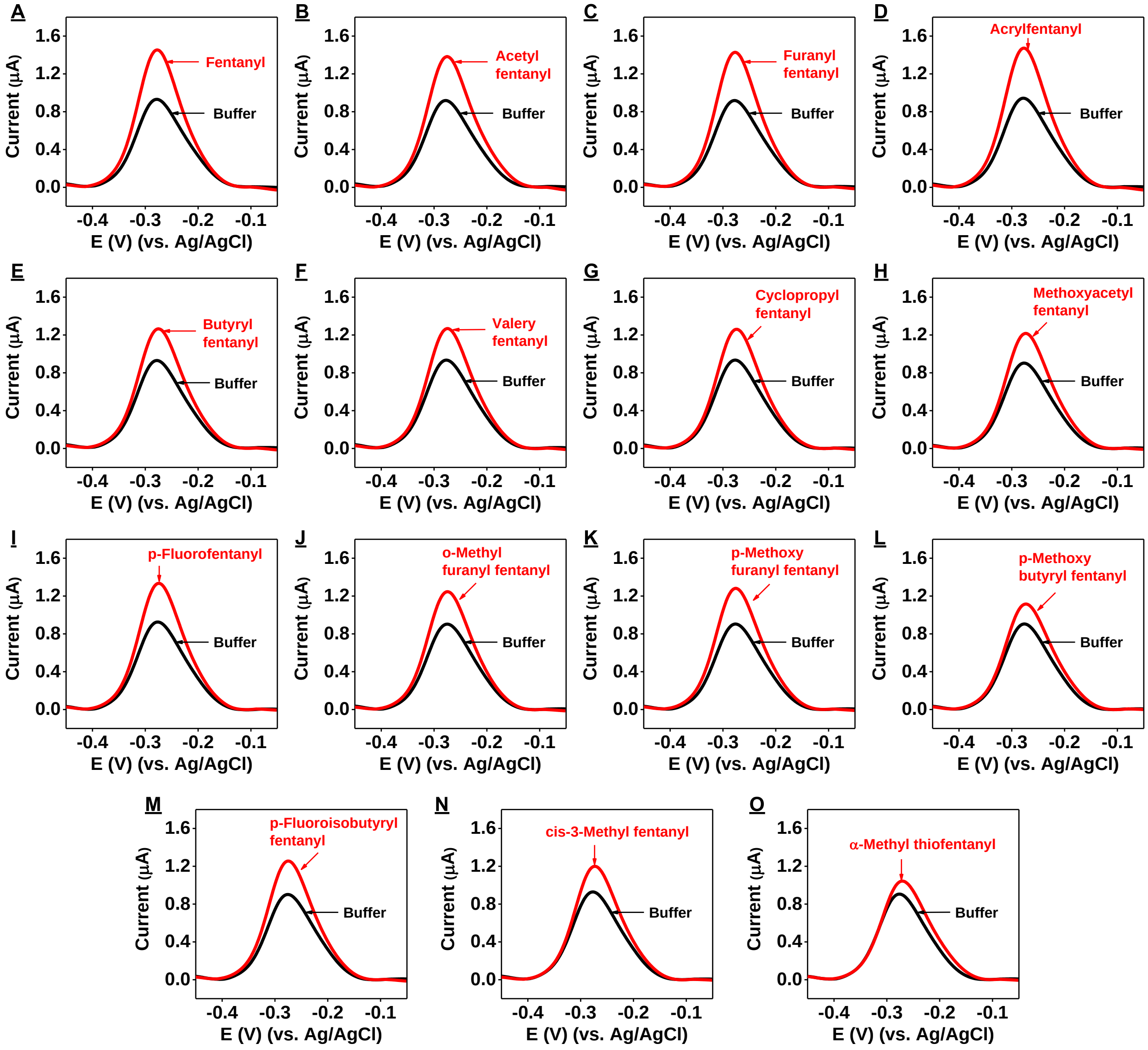

Figure S42. SWV response of an E-AB sensor constructed with F13-32-MB to fentanyl and its 14 analogs at a concentration of 5 µM.

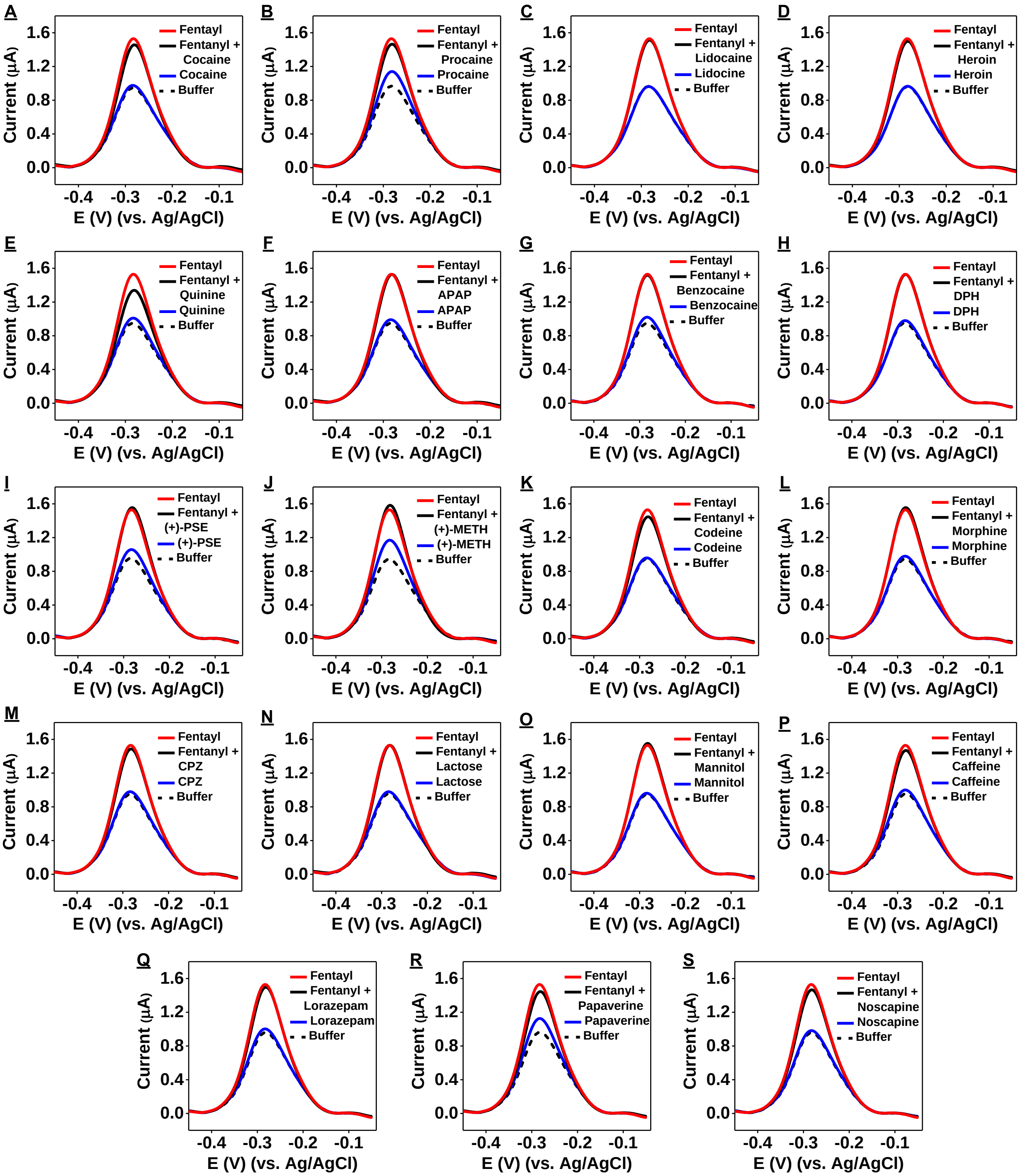

Figure S43. SWV response of F13-32-MB E-AB sensor to binary mixtures containing a 1:100 or 1:40 molar ratio of fentanyl (final concentration 5 µM) and various interferents commonly found in seized substances (200 µM papaverine, noscapine, and lorazepam; 500 µM other interferents).

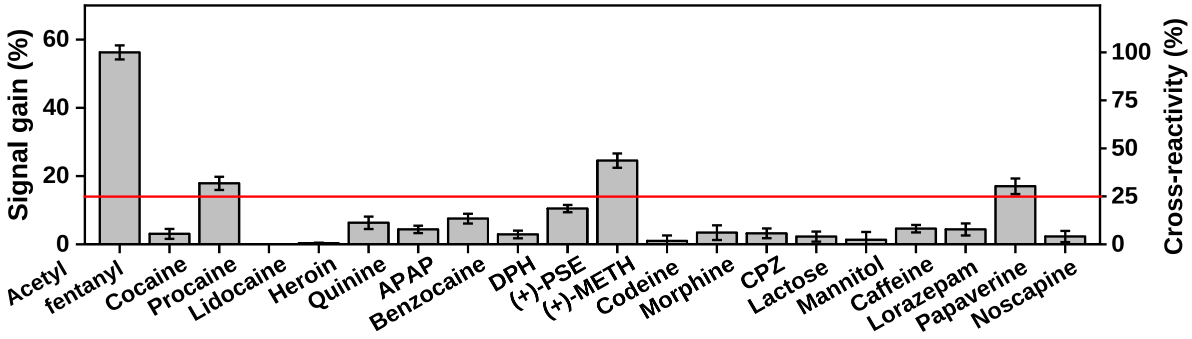

Figure S44. Signal gain and cross-reactivity determined using F13-32-MB E-AB sensor. Cross-reactivity was calculated against 100 μM selection target and various interferents.

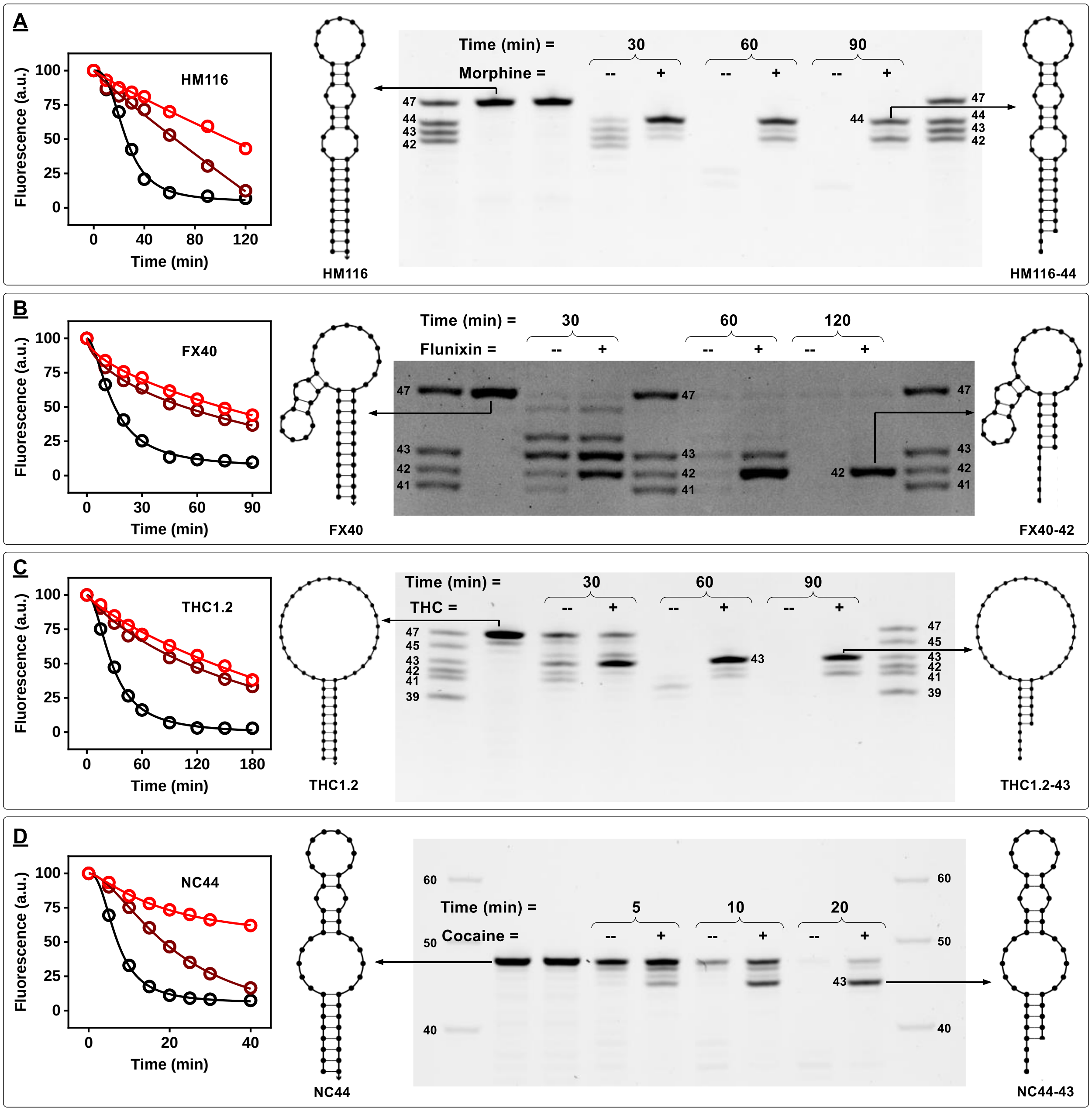

Figure S45. Exonuclease digestion time-course for aptamers newly isolated by our group, including (**A**) morphine-binding aptamer HM116, (**B**) flunixin-binding aptamer FX40, (**C**) THC-binding aptamer THC1.2, and (**D**) cocaine-binding aptamer NC44. Leftmost panel shows fluorescence change without (black) and with 10 µM (brown) or 100 µM (red) target. Gel images show major inhibition products based on PAGE analysis of time-course digestion and are flanked by NUPACK-predicted secondary structures of parent aptamers (left) and hypothetical structures of the major digestion products (right).

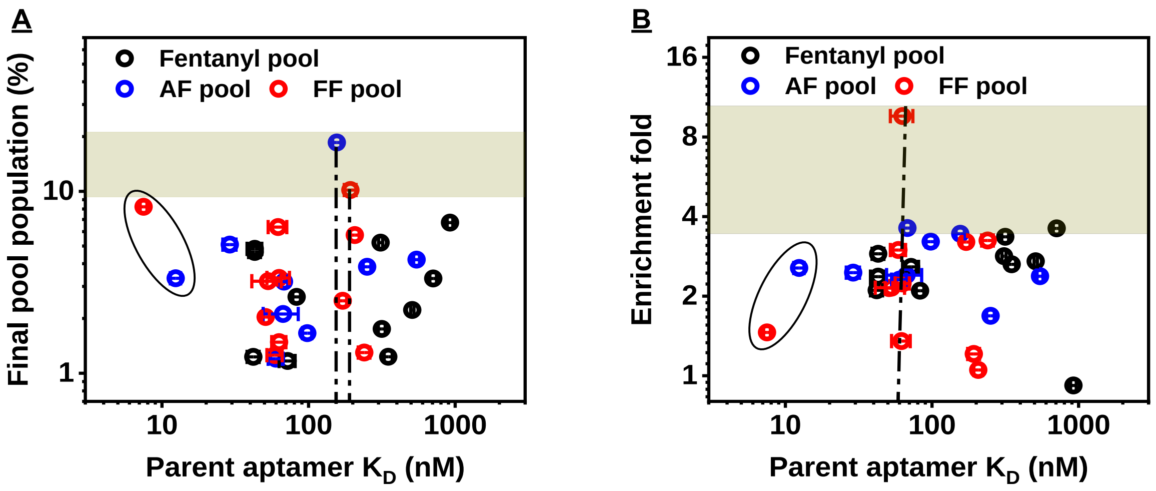

Figure S46. Correlation between parent aptamer binding affinity and performance based on (**A**) abundance in the final pool and (**B**) enrichment between early and final rounds. The aptamers with the highest affinity are circled. The shaded area respectively indicates the most abundant or highly enriched sequences.

References

(1) Yu,H., Yang,W., Alkhamis,O., Canoura,J., Yang,K.-A., Xiao,Y. (2018) *In Vitro* Isolation of Small-Molecule-Binding Aptamers with Intrinsic Dye-Displacement Functionality. *Nucleic Acids Res.*, **46**, e43.

(2) Zadeh,J.N., Steenberg,C.D., Bois,J.S., Wolfe,B.R., Pierce,M.B., Khan,A.R., Dirks,R.M., Pierce,N.A. (2011) NUPACK: Analysis and Design of Nucleic Acid Systems. *J. Comput. Chem.*, **32**, 170–173.
